# Theoretical insight into the effect of CO coverage on formic acid formation from dissociated oxygen on χ-Fe_5_C_2_(510) in Fischer–Tropsch synthesis

**DOI:** 10.1039/d6ra04509j

**Published:** 2026-07-23

**Authors:** Jinchun Jiang, Hongzhi Zheng, Changyi Lai, Qining Wang, Jie Ren, Ning Ai, Wei Zhou

**Affiliations:** a College of Biological Chemical Science and Engineering, Jiaxing University Jiaxing China; b National Demonstration Center for Experimental Chemistry and Chemical Engineering Education, Zhejiang University of Technology Hangzhou China; c Sichuan Technology and Business University Chengdu China weixiaoba0328@163.com

## Abstract

The removal of dissociated oxygen in the form of formic acid over iron-based catalysts can effectively improve atomic utilization efficiency during the Fischer–Tropsch synthesis process. However, surface OH species tend to react with adsorbed hydrogen to generate H_2_O, which severely hinders formic acid formation on the χ-Fe_5_C_2_(510) facet—the key active facet of iron-based catalysts. This work proposes a strategy to promote formic acid formation by increasing CO coverage on this facet, thereby enhancing the reaction probability between CO and OH intermediates. First, the adsorption energies of CO at different coverage degrees are investigated on the hydrogen-covered χ-Fe_5_C_2_(510) facet. The calculated Gibbs adsorption energies are −1.40 eV, −1.00 eV, and −0.30 eV for 1–3, 4–9, and more than 10 adsorbed CO molecules, respectively. Combined with the effective reaction barriers of 0.94 eV for CO activation and 0.73 eV for formic acid formation, the co-coverage model with 9 adsorbed CO molecules is identified as the most thermodynamically and kinetically favorable configuration for the CO/H co-covered χ-Fe_5_C_2_(510) facet. Furthermore, microkinetic data reveal that high CO coverage alters the optimal reaction pathway for formic acid synthesis, following the elementary reaction sequence: O + H → OH + CO → COOH + H → HCOOH. Under high CO coverage, the effective reaction barrier of the dominant formic acid formation pathway increases from 0.94 eV to 1.24 eV. In contrast, the energy barriers of competing side reactions for H_2_O and CO_2_ formation rise more remarkably, increasing from 0.74 eV to 1.26 eV and from 0.85 eV to 1.27 eV, respectively. This kinetic regulation substantially suppresses competitive side reactions and facilitates the elimination of surface dissociated oxygen *via* formic acid generation. Subsequent kinetic Monte Carlo (kMC) simulations successfully reproduce the formation of formic acid on the CO/H co-covered χ-Fe_5_C_2_(510) facet, verifying the feasibility and effectiveness of the CO coverage regulation strategy. The theoretical simulation results of this work provide fundamental insights and rational design guidance for the development of advanced Fischer–Tropsch synthesis catalysts with formic acid co-production capability.

## Abstract 2

DFT-based studies reveal that tuning the H_2_/CO ratio can effectively adjust CO coverage and thereby facilitate the removal of dissociated oxygen in the form of formate.

## Graphical abstract

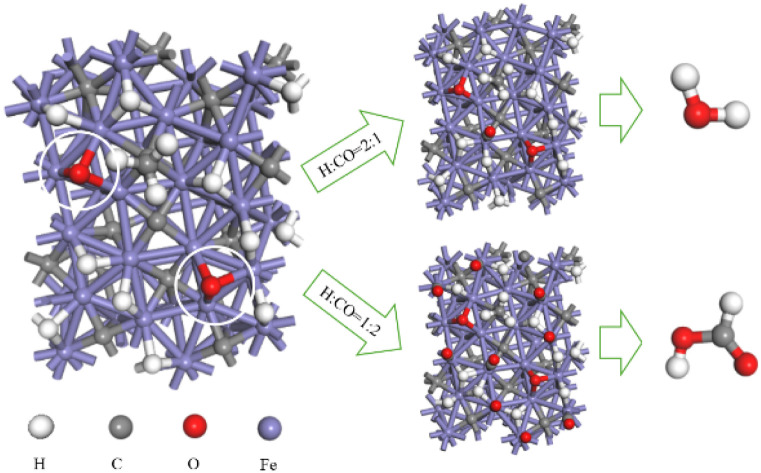

## 1. Introduction

Fischer–Tropsch synthesis (FTS) enables the efficient transformation of syngas into high-value hydrocarbons and oxygenated chemicals.^1–6^ The dissociation of interfacial C–O bonds are the key part within the Fischer–Tropsch reaction network.^7–9^ Oxygen species released from C–O bond tend to accumulate on the catalyst surface and are eventually consumed to form low-value byproducts, including CO_2_ and H_2_O, which severely deteriorates the atomic utilization efficiency and overall economic performance of practical FTS processes. In this regard, the directional conversion of adsorbed dissociated oxygen into value-added oxygenates is regarded as a feasible strategy to promote resource utilization efficiency of FTS. Based on the principle of atomic conservation, ideal byproduct must structurally integrate one carbon atom with two oxygen atoms, which can synchronously consume oxygen originating from reactant CO and remove excess surface oxygen residues. Typical candidates satisfying such structural characteristics primarily cover formic acid and oxalic acid. Notably, formic acid inherently exists as a trace oxygenated byproduct in FTS reactions^10,11^ making it a most feasible and targeted platform compound for realizing the high-value byproduct of surface dissociated oxygen species.

The χ-Fe_5_C_2_(510) facet is the dominant exposed surface among the active phases of iron-based catalysts and acts as the primary enrichment site for dissociated oxygen.^12–14^ On this surface, dissociated oxygen is mainly removed in the forms of CO_2_ and H_2_O.^15–18^ Our previous studies have demonstrated that dissociated oxygen can generate formic acid *via* the reaction pathway: O + H → OH + CO → COOH + H → HCOOH. The reaction barrier energy of this pathway is approximately 0.936 eV, which is comparable to that of the C–O activation process. Nevertheless, in the above reaction route, OH species tend to react with adsorbed hydrogen to form H_2_O,^19^ thereby severely inhibiting formic acid formation. As the surface coverage of reactants on the catalyst exerts a significant influence on the product distribution,^20–22^ to realize the removal of dissociated oxygen in the form of formic acid, increasing the surface CO coverage of the catalyst is proposed. By enabling CO to occupy more sites on the catalyst surface, the coverage of hydrogen can be reduced and the contact between OH and CO is facilitated to promote the formation of COOH intermediates, which ultimately drives the production of HCOOH. Adjusting the carbon-hydrogen ratio of feed gas is the way to adjust the surface coverage. However, changes in the partial pressure of CO and feed gas composition inevitably alter the phase structure of iron-based catalysts, making it experimentally unfeasible to conduct single-variable research that solely increases CO coverage.^23,24^ Therefore, this work adopts theoretical simulation methods to verify the feasibility of promoting HCOOH generation by elevating surface CO coverage.

This study would construct χ-Fe_5_C_2_(510) models with different CO coverages and the structural stability with various coverage configurations is evaluated to establish reliable CO/H co-coverage χ-Fe_5_C_2_(510) model. After that, the reaction network for dissociated oxygen removal is designed, the transition state search is performed to acquire the kinetic parameters of each elementary reaction in the overall reaction pathway, and the dominant reaction routes for dissociated oxygen removal on the co-covered surface are thereby determined. Furthermore, the obtained reaction pathways are compared with those on χ-Fe_5_C_2_(510) models without CO coverage in previous literature, to clarify the effect of CO coverage on the kinetic characteristics of elementary reactions. Finally, kinetic Monte Carlo (kMC) simulation is adopted to analyse the reaction frequency of major products derived from dissociated oxygen on CO/H co-coverage χ-Fe_5_C_2_(510) model, and aims to theoretically verify that increasing CO coverage on the χ-Fe_5_C_2_(510) surface can effectively facilitate formic acid (HCOOH) formation.

This study offers theoretical guidance for the rational design of high-efficiency Fischer–Tropsch synthesis catalysts capable of co-producing olefins or higher alcohols with formic acid. The by-production of formic acid can effectively enhance the atomic utilization efficiency and economic benefits of the Fischer–Tropsch synthesis process.

## 2. Computational models and methods

### 2.1 Method

#### 2.1.1 DFT method

This work employs the Vienna *Ab Initio* Simulation Package (VASP)^25–31^ with a periodic slab model, utilizing spin-polarized DFT to account for magnetic effects of the χ-Fe_5_C_2_ phase on system energetics and structure. The exchange–correlation energy is described by the Perdew–Burke–Ernzerhof (PBE) generalized gradient approximation (GGA) functional^28–30^ using the projector augmented-wave (PAW) method. A 400 eV plane-wave cutoff energy is implemented. Brillouin zone integrations for bulk and surface models employ 3 × 5 × 5 and 2 × 2 × 1 *k*-point meshes,^32^ respectively. The electron occupancies determined by the Methfessel–Paxton method (smearing width = 0.2 eV).^33^ van der Waals interactions are corrected *via* the Becke–Johnson (BJ) damped DFT-D3 method.^34,35^

Transition states are located *via* the climbing image nudged elastic band (CI-NEB) method,^36,37^ with DFT convergence criteria set to 1.0 × 10^−6^ eV for electronic self-consistent field (SCF), 0.03 eV Å^−1^ for geometry optimization, and 0.05 eV Å^−1^ for transition state searches. Converged transition states undergo vibrational frequency analysis requiring exactly one imaginary frequency for validation. The detailed calculation formulas for data calculation could be found in SI.^38–40^

#### 2.1.2 kMC method

The kinetic Monte Carlo methods, KMC could simulate chemical and physical processes taking place at crystal surfaces and is developed at Eindhoven Technical University.^41–43^ The kMC method can quantitatively characterize the specific evolution pathways of surface species on catalysts based on reaction frequency data and has been used in thermocatalysis and photocatalysis.^44,45^ This work employs a lattice-gas kMC model with the variable step size method (VSSM). The intermediate species and processes including adsorption, dissociation, migration, and reactions would be determined by the DFT data, the detailed information and calculation formulas could be found in SI.

### 2.2 Model

#### 2.2.1 DFT model

**Fig. 1:**
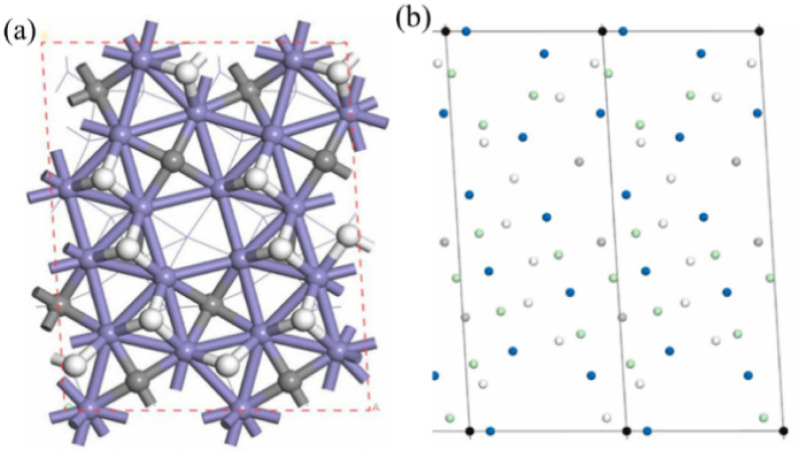
(a) The χ-Fe_5_C_2_(510) model used in DFT simulation (blue: Fe; gray: C; white: H); (b) kMC model (blue: Fe-top sites; green: Fe-bridge sites; white: Fe-hollow sites; gray: 4-fold sites).

Fig. 1(a) displays the top view of χ-Fe_5_C_2_(510), whose optimized lattice parameters (*a* = 11.570 Å, *b* = 4.501 Å, *c* = 4.986 Å, *β* = 97.56°) closely match experimental values (*a* = 11.588 Å, *b* = 4.579 Å, *c* = 5.059 Å, *β* = 97.95°), validating model reliability. The χ-Fe_5_C_2_(510) model was constructed with one H-atom layer, two Fe-atom layers and four C-atom layers according to the hydrogen coverage effect.^46,47^ The bottom Fe layer and two C layers were fixed, while other atoms and adsorbates were allowed to relax within a 13 Å vacuum layer. All geometric optimizations and transition state searches for surface adsorbates were performed on a *p*(2 × 1) supercell.

#### 2.2.2 kMC model

This work employs a lattice-gas mode^48,49^ for kMC simulations, utilizing a 64 × 64 two-dimensional periodic grid where each unit represents the *p*(1 × 1) surface of χ-Fe_5_C_2_(510). The grid contains 32 adsorption sites: 10 Fe-top sites (one-to-one correspondence with surface Fe atoms in DFT model), 10 Fe-hollow sites, 9 Fe-bridge sites, and 3 4-fold sites (including 2 C-top sites and 1 vacancy). Fig. 1(b) illustrates the kMC model.

## 3. Results and discussion

### 3.1 Construction of χ-Fe_5_C_2_(510) models with CO and H co-adsorption

**Fig. 2:**
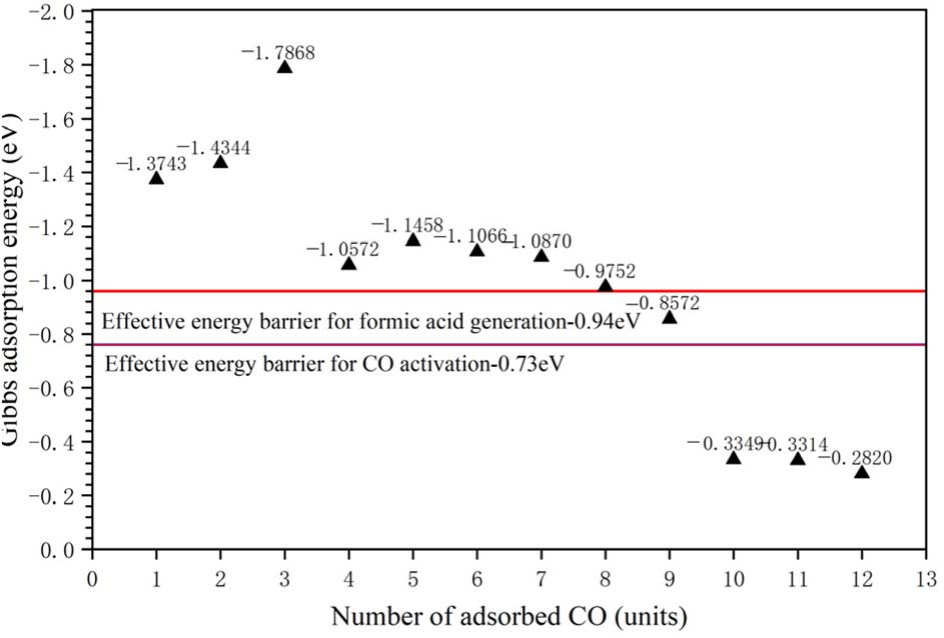
Relationship between the amount of CO adsorbed on the surface of χ-Fe_5_C_2_(510) and the variation of Gibbs CO adsorption energy.

During the Fischer–Tropsch synthesis reaction, both CO and H atoms exhibit certain surface coverages on the χ-Fe_5_C_2_(510) facet. To investigate the adsorption sites and structural stability of CO under varying coverage conditions, different amounts of adsorbed CO are sequentially introduced on the hydrogen-covered χ-Fe_5_C_2_(510) model. In the sequentially introduced process, CO initially adsorbs on Fe-top sites without neighboring adsorbed species. Subsequently, CO adsorb on Fe-top sites surrounded only by co-adsorbed CO until available sites become saturated. Ultimately, CO molecules are allowed to adsorb adjacent to dissociated O atoms when no other feasible sites remain. The CO Gibbs adsorption energies are presented in Fig. 2, while the optimal adsorption configurations are presented in SI.

As illustrated in Fig. 2, three distinct stages can be identified in terms of CO adsorption sites and adsorption energies. In the first stage, when 1–3 CO molecules are adsorbed on the *p* 2 × 1 unit cell, the intermolecular distance between CO molecules is relatively large, which is 4.91 A, 6.96 A and 7.50 A, respectively. CO preferentially adsorbs at Fe atop sites, and the spacious separation among adsorbed CO molecules renders their steric interactions negligible. In this adsorption mode, the Gibbs CO adsorption energy is higher than −1.3 eV. The adsorption and reaction behaviors under this condition are consistent with those reported in our previous studies on the chain growth mechanism and formic acid formation pathway.^19^ As the CO coverage increases to 4–9 adsorbed molecules, CO remains adsorbed on Fe atop sites. Nevertheless, the continuous increase in CO loading forces newly added CO to occupy the adjacent sites of pre-adsorbed CO, giving rise to obvious steric hindrance. This effect is reflected by the elevation of Gibbs adsorption energy, which stabilizes at approximately −1.00 eV in this stage 2. When the amount of adsorbed CO exceeds 10, its adsorption energy falls abruptly to approximately −0.3 eV. Even though CO remains adsorbed at Fe-top sites as in stage 2, intense interactions between numerous CO and surface H atoms lead to such low adsorption energy. When the number of adsorbed CO reaches 12, all available Fe-top sites become fully saturated. Further CO loading triggers the third-adsorption stage where CO molecules adsorb near dissociated O atoms. Nevertheless, the adsorption energy has already become fairly low once the CO count exceeds 10; therefore, we do not consider additional CO adsorption configurations. To further analyze why adsorption properties change with increasing CO loading, Bader-charge is carried out in Table 1.

**Table 1:** Bader-charge analysis of CO species under three different adsorption stages^a^

	1CO	9CO	12CO
C	−0.849	−0.817	−0.730
O	+0.989	+0.970	+0.965
Fe	−0.598	−0.621	−0.700

a “+” Gain charge; “−” lose charge.

As shown in Table 1, the amount of electrons lost by C atoms gradually decreases with increasing CO coverage, indicating a weakened interfacial bonding strength between CO and the χ-Fe_5_C_2_(510) model.

As the effective reaction barrier energy for CO activation and formic acid formation on the χ-Fe_5_C_2_(510) model are 0.94 eV and 0.73 eV, respectively. This indicates that the CO adsorption energy must be higher than 0.94 eV to prevent CO desorption during the reaction. Accordingly, effective CO adsorption on the χ-Fe_5_C_2_(510) model is restricted to the first and second adsorption stages.

**Fig. 3:**
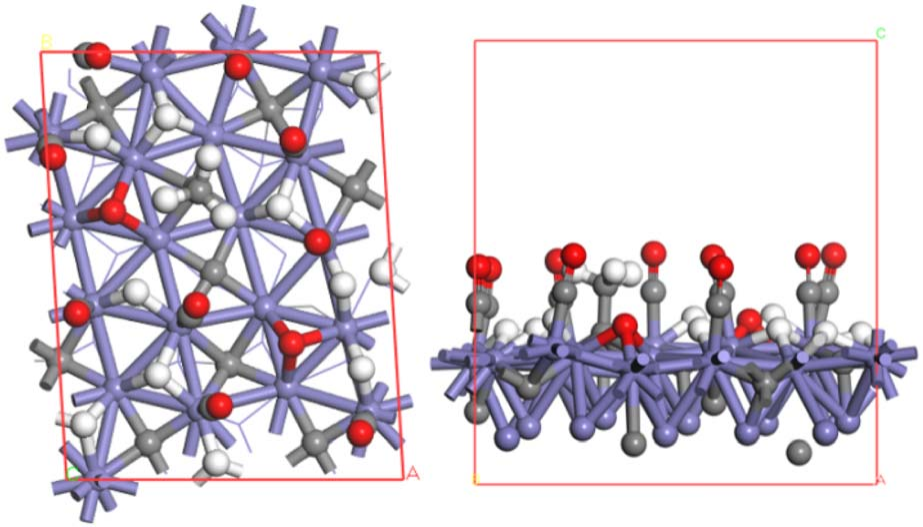
Top and left views of CO/H co-adsorbed χ-Fe_5_C_2_(510) model.

Previous studies have verified that the CO adsorption and reaction characteristics in the first stage cannot efficiently facilitate formic acid synthesis. To further explore the influence of CO coverage on the formic acid formation pathway, all subsequent investigations in this work are performed within the second CO adsorption stage. Though all CO species with adsorbed amounts between 4 and 9 show nearly identical performance as long as they belong to the second stage regime, the reactant CO would tend to migrate among different surface sites during transition-state searching, rather than undergoing the designated elementary reaction if the second-stage adsorption sites were not fully saturated by CO molecules. Hence, the constructed CO/H co-adsorbed χ-Fe_5_C_2_(510) model with nine CO molecules was ultimately selected for subsequent calculations. The top and left views of the constructed CO/H co-adsorbed χ-Fe_5_C_2_(510) model are displayed in Fig. 3. In this configuration, 9 CO molecules are anchored on the surface to eliminate the interference of CO migration on surface reactions, and all follow-up mechanistic analyses are carried out based on this optimized model.

### 3.2 Reaction pathways of formic acid formation on CO/H co-adsorbed χ-Fe_5_C_2_(510) model

#### 3.2.1 Reaction network design for formic acid formation

**Fig. 4:**
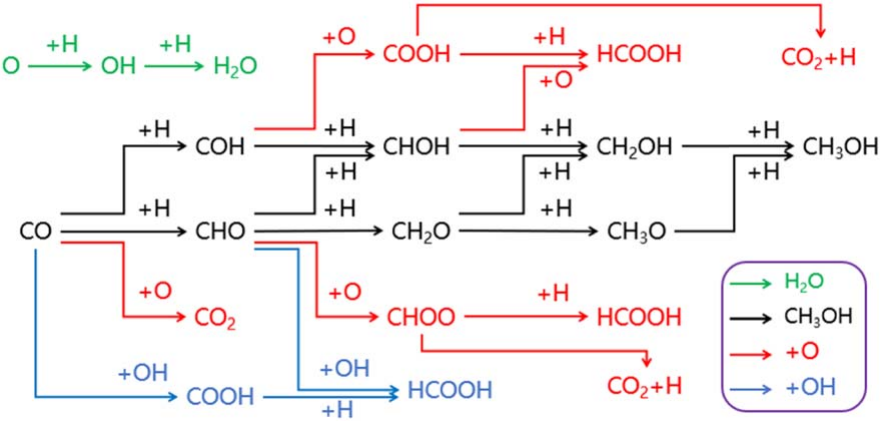
Possible dissociated O removal pathways on CO/H co-adsorbed χ-Fe_5_C_2_(510) model.

In this section, taking dissociated O, bridge-bound H, and atop CO as initial reactants, and selecting H_2_O, CO_2_, HCOOH and CH_3_OH as target products, the potential reaction network for dissociated oxygen removal is established by arranging and combining the formation sequences of C–H, C–O and O–H bonds. The corresponding reaction diagram is presented in Fig. 4. For example, CO can react with H atoms to generate COH and CHO species shown in the black pathway. Meanwhile, CO reacts with O atoms and OH species to produce CO_2_ species in the red pathway and COOH species in the blue pathway, respectively. All reaction pathways are classified into three categories for systematic discussion: the CO_2_ pathway (Pathways 1 and 2), the OH pathway (Pathways 3 and 4), and the CO pathway (Pathways 5, 6, 7 and 8). The detailed elementary reactions involved in each route are summarized in Table S1.

#### 3.2.2 Energy barrier diagrams of formic acid formation on CO/H co-covered χ-Fe_5_C_2_(510)

**Fig. 5:**
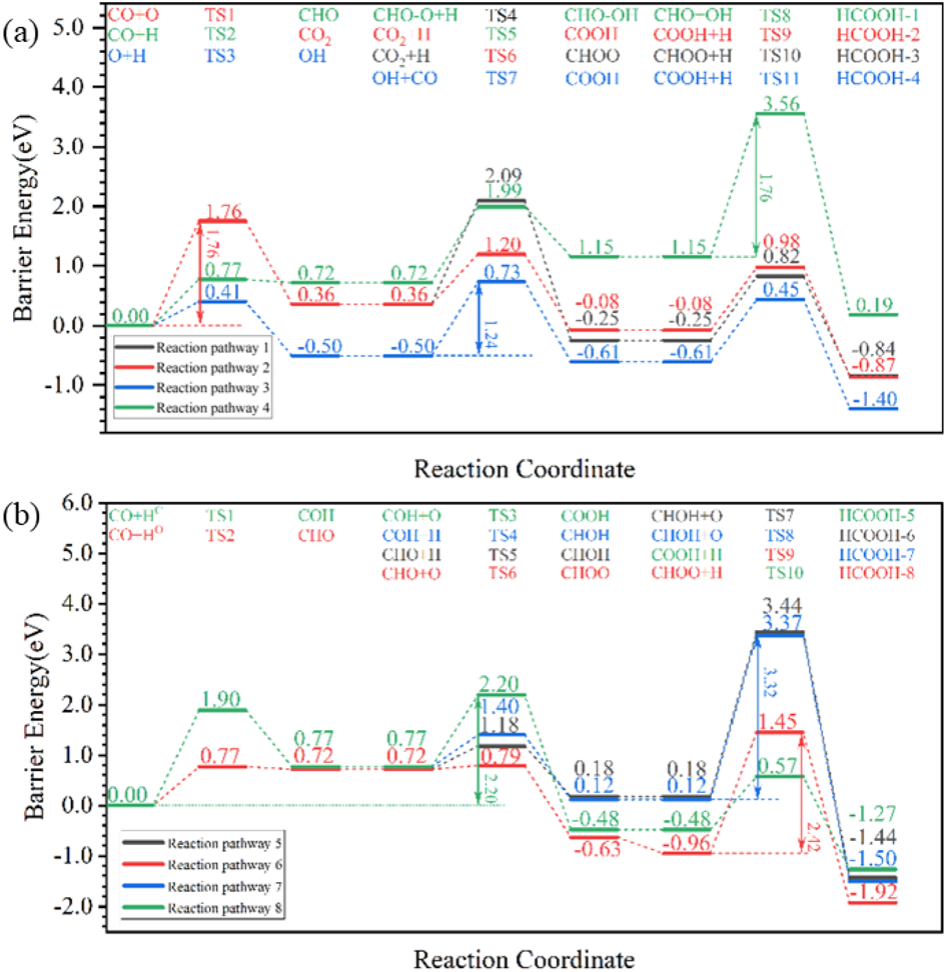
Energy barrier diagrams for dissociated oxygen removal in the form of HCOOH on CO/H co-adsorbed χ-Fe_5_C_2_(510) model: (a) CO_2_ and OH pathways; (b) CO pathway.

Fig. 5(a) and (b) present the effective reaction energy barriers for formic acid formation *via* the CO_2_/OH pathways and CO pathways of dissociated O removal, respectively. The detail configure of initial state (IS), translate state (TS) and final state (FS) for each elementary reaction could be found in Fig. S1.

In Fig. 5(a), Pathway 3 (blue line) exhibits the lowest effective barrier energy for each elementary step, which is identified as the optimal route for HCOOH production through the CO_2_/OH pathways under CO and H co-adsorption model. The rate-determining step of this pathway is the reaction CO + OH → COOH, with an effective energy barrier of 1.24 eV, which governs the overall barrier of the entire route. By contrast, all reaction routes in Fig. 5(b) possess relatively high effective barrier energy. For Pathway 5 (black line) and Pathway 7 (blue line), the elementary reaction CHOH + O → HCOOH exhibits an extremely high barrier of 3.32 eV, which dominates the overall reaction difficulty. The rate-limiting step of Pathway 6 (red line) is CHOO + H → HCOOH, with a barrier up to 2.42 eV. Among all CO pathways, Pathway 8 (green line) shows the minimum effective barrier of 2.20 eV, which is still considerably higher than that of Pathway 3 in Fig. 5(a). In summary, the most favorable reaction pathway on CO/H co-adsorbed χ-Fe_5_C_2_(510) model follows the sequence: O + H → OH + CO → COOH + H → HCOOH. Correspondingly, the overall effective energy barrier for formic acid generation is determined to be 1.24 eV.

#### 3.2.3 Formation pathway of other byproducts on CO/H co-adsorbed χ-Fe_5_C_2_(510) model

**Fig. 6:**
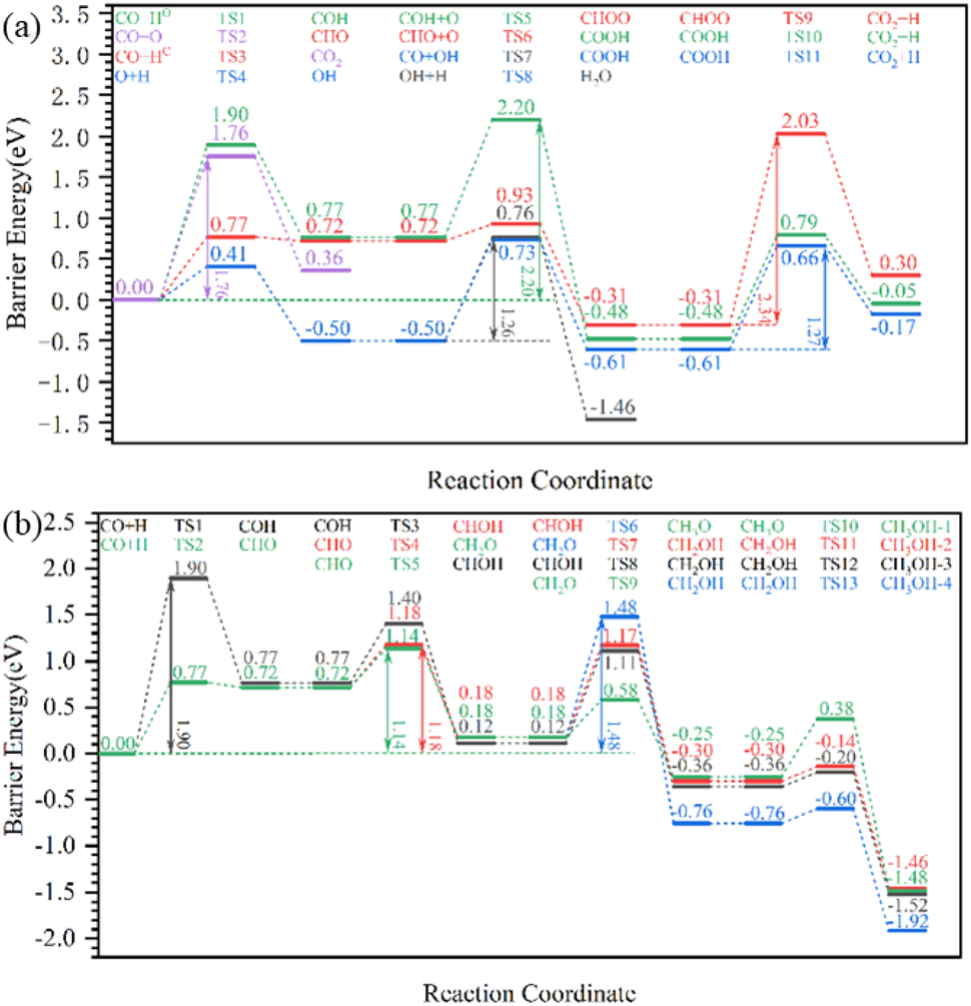
Energy barrier diagrams for dissociated oxygen removal in the form of other byproducts on CO/H co-adsorbed χ-Fe_5_C_2_(510) model: (a) H_2_O and CO_2_; (b) CH_3_OH.

CO_2_, H_2_O and methanol are the major competitive byproducts during formic acid formation. Fig. 6(a) and (b) illustrates the effective reaction barrier energy for the formation of CO_2_/H_2_O and methanol on CO/H co-adsorbed χ-Fe_5_C_2_(510) model, respectively. In Fig. 6(a), the purple, red, green, and blue lines correspond to different reaction routes for CO_2_ production, while the black line represents the two-step hydrogenation pathway of H_2_O generation. In Fig. 6(b), the black, green, blue, and red curves denote the methanol formation routes *via* stepwise CO hydrogenation. These pathways originate from the O-site hydrogenation of key intermediates, including CO, CHO, CH_2_O, and CH_3_O, respectively, to produce OH contained species.

As revealed in Fig. 6(a), the dominant rate-determining step for H_2_O formation is the elementary reaction OH + H → H_2_O, with an effective reaction barrier energy of 1.26 eV. The primary pathway for CO_2_ production follows the dehydrogenation of the COOH intermediate, expressed as O + H → OH + CO → COOH → CO_2_ + H, which exhibits an overall effective barrier energy of 1.27 eV. The effective barrier energy for the formation of these two by-products is marginally higher than that of formic acid synthesis (1.24 eV).

As shown in Fig. 6(b), the initial C-site hydrogenation of CO during methanol formation, namely CO + H → CHO, possesses a low reaction barrier of only 0.77 eV. In contrast, the O-site hydrogenation pathway CO + H → COH shows a considerably higher barrier of 1.90 eV. This preferential hydrogenation at the C-site is also observed for subsequent CH_*x*_O intermediates, indicating that stepwise hydrogenation preferentially occurs on carbon sites prior to oxygen-site. Accordingly, the major methanol formation route is identified as: CO + H → CHO + H → CH_2_O + H → CH_3_O + H → CH_3_OH.The effective barrier energy of the individual elementary reactions along this pathway are 0.77 eV, 0.42 eV, 0.41 eV and 0.63 eV, respectively, with the overall rate-limiting barrier energy determined to be 1.14 eV, which is slightly lower than that of the formic acid formation pathway.

Formaldehyde is produced *via* two successive C-site hydrogenation steps of CO, corresponding to the first two elementary reactions along the green pathway in Fig. 6(b). Although the effective barrier energy for formaldehyde generation is also 1.14 eV, its desorption energy reaches 1.31 eV, which is much higher than the barrier energy of the competing parallel reaction CH_2_O + H → CH_3_O (0.41 eV). Consequently, the direct formation and desorption of formaldehyde are thermodynamically and kinetically unfavourable.

#### 3.2.4 Competitive reaction pathways of various products on CO/H co-adsorbed χ-Fe_5_C_2_(510) model

The optimal formation pathways of various products including HCOOH, H_2_O, CO_2_ and CH_3_OH on CO/H co-adsorbed χ-Fe_5_C_2_(510) model have been clarified in the above sections. On this basis, the competitive formation relationships among different products and the influence of CO coverage are further discussed.

**Fig. 7:**
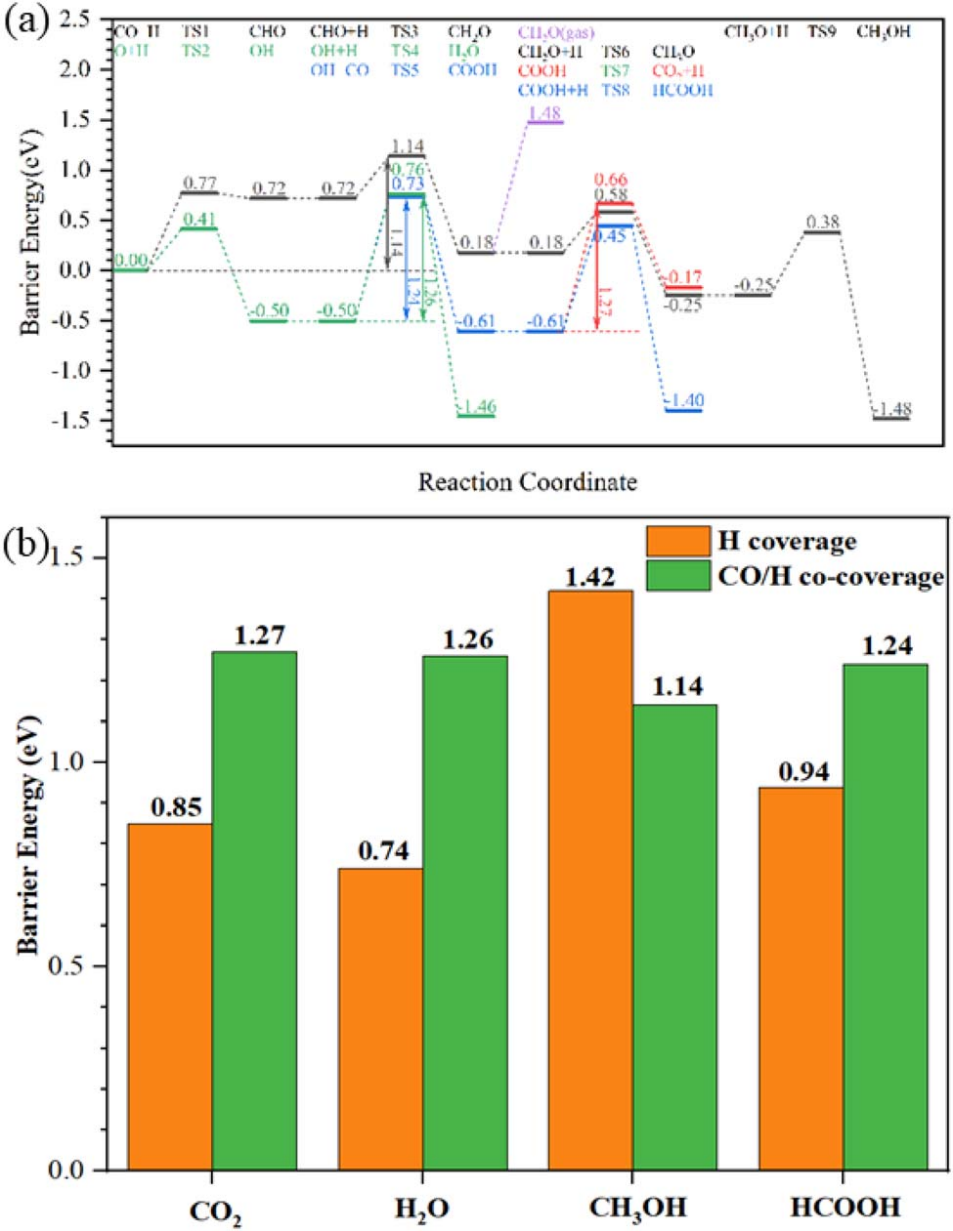
(a) Energy profile diagrams of the dominant pathways for each product on CO/H co-adsorbed χ-Fe_5_C_2_(510) model. (b) Comparison of effective barrier energy of the dominant pathways for each product under different CO coverage degree.

Fig. 7(a) compares the effective reaction barrier energy of the dominant pathways for each product. From the perspective of effective reaction barrier energy, the order is: CH_3_OH (1.14 eV) < HCOOH (1.24 eV) < H_2_O (1.26 eV) < CO_2_ (1.27 eV) < CH_2_O (1.48 eV). The differences in effective barrier reaction are relatively small. However, both CO_2_ and HCOOH formation proceed *via* the key intermediate COOH, leading to competitive reactions between COOH → CO_2_ + H (1.27 eV) and COOH + H → HCOOH (1.05 eV). Hence, the pathway toward formic acid formation is thermodynamically more favourable. Accordingly, methanol, formic acid and water can be readily produced on the CO/H co-covered χ-Fe_5_C_2_(510) surface, while carbon dioxide is difficult to form.

Fig. 7(b) compares the effective reaction barrier energy for the removal of dissociated oxygen in the forms of CH_3_OH, HCOOH, H_2_O and CO_2_ on the H-covered model and the CO/H co-covered χ-Fe_5_C_2_(510) model, respectively. As indicated in Fig. 7(b), increasing CO coverage markedly elevates the energy barriers for dissociated oxygen elimination *via* H_2_O and CO_2_ pathways, which rise from 0.74 eV to 1.26 eV and from 0.85 eV to 1.27 eV, respectively. In contrast, the barrier for formic acid formation increases moderately from 0.94 eV to 1.24 eV. These results demonstrate that enhanced CO coverage can effectively suppress the production of H_2_O and CO_2_, thereby facilitating HCOOH generation.

Nevertheless, the barrier energy for methanol formation decreases from 1.41 eV to 1.14 eV with the increase of CO coverage, which is slightly lower than that for formic acid synthesis. This suggests that adsorbed CO tends to undergo side reactions to form methanol during dissociated oxygen removal. To further clarify the detailed influence of CO coverage on formic acid production, kinetic Monte Carlo (kMC) simulations are performed based on the elementary reaction parameters derived from DFT calculations, to quantitatively reveal the intrinsic correlation between CO coverage and HCOOH formation.

### 3.3 Microkinetic investigation on HCOOH generation on CO/H co-adsorbed χ-Fe_5_C_2_(510) model

In this section, kMC simulations are performed on the χ-Fe_5_C_2_(510) facet with co-adsorbed CO and H. The microscopic kinetic parameters of the elementary reactions adopted in the simulations are listed in Table 2, and the corresponding initial state, translate state and final state configures for each elementary reaction are summarized in Fig. S2. The kMC simulation conditions are set as follows: a reaction temperature of 600 K, a reaction pressure of 2 MPa, and a simulation time of 1.0 × 10^−2^ s. After the kMC system reaches a steady state, the product distribution and reaction frequency are statistically extracted for subsequent analysis. In the kMC simulation, the dissociation O was set as the starting point rather than the CO and the CO activation is not considered This simplification of the Fischer–Tropsch synthesis (FTS) reaction pathway allows the article to exclusively focus on the oxygen removal mechanism.

**Table 2:** The elementary reactions and corresponding kinetic parameters included in the kMC simulation about the Fischer–Tropsch reaction pathway on CO/H co-covered χ-Fe_5_C_2_(510) model^a^

Elementary reaction	*A*_for_	Δ*G*_a,f_ (kcal mol^−1^)	*A*_des_	Δ*G*_a,r_ (kcal mol^−1^)
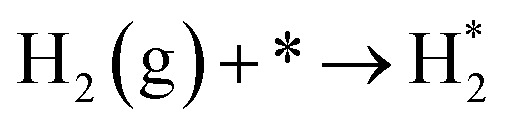	1.18 × 10^7^	—	1.00 × 10^13^	5.53
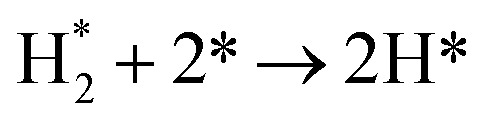	9.89 × 10^12^	0.69	1.88 × 10^13^	3.23
H* + * → * + H*	1.28 × 10^13^	3.92	1.28 × 10^13^	—
CO(g) + * → CO*	3.18 × 10^6^	—	1.00 × 10^13^	32.98
CO* + * → * + CO*	2.77 × 10^13^	4.38	2.77 × 10^13^	4.38
CHOO-1 → CHOO	4.69 × 10^12^	35.95	4.59 × 10^12^	28.50
CHO + OH → HCOOH	1.27 × 10^13^	77.77	1.98 × 10^13^	55.51
CHOO + H → HCOOH	1.90 × 10^12^	38.36	1.03 × 10^12^	24.67
CO_2_ + H → CHOO	2.43 × 10^13^	53.93	1.66 × 10^13^	39.97
HCOH + O → HCOOH	3.49 × 10^14^	112.41	1.19 × 10^11^	75.13
COH + H → HCOH	7.39 × 10^13^	29.65	6.49 × 10^13^	14.68
COOH + H → HCOOH	2.74 × 10^13^	42.51	3.83 × 10^12^	24.31
CO + H → CHO	2.13 × 10^12^	1.24	8.37 × 10^12^	17.78
CO_2_ + H → COOH	9.97 × 10^12^	29.34	6.46 × 10^12^	19.32
HCO-1 → CHO	3.20 × 10^13^	26.97	1.00 × 10^13^	37.09
CHO + O → CHOO	2.58 × 10^12^	41.10	9.30 × 10^12^	1.75
O + H → OH	1.23 × 10^13^	21.04	4.03 × 10^12^	9.41
CHO + O + H → CHO + OH	2.24 × 10^13^	19.40	1.64 × 10^13^	29.46
CO + OH → COOH	4.39 × 10^12^	31.72	3.40 × 10^12^	28.51
CO + O → CO_2_	7.32 × 10^12^	32.21	3.29 × 10^12^	40.48
CO + H → COH	1.86 × 10^13^	26.06	4.45 × 10^13^	43.71
HCO + H → HCOH	1.93 × 10^12^	23.14	4.77 × 10^12^	10.71
OH + H → H_2_O	6.15 × 10^12^	50.98	2.48 × 10^11^	29.13
H_2_CO + H → H_2_COH	1.51 × 10^13^	51.52	1.60 × 10^13^	30.00
HCO + H → H_2_CO	2.14 × 10^12^	22.22	4.06 × 10^12^	9.75
H_3_CO + H → H_3_COH	1.13 × 10^13^	42.92	2.80 × 10^13^	14.52
H_2_CO + H → H_3_CO	8.53 × 10^12^	19.28	2.35 × 10^12^	9.42
H_2_COH + H → H_3_COH	4.98 × 10^12^	30.40	1.21 × 10^12^	3.67
HCOH + H → H_2_COH-1	9.22 × 10^12^	10.21	2.04 × 10^12^	0.75
H_2_COH-1 → H_2_COH	4.32 × 10^12^	33.99	6.96 × 10^12^	32.44
COH + O → COOH	2.21 × 10^13^	61.71	1.53 × 10^13^	32.98

a *A*_for_/*A*_des_: forward/reverse pre-exponential factor; Δ*G*_a,f_/Δ*G*_a,r_: forward/reverse Gibbs barrier energy.

Microkinetic analysis results are presented in Table 3, in which the inlet molar ratios of CO to H_2_ were set to 1 : 2, 1 : 1, and 2 : 1. At a CO : H_2_ molar ratio of 1 : 2, the system remained favourable for H_2_O formation with a reaction frequency of 3180, accompanied by a small amount of methanol. As the CO proportion in the feed increased, methanol production gradually rose, while H_2_O formation declined accordingly. Formic acid began to emerge distinctly when the CO : H_2_ molar ratio was adjusted to 2 : 1.

**Table 3:** Product distributions and reaction frequencies for kMC simulations of CO and H covered χ-Fe_5_C_2_(510)surfaces with different feed ratios

H_2_ : CO	H_2_O	CO_2_	HCHO	CH_3_OH	HCOOH
2 : 1	3180	0	0	102	0
1 : 1	365	0	0	816	0
1 : 2	141	0	0	1706	1

On one hand, kMC results verify that raising CO proportion in feed gas efficiently accelerates the elimination of surface dissociated oxygen in the form of formic acid, and microkinetic results prove the feasibility of oxygen removal *via* HCOOH routes over iron carbide catalysts. On the other hand, though, DFT results reveal that the effective barrier of HCOOH formation is slightly lower than that of H_2_O and marginally higher than that of CH_3_OH, the reaction frequency of HCOOH is much lower than those of H_2_O and CH_3_OH in kMC simulations.

This difference mainly originates from the fact that HCOOH formation needs direct CO involvement, while adsorbed CO on χ-Fe_5_C_2_(510) tends to undergo successive hydrogenation with bridge H species to form methanol. Moreover, the activation of adsorbed CO is not considered in the present kMC model, which limits surface CO reactions to methanol formation and leads to its extremely high reaction frequency.

In addition, the dominant H_2_O formation pathway remains consistent with that on the hydrogen-covered model. Although elevating CO coverage can suppress H_2_O production and facilitate HCOOH generation to a certain extent, the promotional effect still requires further improvement.

## 4. Conclusions

The removal of dissociated oxygen in the form of formic acid on the χ-Fe_5_C_2_(510) surface is possible *via* OH intermediates. However, OH intermediates tend to readily react with adsorbed hydrogen to form H_2_O, which hinders formic acid synthesis. Accordingly, this work proposes increasing surface CO coverage to enhance the reaction probability between adsorbed CO and OH species, thereby promoting the conversion of dissociated oxygen into formic acid and realizing resource utilization.

In this work, the configurations and adsorption energies of CO with different coverages are systematically analysed and is found three distinct coverage stages were identified. When 1–3 CO molecules are adsorbed, the adsorption energy remains above −1.4 eV, and the corresponding adsorption and reaction behaviors are consistent with previously reported results. When 4 to 9 CO molecules are adsorbed, steric hindrance arises between adjacent CO species, causing the adsorption energy to drop to around −1.00 eV. With ten or more CO molecules adsorbed on the surface, the adsorption energy drops further to about −0.3 eV, exhibiting typical physical adsorption features. As the effective barrier energy for CO activation and formic acid formation on χ-Fe_5_C_2_(510) model are 0.94 eV and 0.73 eV, respectively, requiring CO adsorption to be maintained within the first and second coverage stages. On this basis, the surface model with nine adsorbed CO molecules in the *p* 2 × 1 unit is determined as the most reasonable CO/H co-adsorbed structure for subsequent investigation.

Based on the constructed co-adsorption model, DFT calculations demonstrate that the optimal formation pathway of formic acid proceeds *via* the reaction sequence: O + H → OH + CO → COOH + H → HCOOH, with an overall effective energy barrier of 1.24 eV. Compared with previous findings, increased CO coverage raises the energy barrier of the main HCOOH formation pathway from 0.94 eV to 1.24 eV, which moderately suppresses its reaction kinetics. Nevertheless, the energy barriers of the competitive pathways for H_2_O and CO_2_ formation rise more significantly, increasing from 0.74 eV to 1.26 eV and from 0.85 eV to 1.27 eV, respectively. Such changes greatly promote the selective conversion of dissociated oxygen through the formic acid formation route.

Subsequent kMC simulations confirm that formic acid can be synthesized on the CO/H co-covered χ-Fe_5_C_2_(510) model at a CO : H_2_ feed ratio of 2 : 1. Nevertheless, the formation of H_2_O still acts as the primary pathway for the elimination of dissociated oxygen. This phenomenon stems from the high hydrogenation activity of hydrogen atoms at bridge sites. On the one hand, bridge-site hydrogen directly facilitates the conversion of surface oxygen species into water. On the other hand, the CO reactant needed for formic acid synthesis tends to undergo stepwise hydrogenation with bridge-site H to generate methanol, thereby lowering the collision frequency between CO and OH intermediates.

The theoretical simulation results of this work lay a solid theoretical foundation and offer explicit design guidance for developing high-performance Fischer–Tropsch synthesis catalysts toward formic acid coproduction. Further optimization of active site structures and reaction conditions in follow-up studies will facilitate the efficient synthesis of high-value formic acid by-products and further enhance the economic benefits and environmental sustainability of industrial Fischer–Tropsch processes.

## Author contributions

Jinchun Jiang and Hongzhi Zheng: methodology, investigation, data curation, and conceptualization. Changyi Lai and Qining Wang: formal analysis and data curation. Jie Ren: validation, methodology. Ning Ai: software. Wei Zhou: supervision.

## Conflicts of interest

The authors declare that they have no known competing financial interests or personal relationships that could have influenced the work reported in this paper.

## Supplementary Material

RA-OLF-D6RA04509J-s001

## Data Availability

The data supporting this article have been included as part of the supplementary information (SI). The code for [VASP] can be found at [https://vasp.at/]. The version of the code employed for this study is version [6.4.3]. Supplementary information: method details and all the structures of the initial state, transition structure and final structure for each elementary reaction. See DOI: https://doi.org/10.1039/d6ra04509j.
