# Supplementary material for: Theoretical insight into the effect of CO coverage on formic acid formation from dissociated oxygen on χ-Fe5C2(510) in Fischer–Tropsch synthesis
Source: RSC Adv. 2026 Jul 23;16(38):42520–7. doi: 10.1039/d6ra04509j (PMC13392752; doi:10.1039/d6ra04509j)
Supplement: Supplementary file 1 [file RA-016-D6RA04509J-s001.pdf]

Supplementary Information (SI) for RSC Advances.
This journal is © The Royal Society of Chemistry 2026

# Theoretical Insight into the Effect of CO Coverage on Formic Acid Formation from Dissociated Oxygen on χ-$F_e5C_2$(510) in Fischer-Tropsch Synthesis

Jinchun Jiang [a], Hongzhi Zheng [a], Changyi Lai [a], qining Wang[b] Jie Ren [a], Ning Ai [a,b], Wei Zhou [c,*]

(a) College of Biological Chemical Science and Engineering, Jiaxing University,Jiaxing University, Jiaxing, China.

(b) National Demonstration Center for Experimental Chemistry and Chemical Engineering Education, Zhejiang University of Technology, Hangzhou China.

(c) Sichuan Technology and Business University, Chengdu,China;.

## I. The detailed calculation formulas for DFT method

The adsorption energies $E_{\mathrm{ads}}$ (eV) are computed using Equation 1:

$$E_{ads} = E_{(adsorbate/slab)} - [E_{(slab)} + E_{(adsorbate)}] \quad (1)$$

Here, $E_{(\mathrm{adsorbate/slab})}$(eV), $E_{(\mathrm{slab})}$(eV), and $\mathrm{E}_{(\mathrm{adsorbate})}$(eV) denote the total energy of the adsorbate-surface system energy, the clean surface energy, and the isolated adsorbate energy, respectively.

The desorption energies $E_{\mathrm{des}}$ (eV) are computed using Equation 2:

$$E_{des} = E_{(desorbate/slab)} - [E_{(slab)} + E_{(desorbate)}] \quad (2)$$

Here, $E_{(\mathrm{desorbate/slab})}$(eV), $E_{(\mathrm{slab})}$(eV), and $\mathrm{E}_{(\mathrm{desorbate})}$(eV) denote the total energy of the desorbate-surface system energy, the clean surface energy, and the isolated desorbate energy, respectively.

To evaluate standard molar Gibbs free energies (*G*), The zero-point energy (*ZPE*) corrections, standard molar vibrational internal energy contributions, and vibrational entropy effects were applied, G could be computed via Equation 3:

$$G = E + ZPE + U^0 + \gamma RT(1 + ln\, {}^{p}\!/_{p^0}) - TS^0 \quad (3)$$

*E* denotes electronic energy, *ZPE* denotes zero-point energy, $U^0$ denotes standard molar vibrational internal energy at 0 K, p denotes system pressure, $\gamma$ denotes the phase coefficient (0 for surfaces, 1 for gases), and $S^0$ standard molar vibrational entropy.

The standard molar Gibbs free energy (*G*) values are applied to calculate the Gibbs reaction energy ($\Delta G_r$ = *G*(FS) - *G*(IS)) and Gibbs barrier energy ($\Delta G_a$ = *G*(TS) - *G*(IS)), where *G*(IS), *G*(FS), and *G*(TS) denote the Gibbs free energies of the initial state, final state, and transition state, respectively.

This work addresses complex reaction network featuring extensive consecutive and parallel pathways. In consecutive steps, the coverage of intermediate species critically influences reaction rates according to law of mass action. Consequently, identifying rate-determining steps requires more than elementary

reaction energy barriers alone. We employ effective Gibbs barrier energies ($G_{a,eff}$) to account for reactant coverage effects on product formation rates[34-36], the $G_{a,eff}$ for the $k^{th}$ reaction step would be calculated via Equation 4:

$$G_{a,eff}^{k} = G_a^k + \sum_{i=j}^{k-1} G_r^i \quad (4)$$

$G^k{}_a$(eV) and $G^i{}_r$(eV) represent the Gibbs barrier energy of the $k^{th}$ step and the Gibbs reaction energy of the $i^{th}$ step, respectively. The $j^{th}$ step reaction represented by j is the elementary step with the lowest product energy in a series of reactions.

This work defines the transitions of adsorbate between different stable adsorption states as migration reactions, which may involve one or multiple elementary reactions—each with distinct initial, transition, and final states. Computational analysis reveals minimal overall energy changes and low barriers in most migration processes, enabling rapid equilibrium attainment. Under such conditions, the Gibbs reaction energy rather than the Gibbs barrier energy is important. Hence, to prevent energy profile diagram becoming complex due to migration reactions, only the Gibbs reaction energy of migration reactions—defined as Gibbs migration energy ($G_m$)—were considered when evaluating migration effects on reaction pathways.

## II . The detailed calculation formulas for kMC method

The reactant adsorption rate $k_{ads}$ (1/s) is given by Equation 5:

$$k_{ads} = \frac{\sigma P A_{site}}{\sqrt{2\pi m k_B T}} \tag{5}$$

Here, $\sigma$ denotes the dimensionless sticking coefficient (typically set to 1), $P$(Pa) and $T$(K) represent gas partial pressure and temperature under simulated conditions, $A_{site}$($m^2$) is the per-site adsorption area, $m$(kg) is molecular mass, and $K_B$(J/K) is Boltzmann constant.

The forward rate constants $k_i$ are calculated using the Arrhenius formulation in Equation 6, where $A_i$ are the pre-exponential factor calculated via transition state theory in Equation 7**:**

$$k_i = A_i e^{-\frac{G_i}{K_B T}} \tag{6}$$

$$A_i = \frac{K_B T}{h} \frac{\prod_{i,TS} \frac{1}{1 - e^{-\frac{h v_i}{K_B T}}}}{\prod_{i,IS} \frac{1}{1 - e^{-\frac{h v_i}{K_B T}}}} \tag{7}$$

Here, $k_B$(J/K) denotes the Boltzmann constant, $T$(K) is the reaction temperature, $h$ (J·s) is Planck's constant, $vi^{TS}$($m^{-1}$) denotes the ith vibrational frequency of the transition state, and $vi^{IS}$($m^{-1}$) is the corresponding vibrational frequency of the initial state

**III. The optimal adsorption configurations of CO adsorption with different amount in Fig.2**

| 1 | 2 | 3 | 4 |
|---|---|---|---|
| 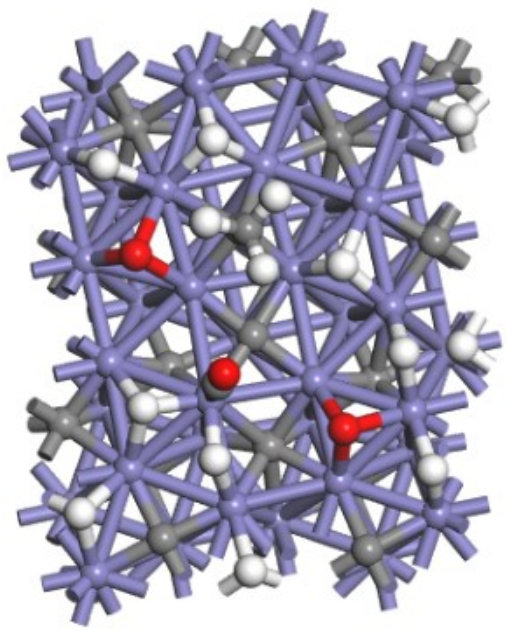 | 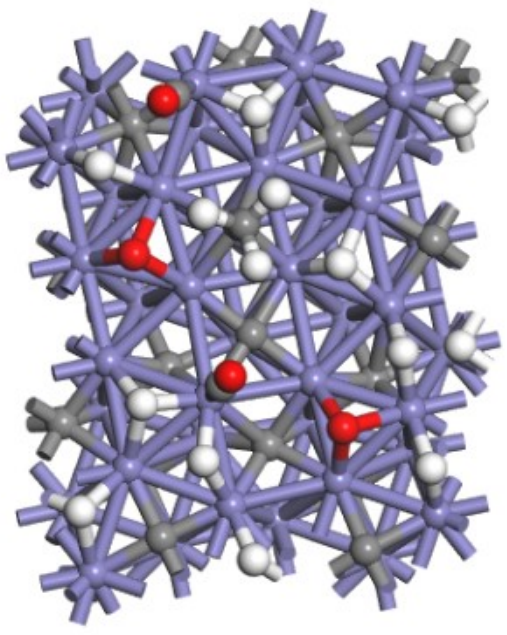 | 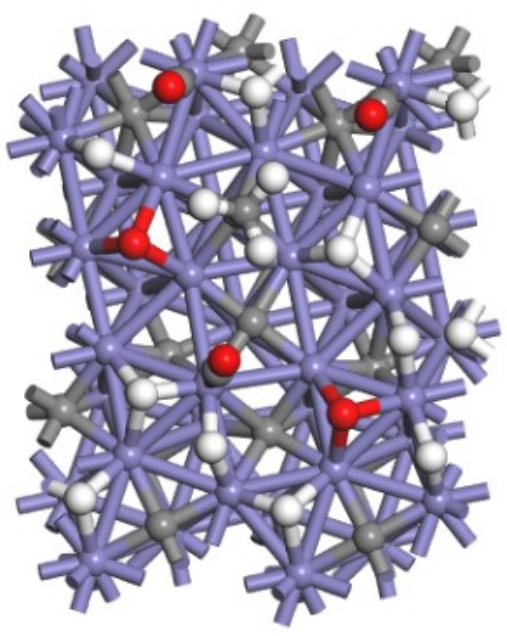 | 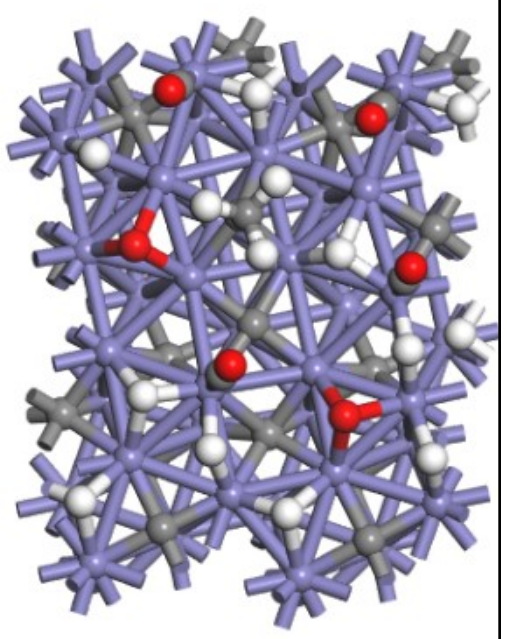 |
| 5 | 6 | 7 | 8 |
| 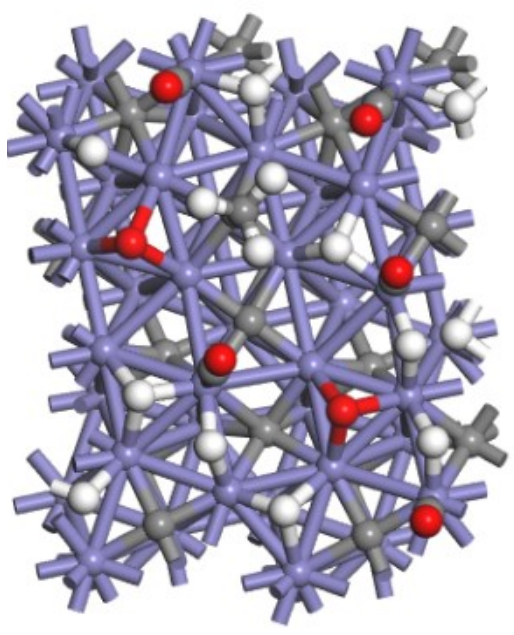 | 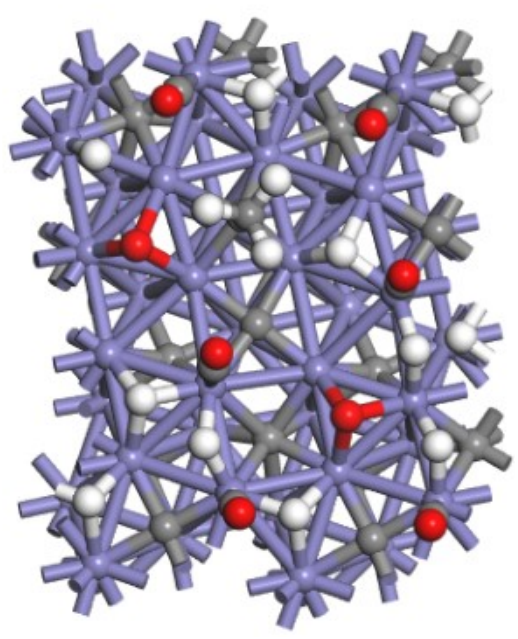 | 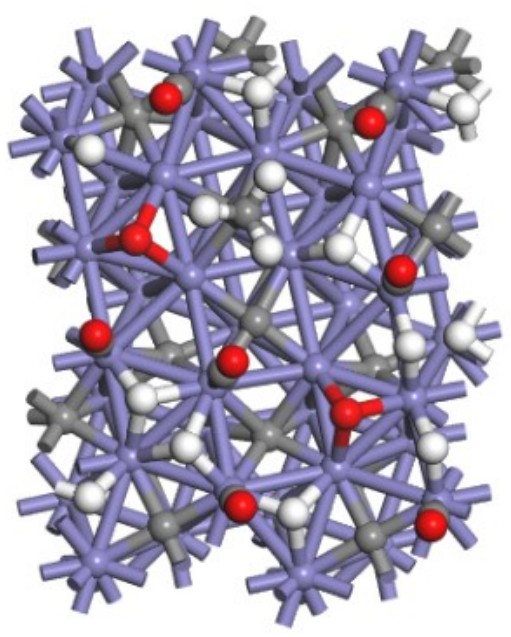 | 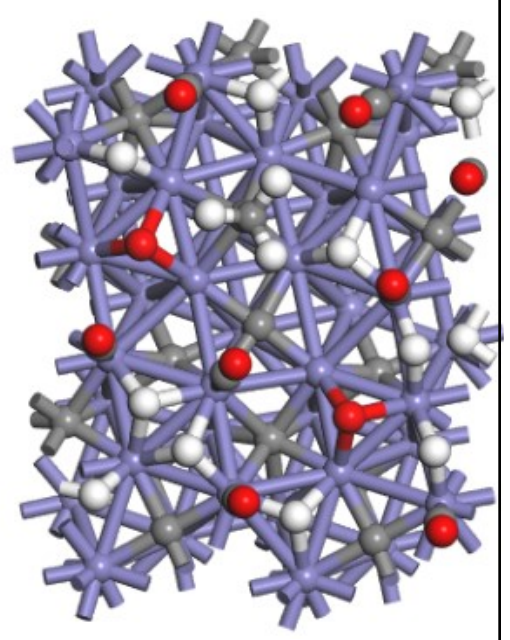 |
| 9 | 10 | 11 | 12 |

| Reaction pathway | First step | Second step | Third step |
|---|---|---|---|
| 1 | CO+O→$CO_2$ | $CO_2$+H→CHOO | CHOO+ H→HCOOH |
| 2 | CO+O→$CO_2$ | $CO_2$+H→COOH | COOH+ H→HCOOH |
| 3 | O+H→OH | OH+CO→COOH | COOH+ H→HCOOH |
| 4 | CO+H→CHO | O+H→OH | CHO+OH→HCOOH |
| 5 | CO+H→CHO | COH+H→CHOH | CHOH+O→HCOOH |
| 6 | CO+H→CHO | CHO+O→CHOO | CHOO+H→HCOOH |
| 7 | CO+H→COH | COH+H→CHOH | CHOH+O→HCOOH |
| 8 | CO+H→COH | COH+O →COOH | COOH+ H→HCOOH |

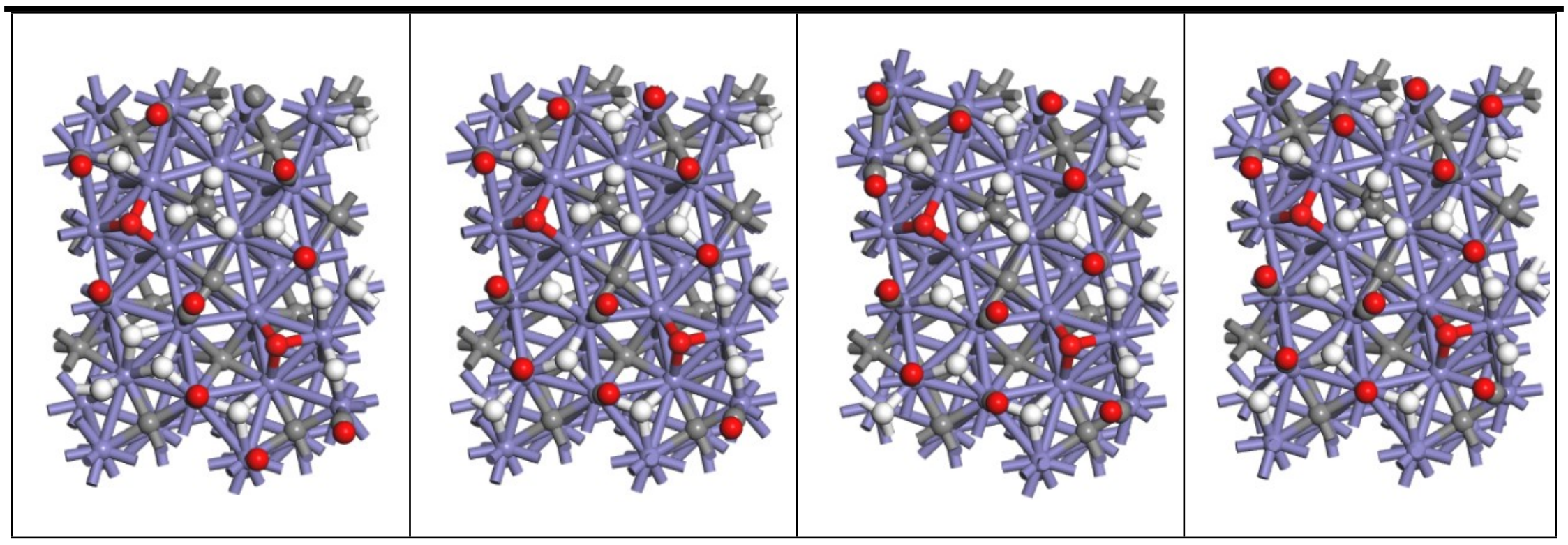

## IV. The detailed elementary reactions involved in each route diagram of Fig.4

## V. The detail configure of initial state(IS), translate state(TS) and final state(FS) for each elementary reaction in Fig5(a)

| Elementary reaction | Initial state | Translate state | Final state |
|---|---|---|---|
| TS1<br>$CO+O \rightarrow CO_2$ | 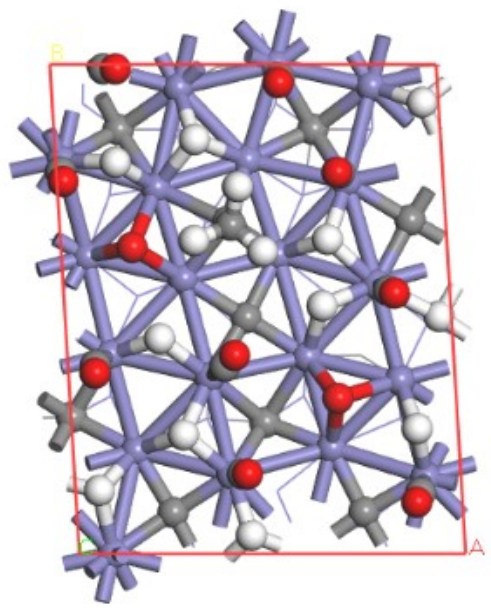 | 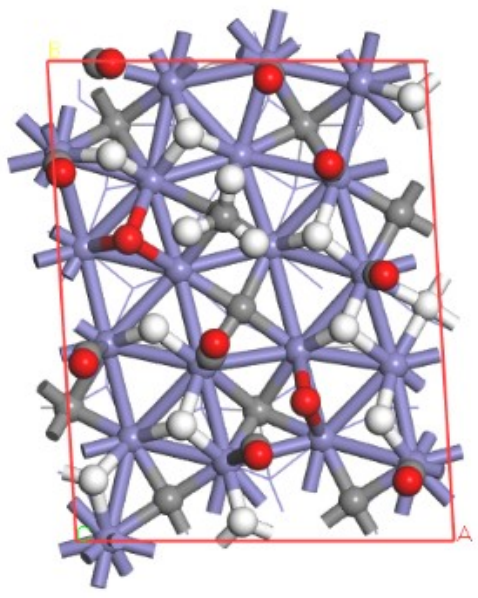 | 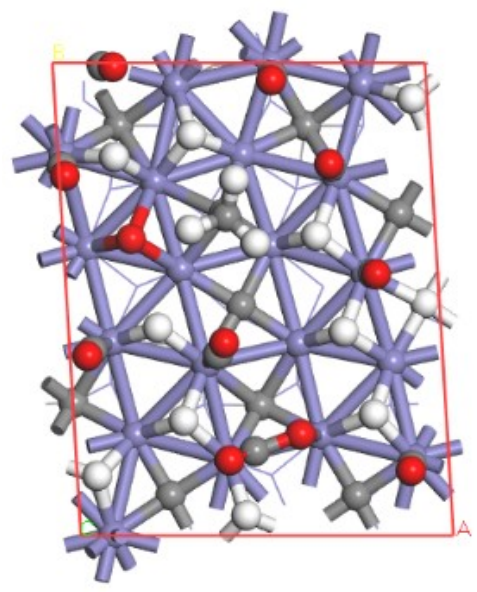 |
| TS2<br>$CO+H \rightarrow CHO$ | 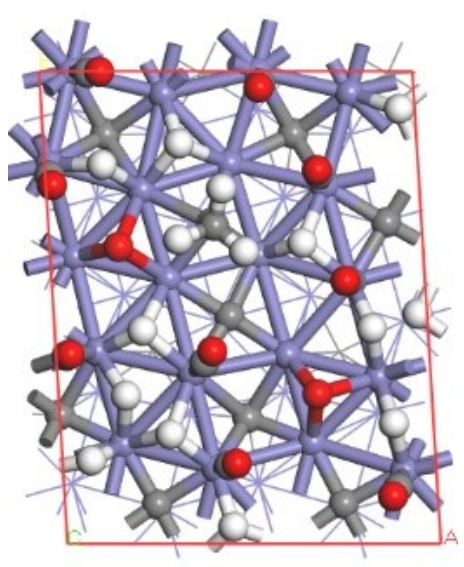 | 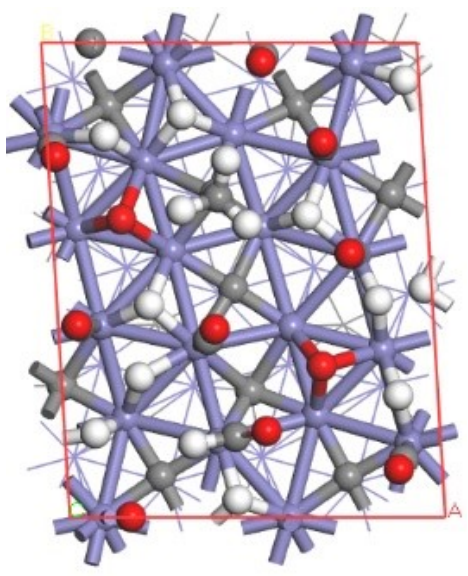 | 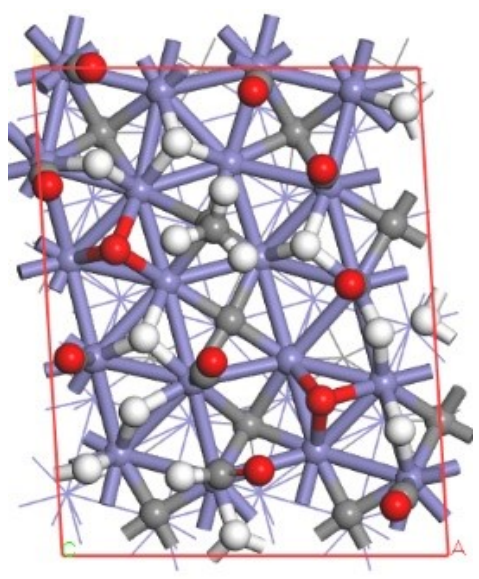 |

TS3
$O+H \rightarrow OH$

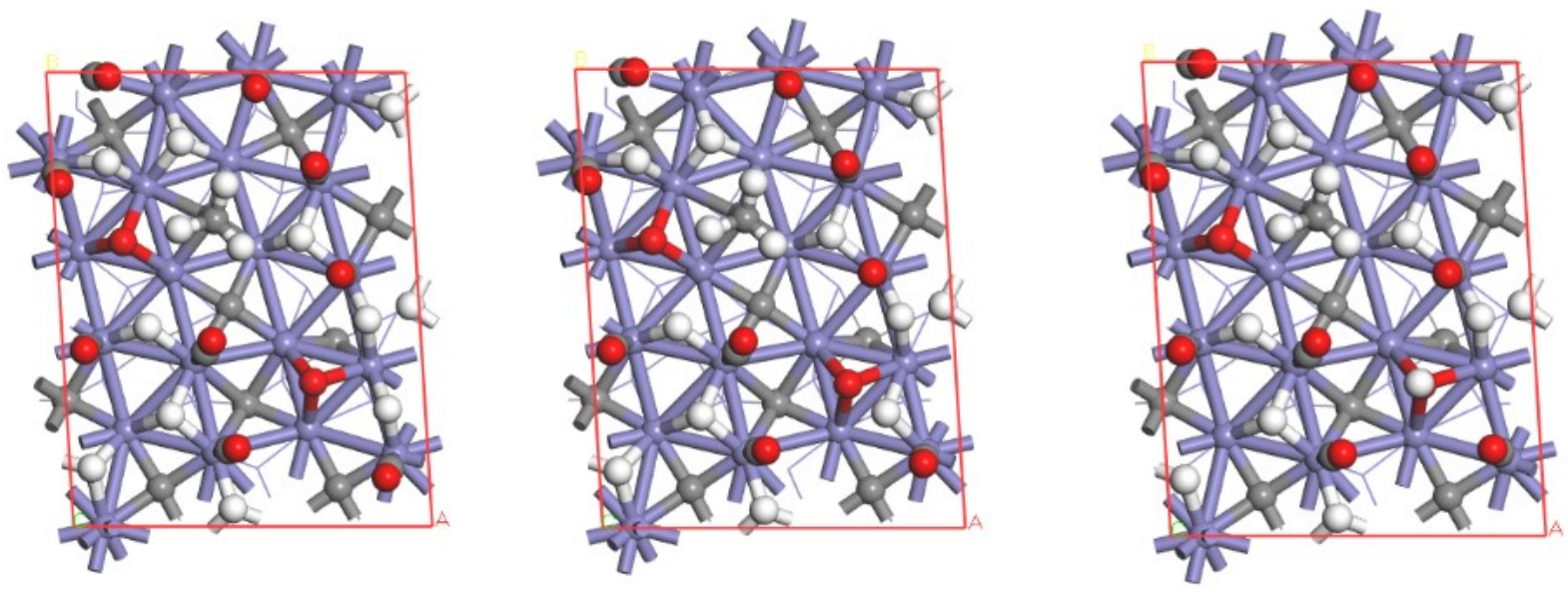

TS4
$CO_2+H \rightarrow CHOO$

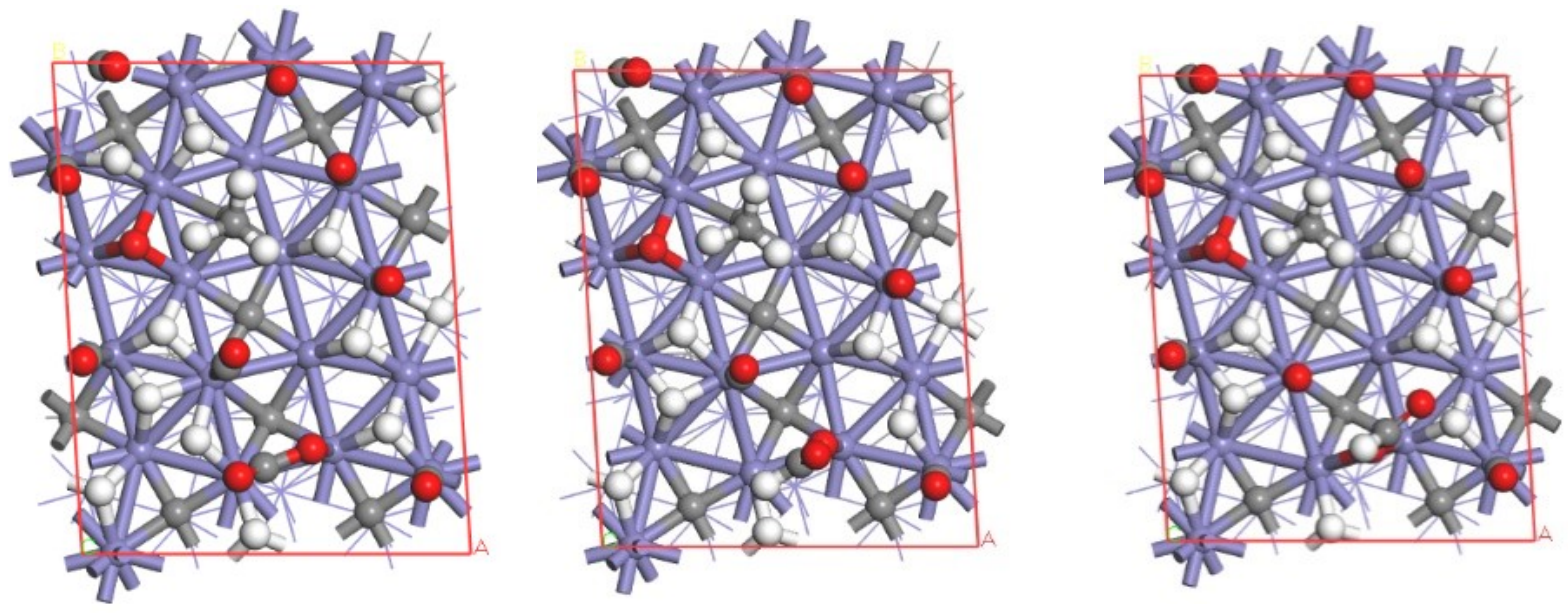

TS5
CHO_O+H→CHO_OH

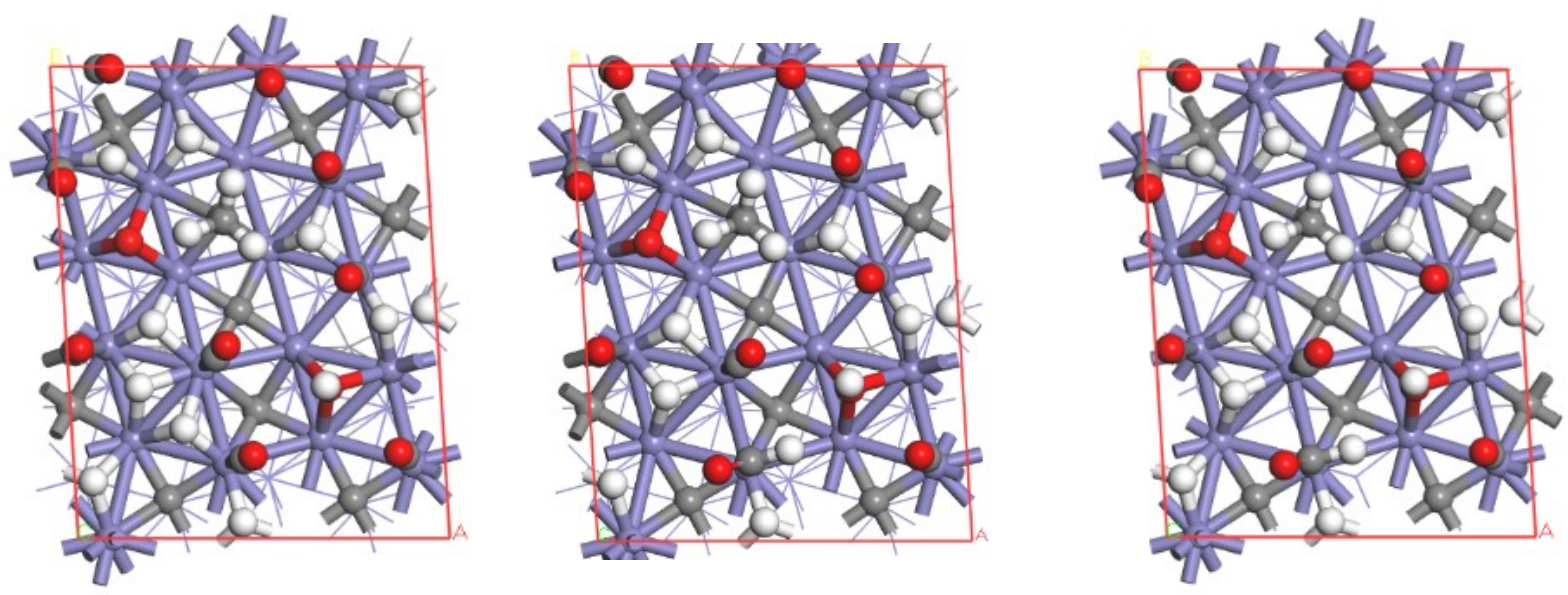

TS6
$CO_2+H \rightarrow COOH$

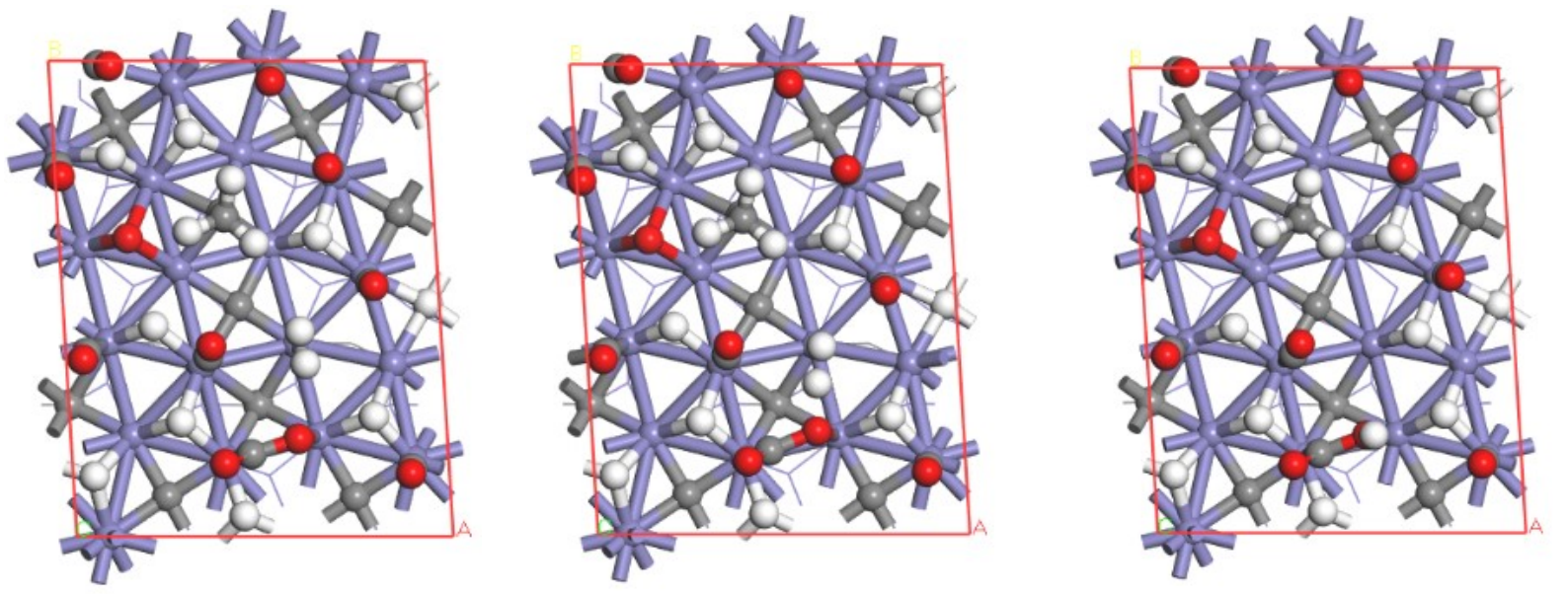

TS7
$OH+CO \rightarrow COOH$

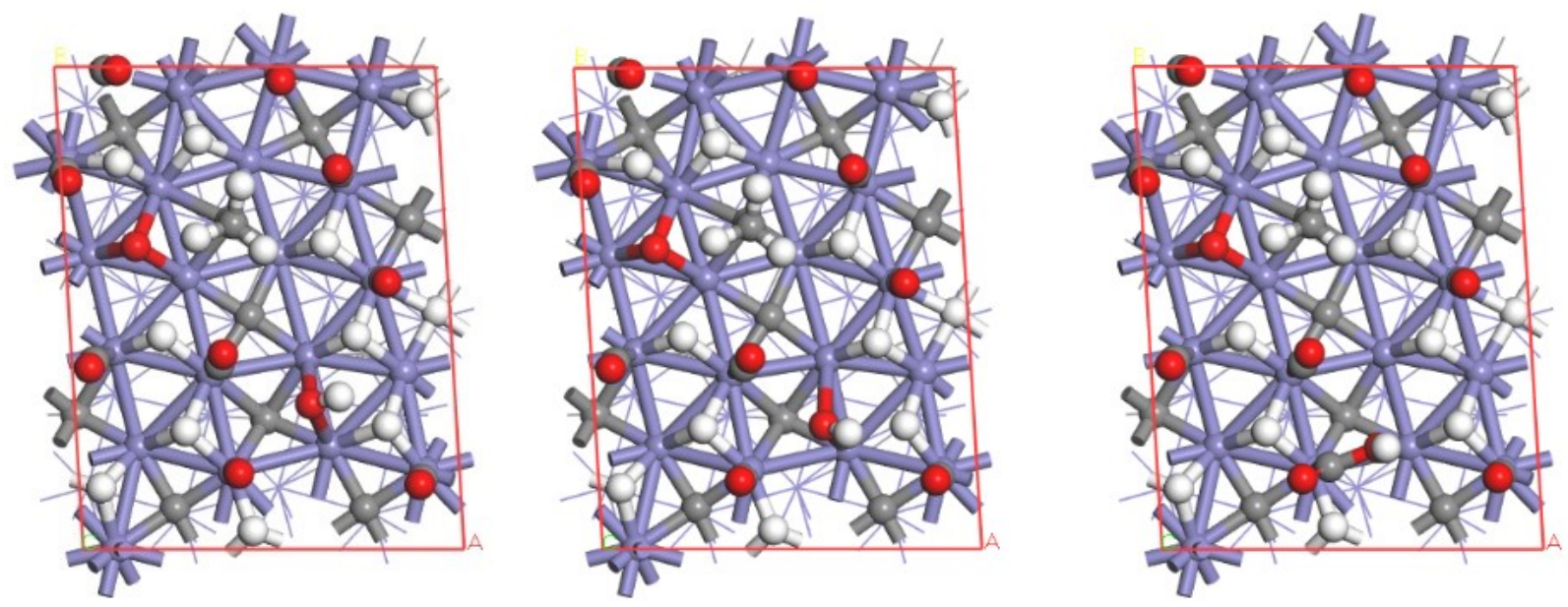

TS8
CHO+OH→HCOOH-1

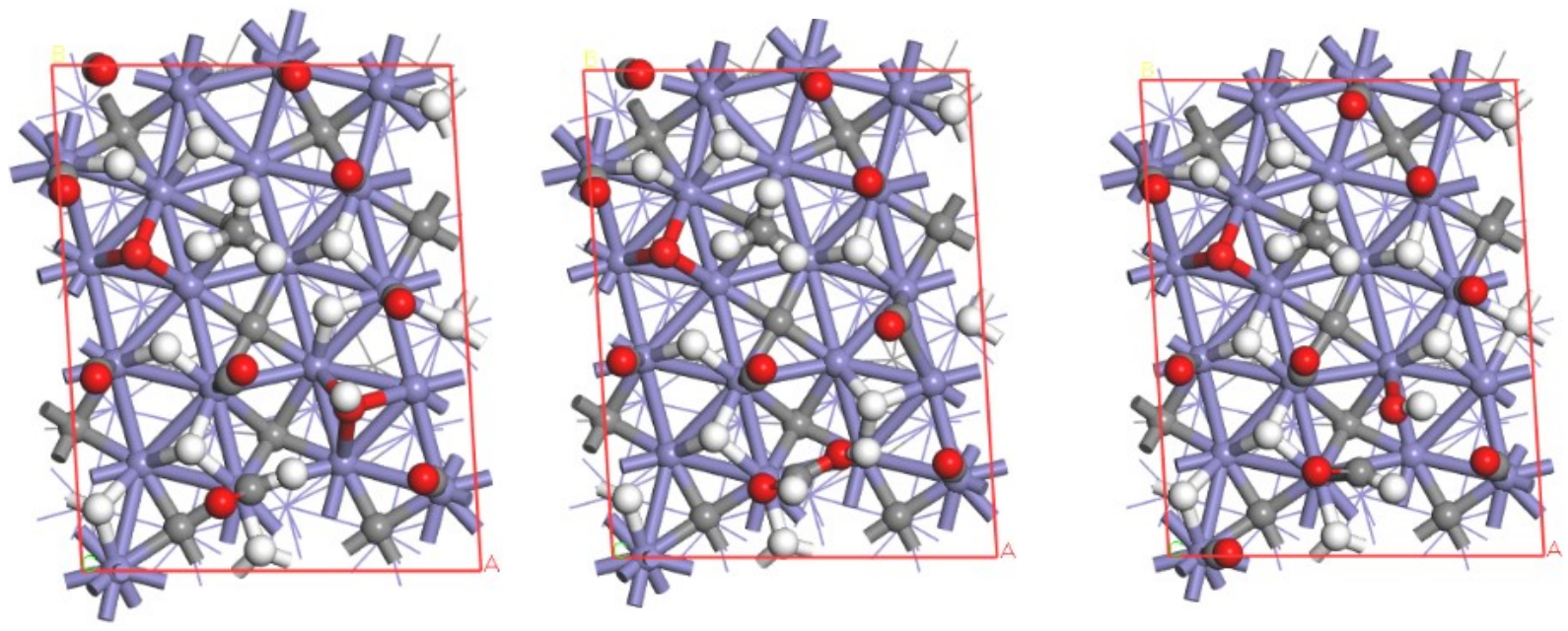

TS9
COOH+H→HCOOH-2

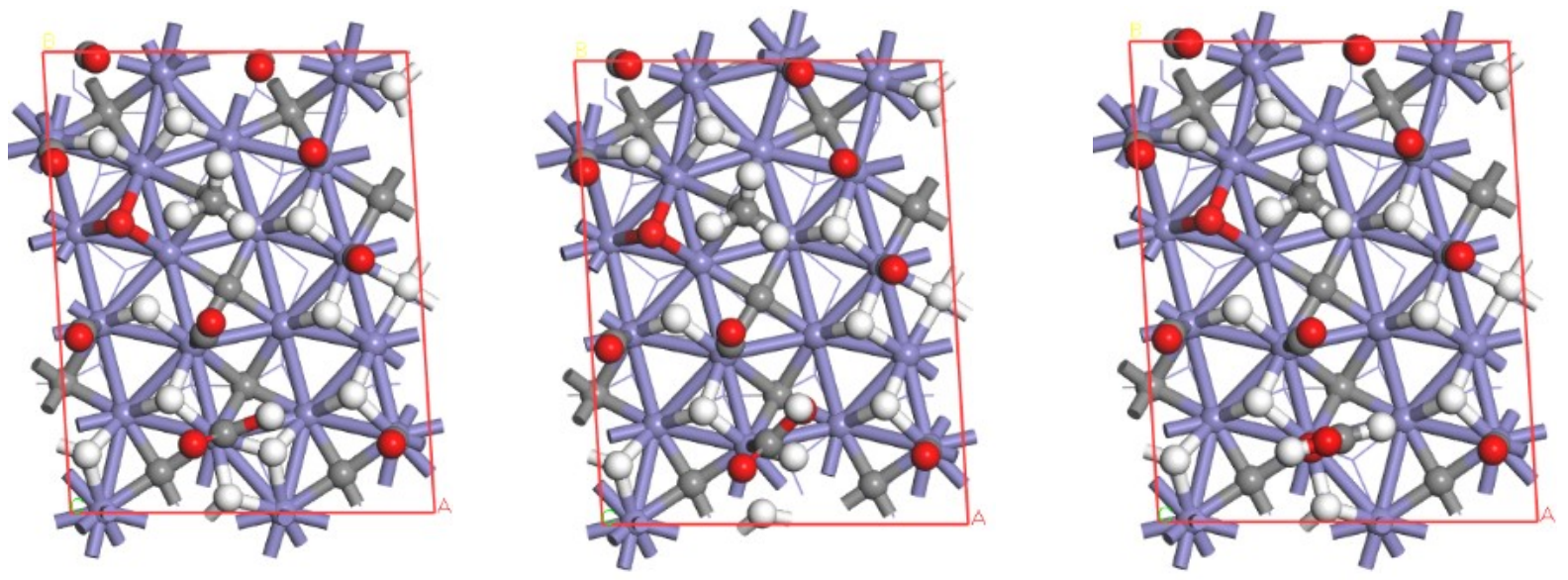

TS10
CHOO+H→HCOOH

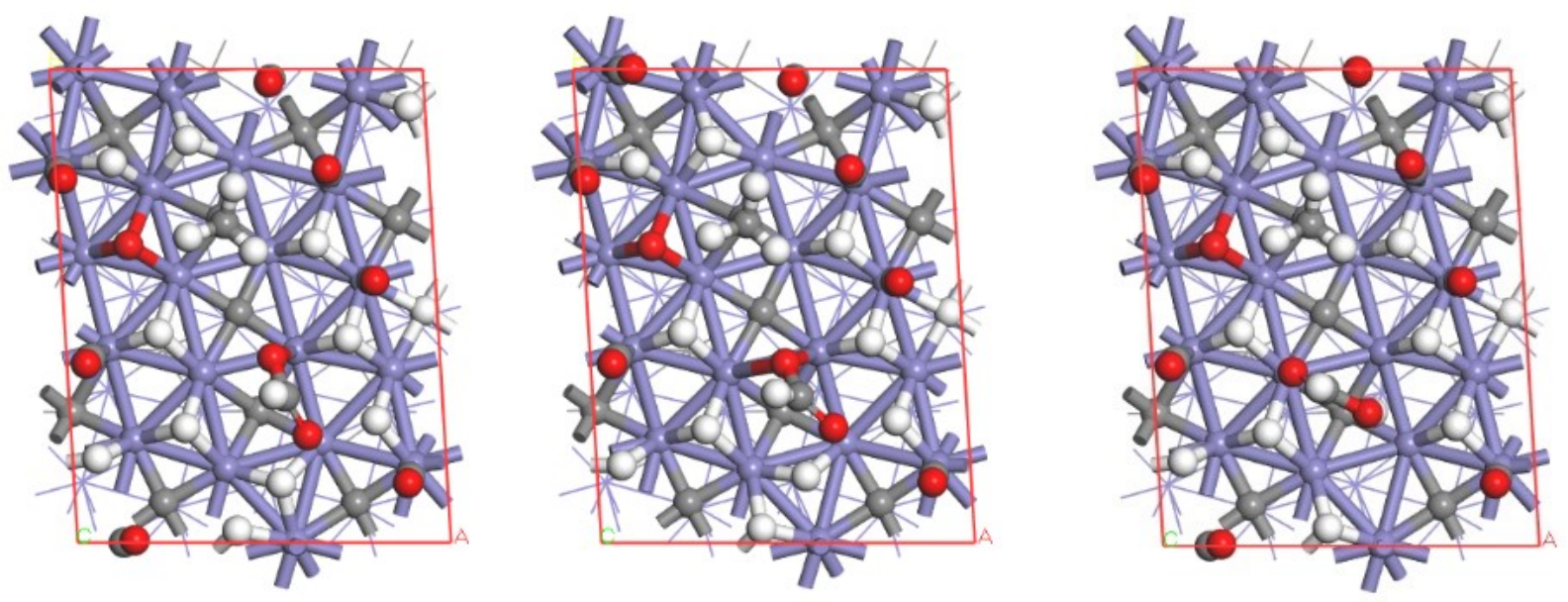

TS11
COOH+H→HCOOH-4

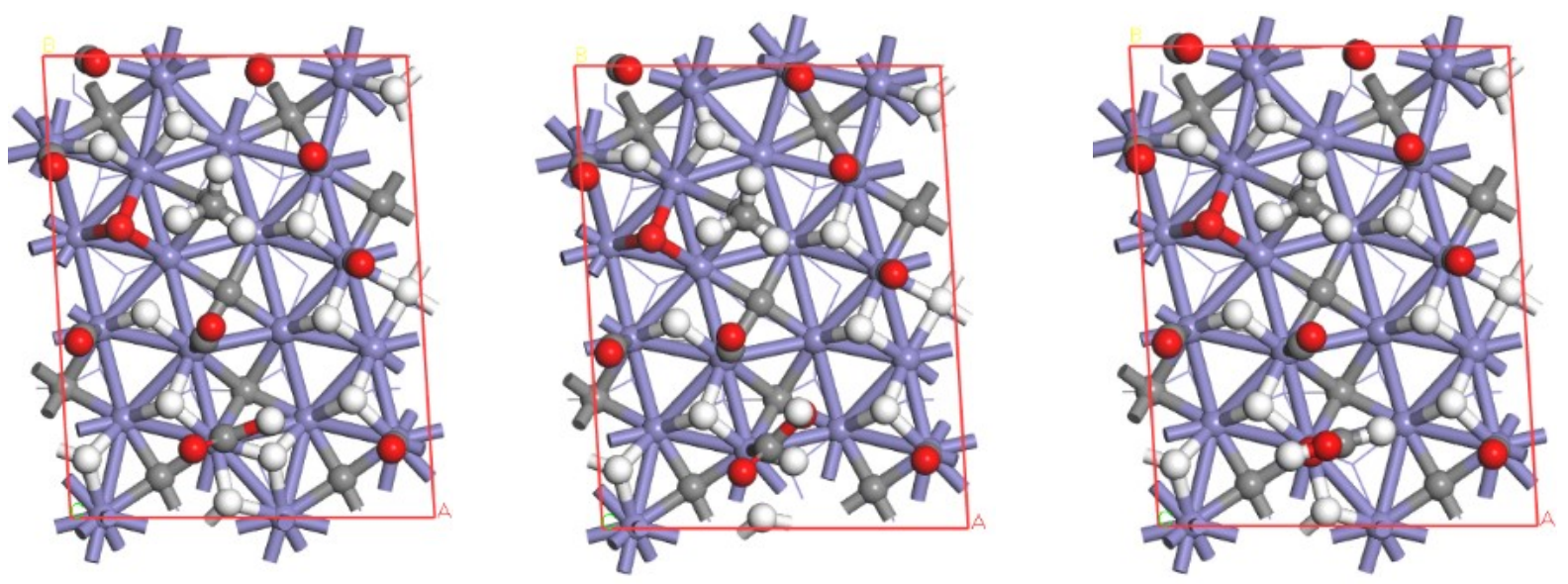

## III. The detail configure of initial state(IS), translate state(TS) and final state(FS) for each elementary reaction in Fig5(b)

| Elementary reaction | Initial state | Translate state | Final state |
|---|---|---|---|
| TS1<br>CO+H→CHO | 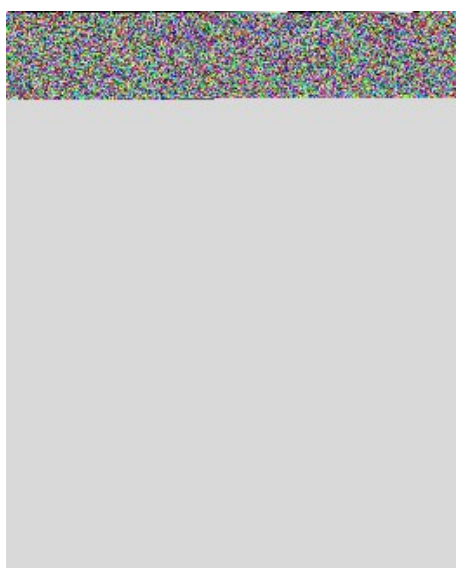 | 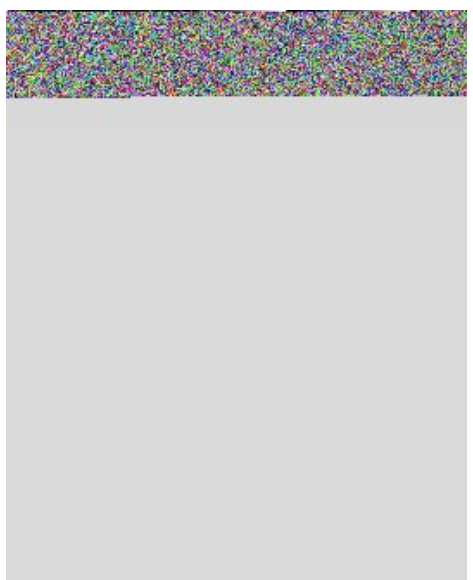 | 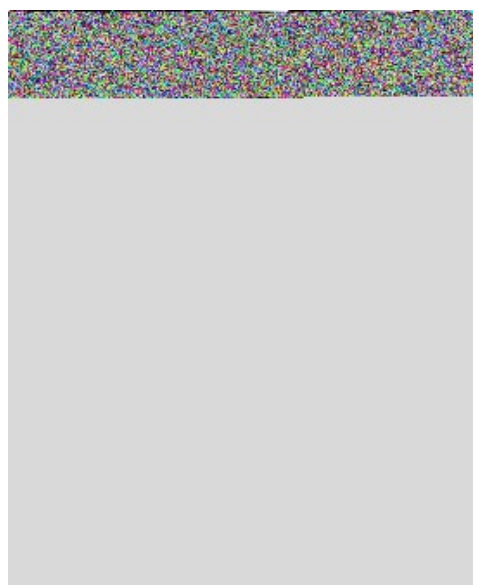 |
| TS2<br>CO+H→COH | 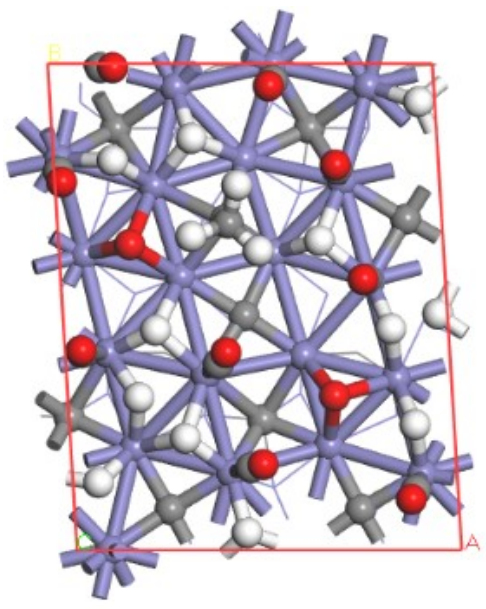 | 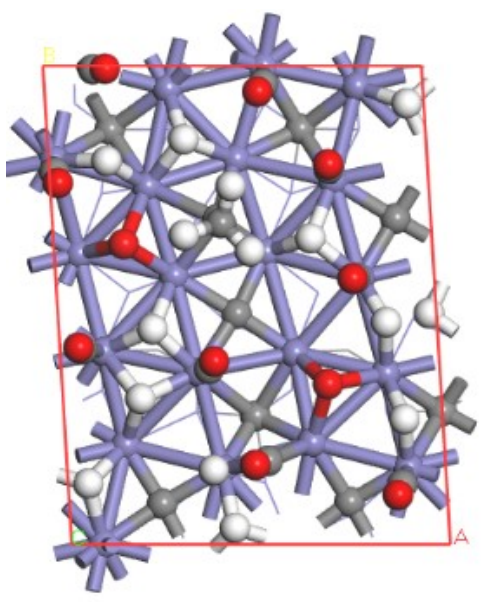 | 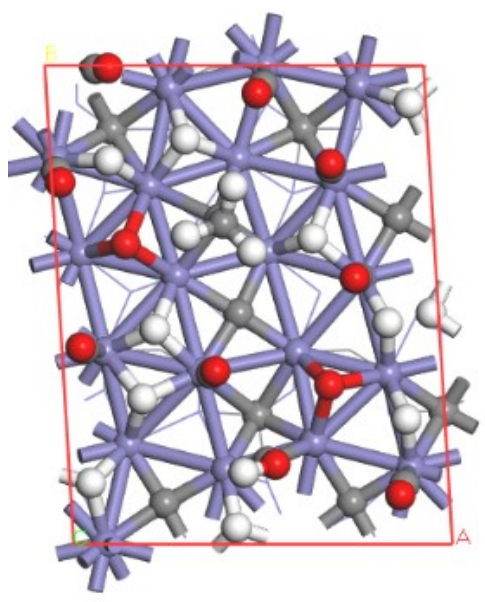 |

TS3

COH+O→COOH

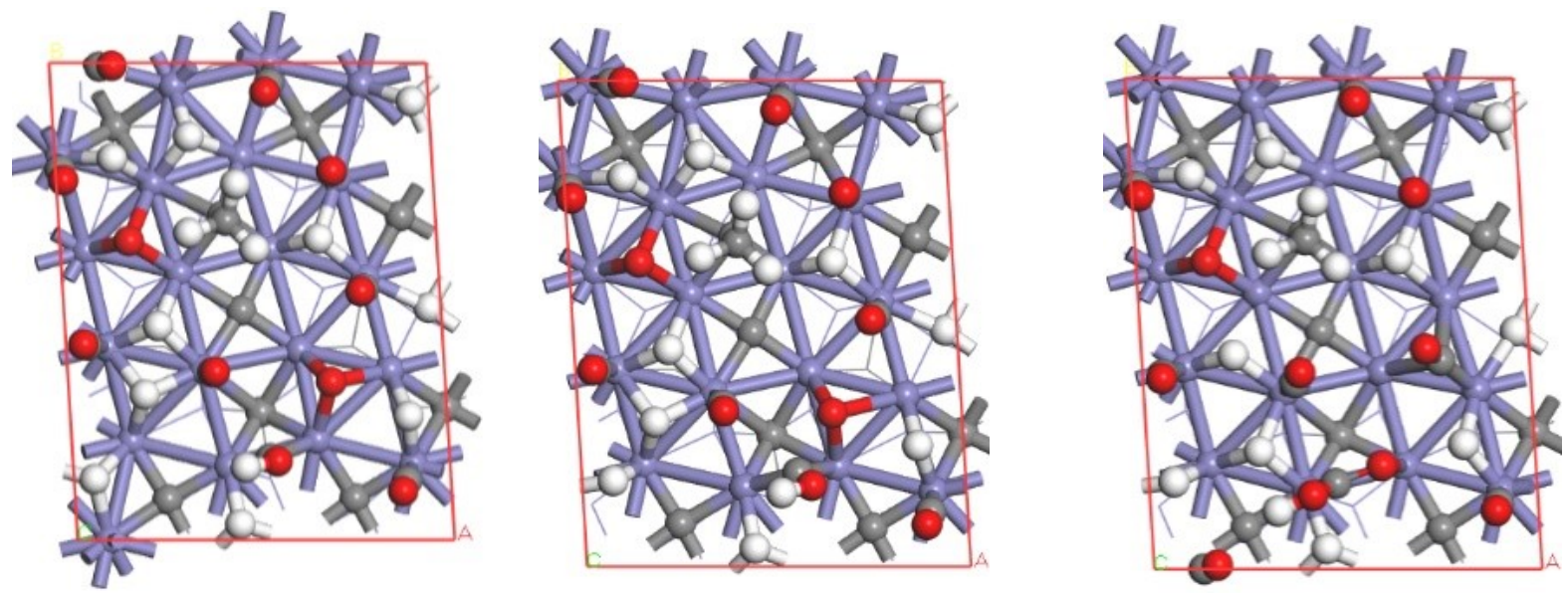

TS4

COH+H→CHOH

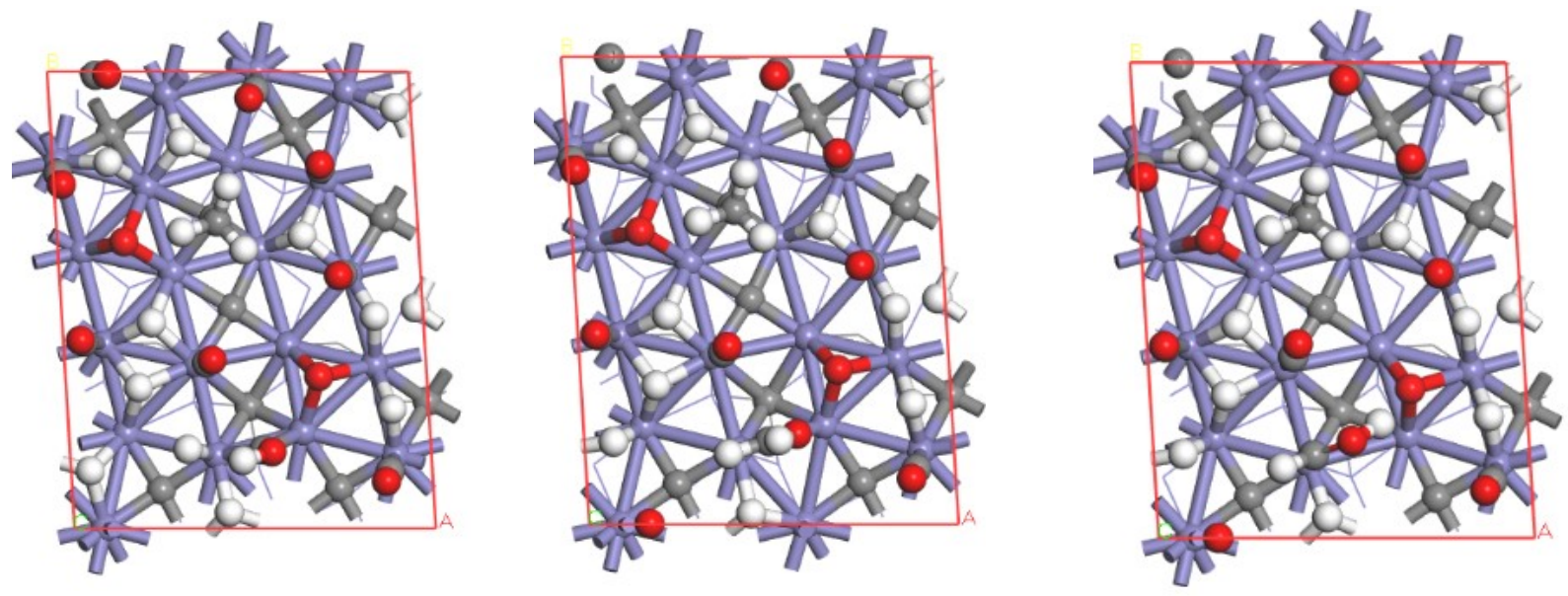

TS5

CHO+H→CHOH

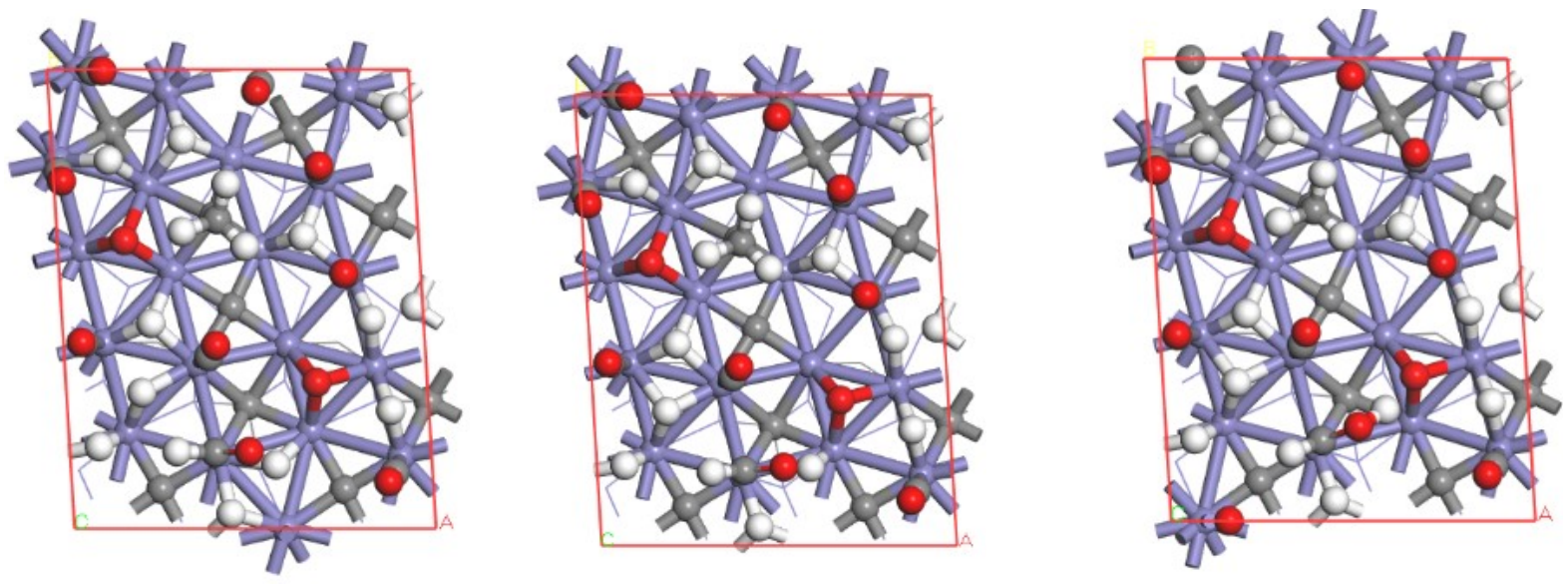

TS6

CHO+O→CHOO

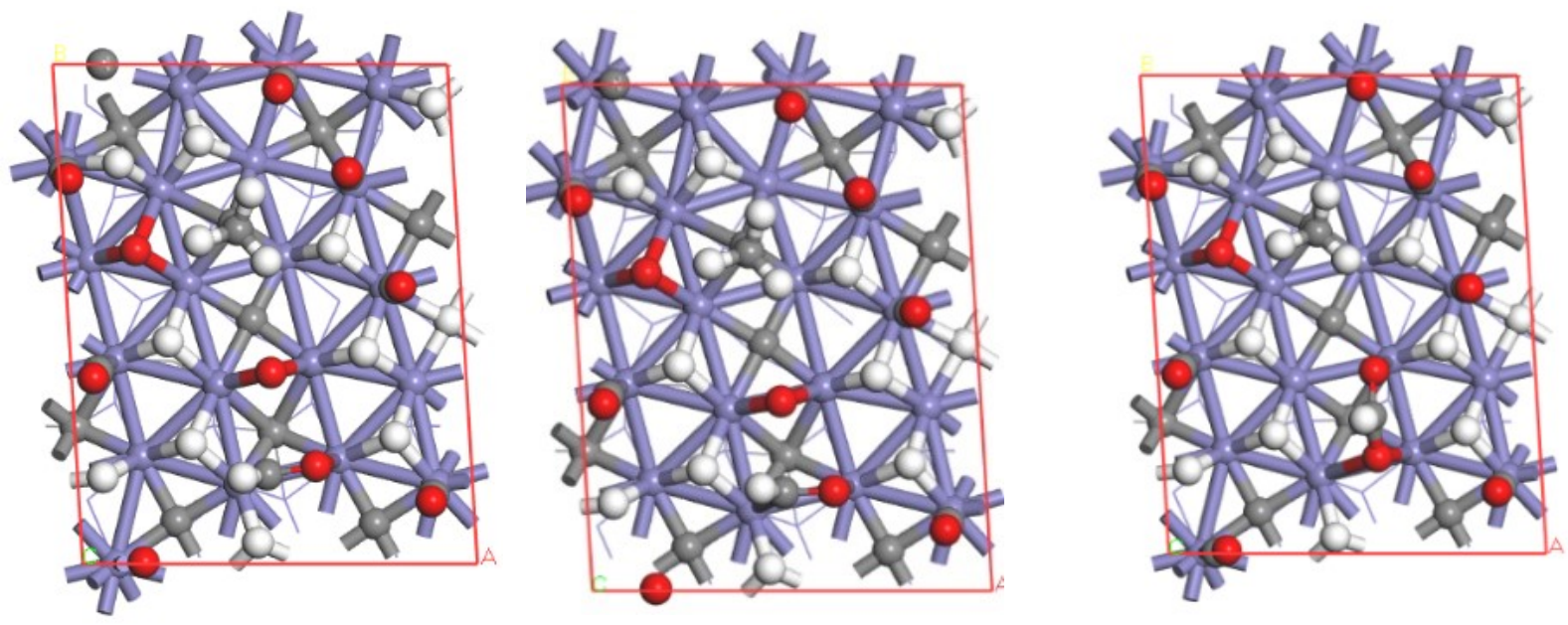

TS7

CHOH+O→HCOOH

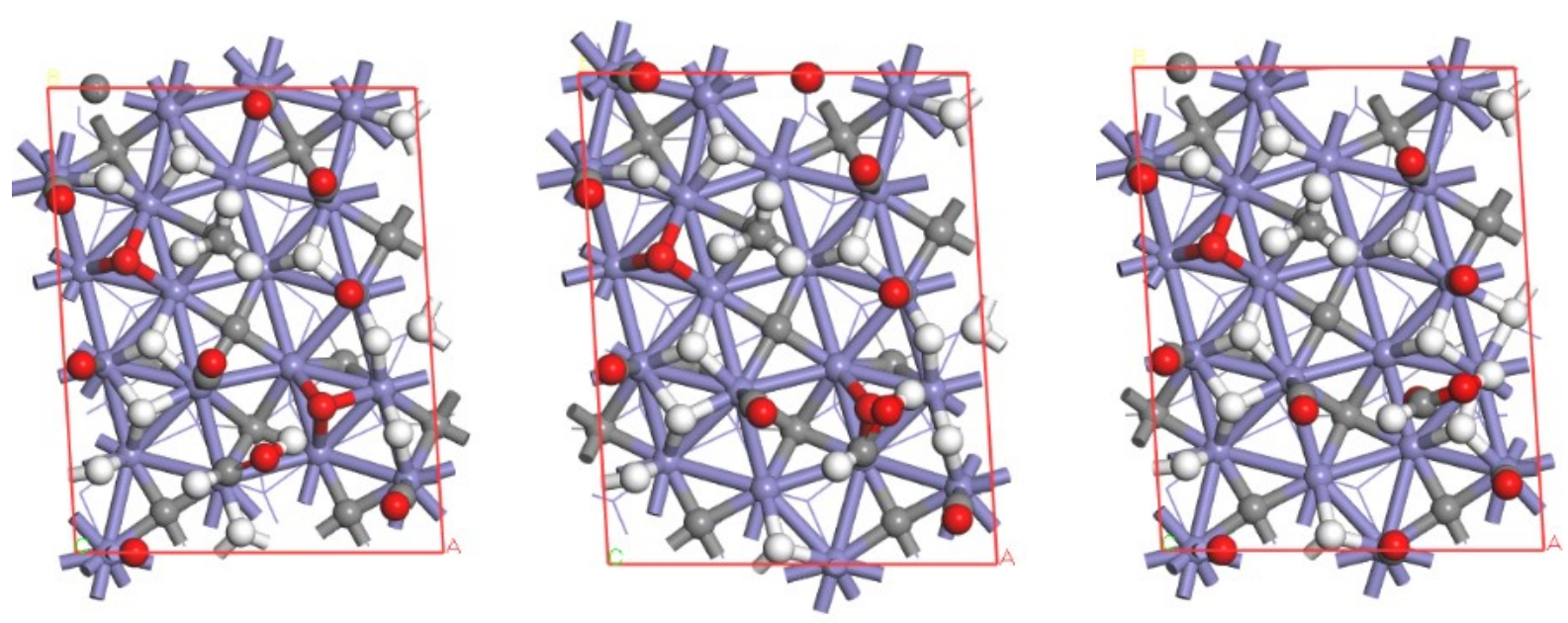

| | | | |
|---|---|---|---|
| TS8<br>CHOH+O→HCOOH | 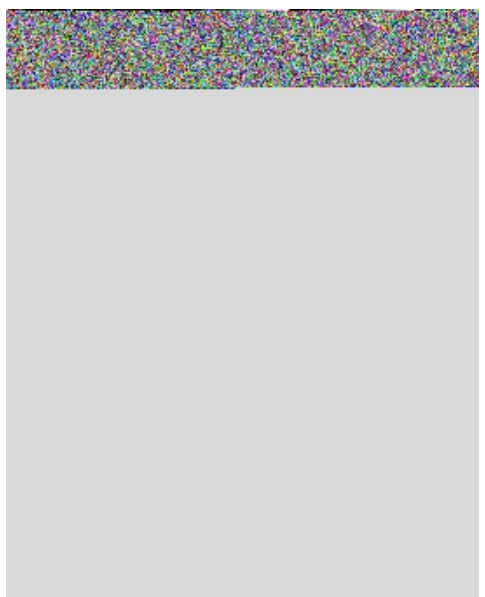 | 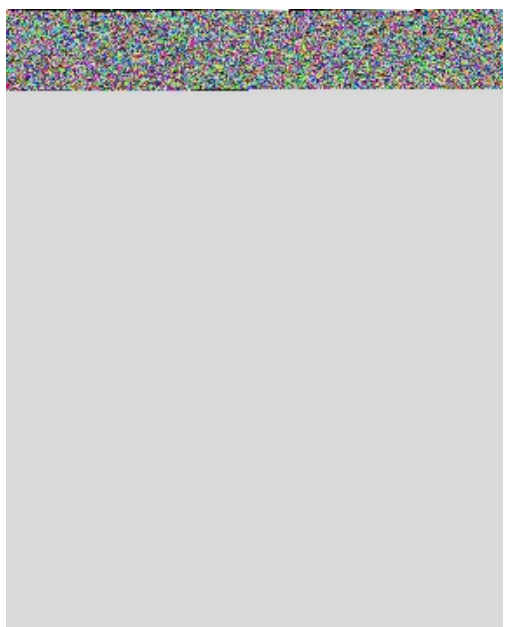 | 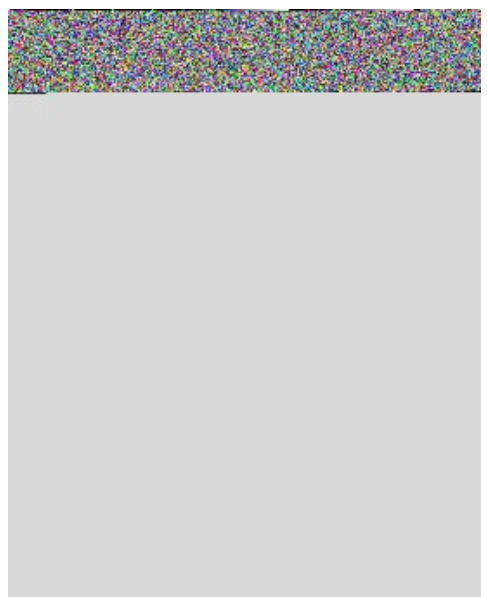 |
| TS9<br>COOH+H→HCOOH | 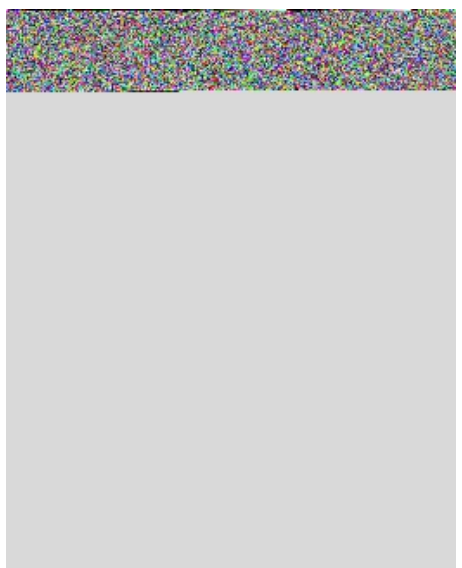 | 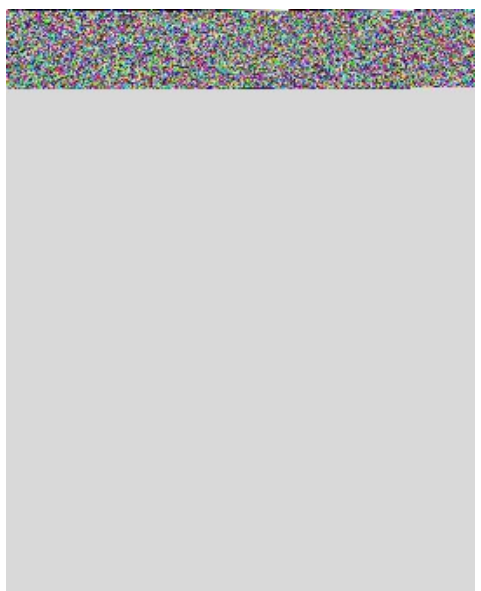 | 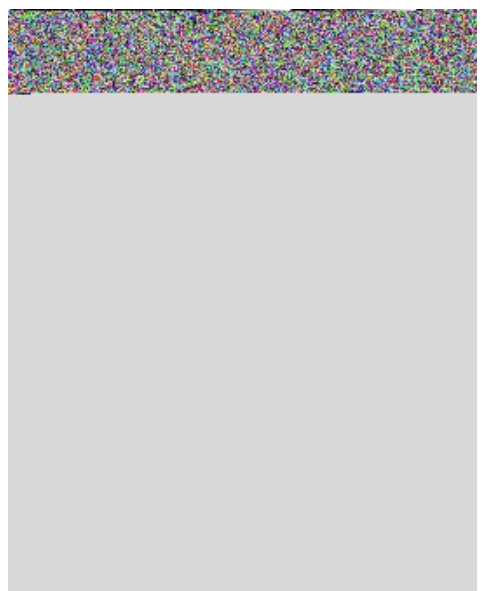 |
| TS10<br>CHOO+H→HCOOH | 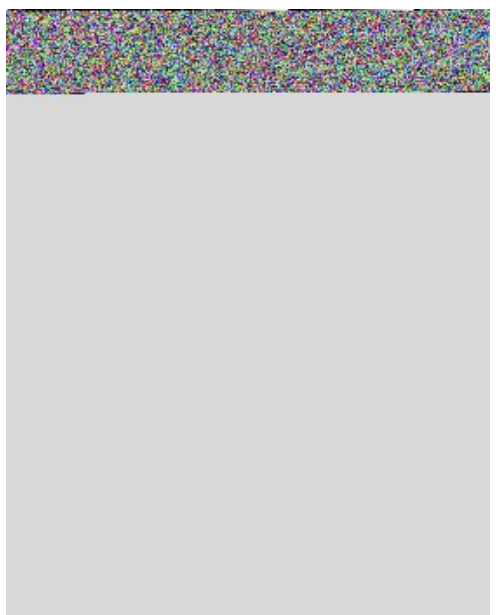 | 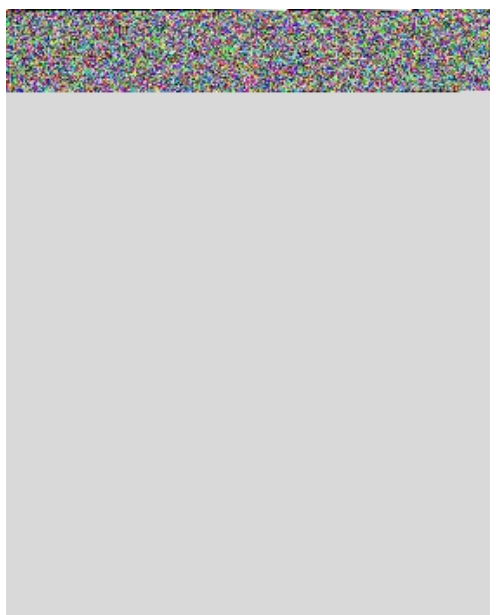 | 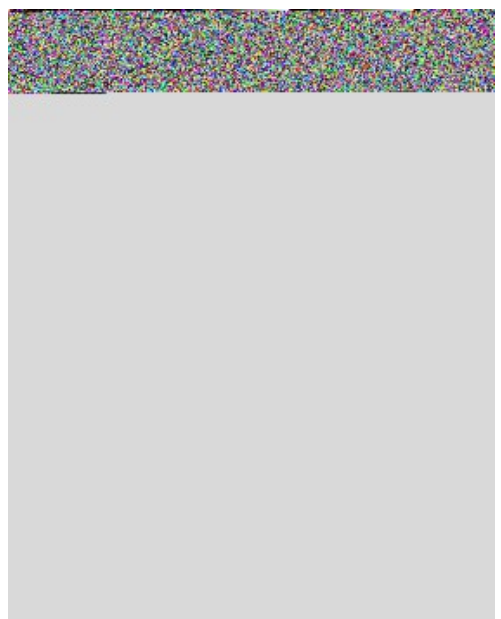 |

## IV. The detail configure of initial state(IS), translate state(TS) and final state(FS) for each elementary reaction in Fig6(a)

| Elementary reaction | Initial state | Translate state | Final state |
|---|---|---|---|
| TS1<br>CO+H→COH | 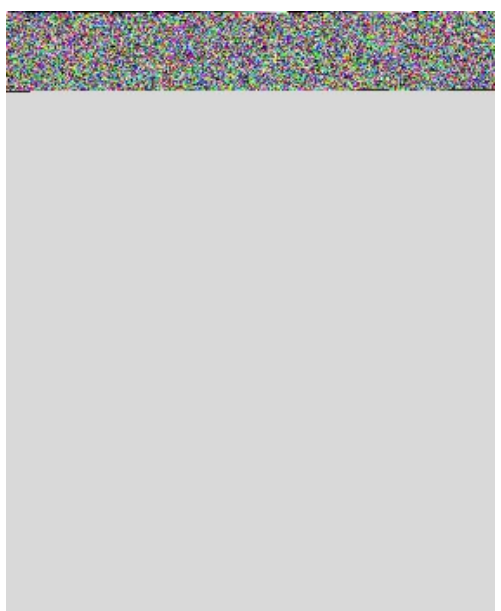 | 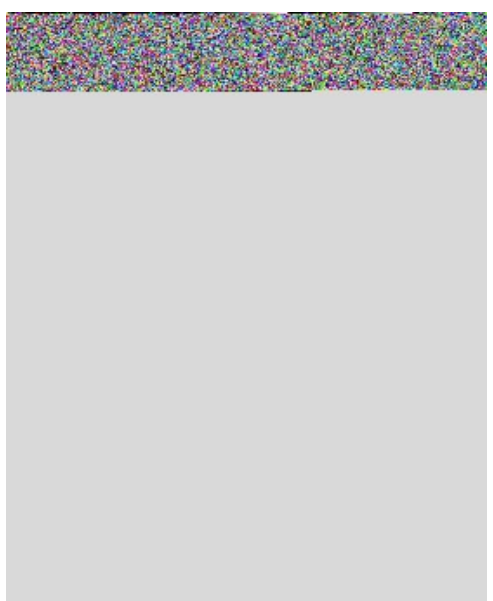 | 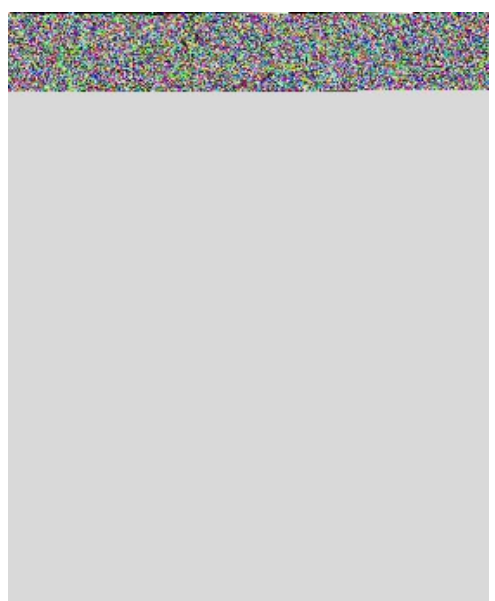 |

TS2

$CO+O \rightarrow CO_2$

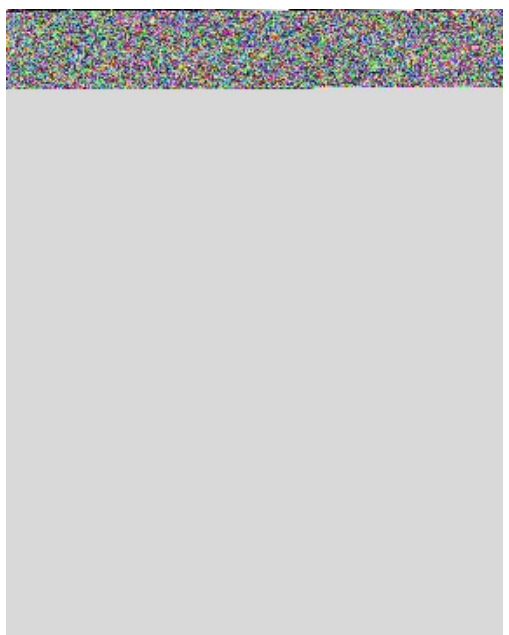
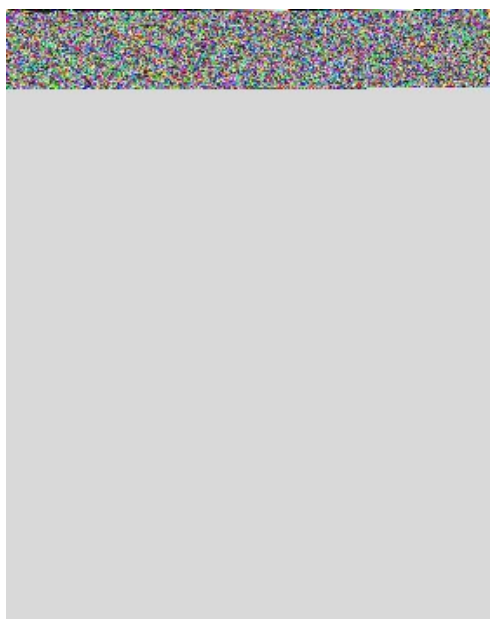
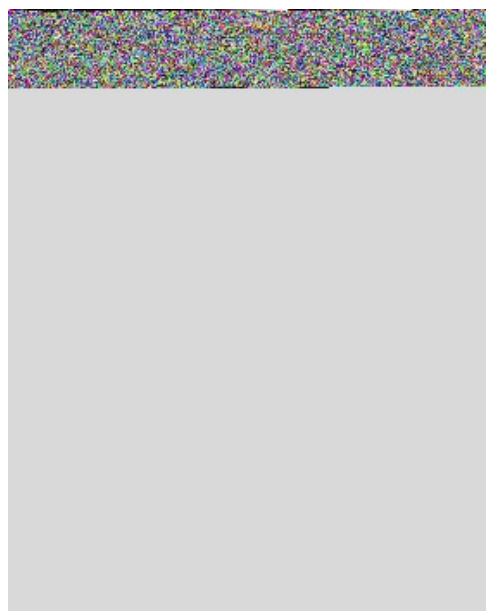

TS3

$CO+H \rightarrow CHO$

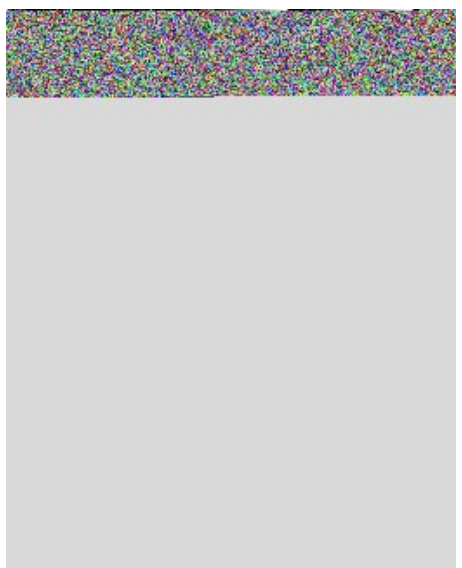
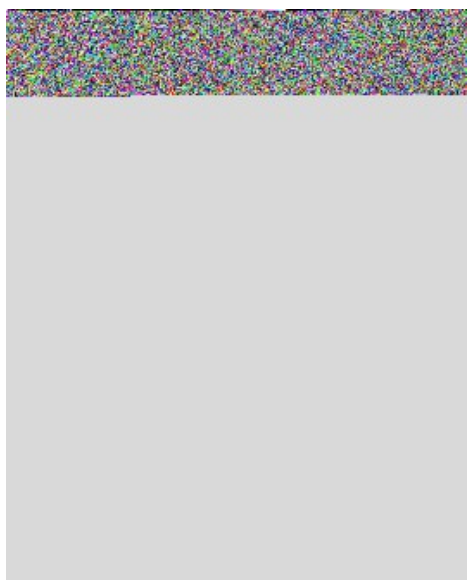
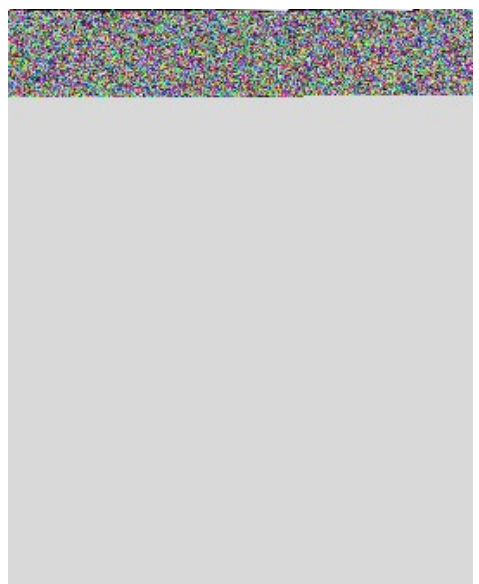

TS4

$O+H \rightarrow OH$

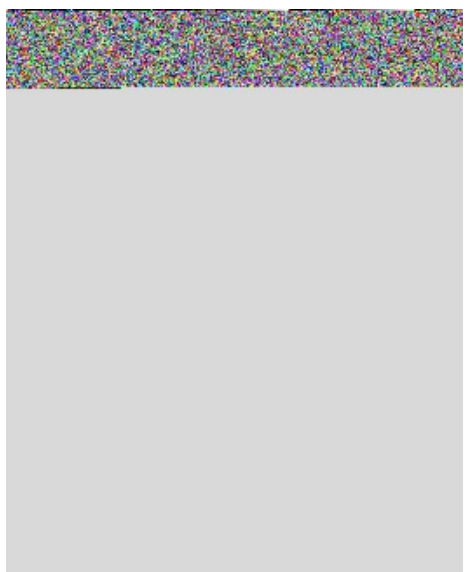
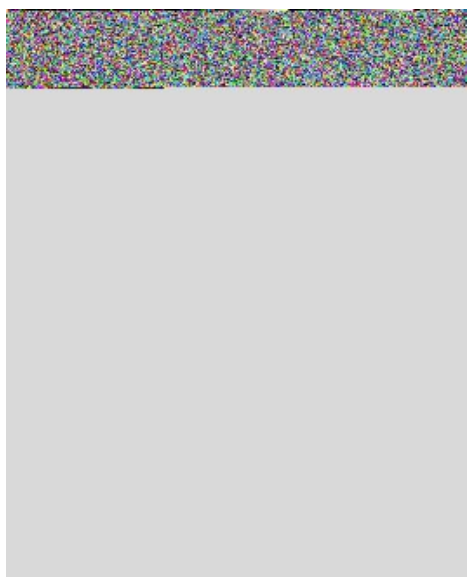
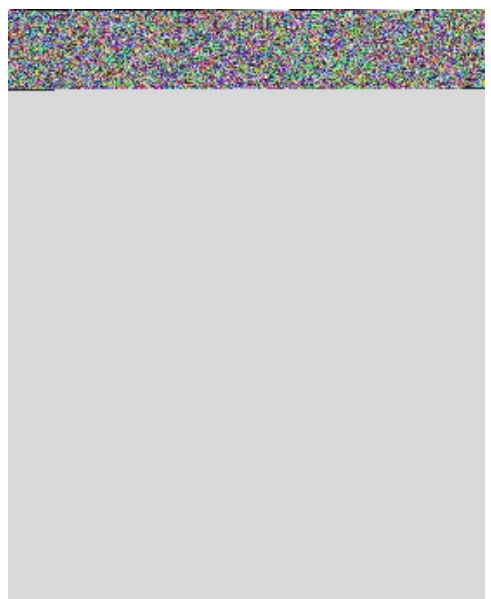

TS5

$COH+O \rightarrow COOH$

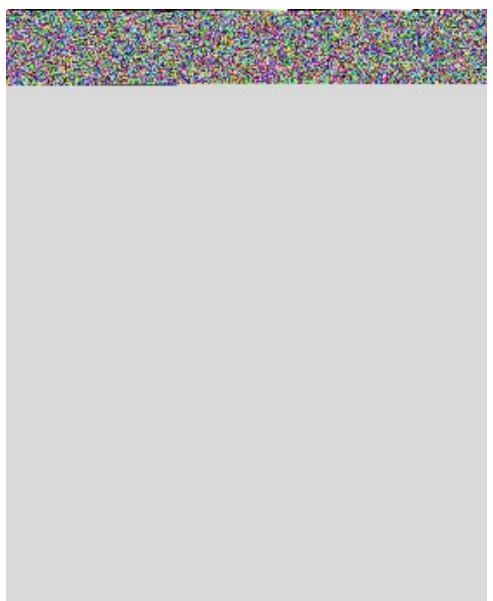
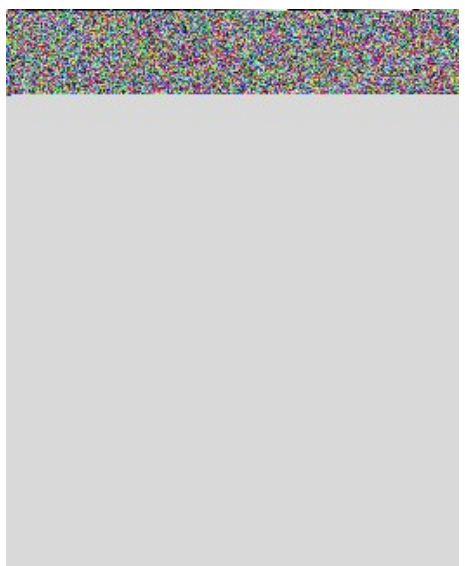
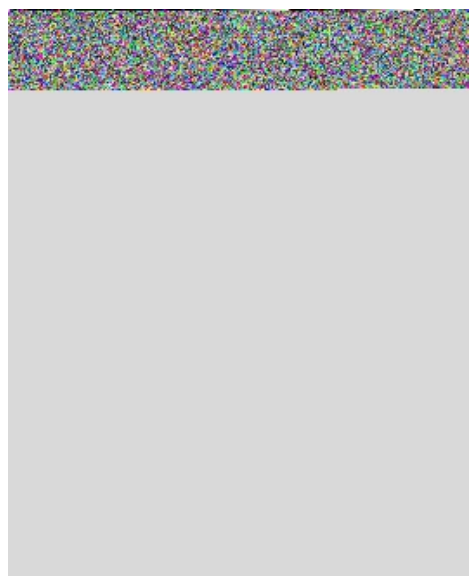

TS6

$CHO+O \rightarrow CHOO$

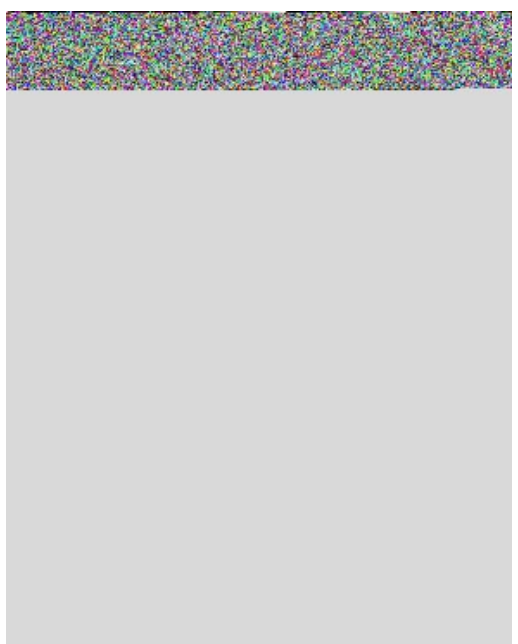
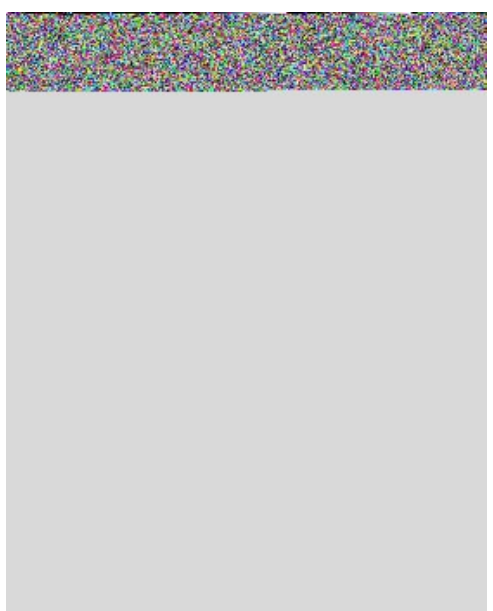
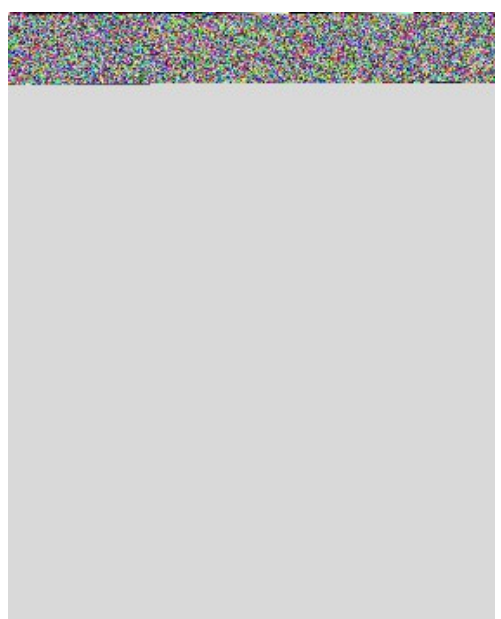

TS7

$OH+H \rightarrow H_2O$

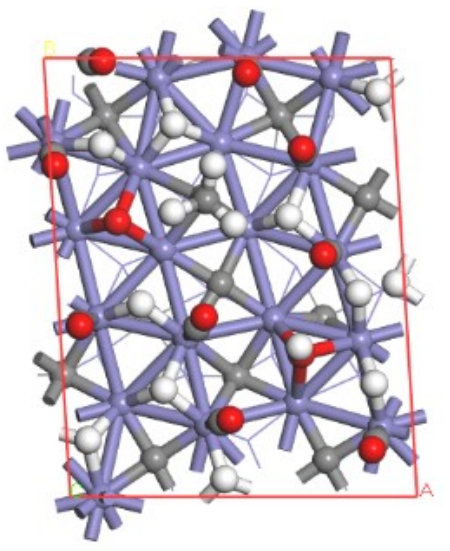 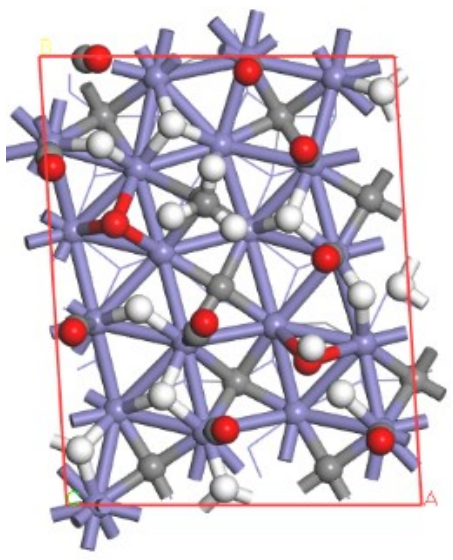 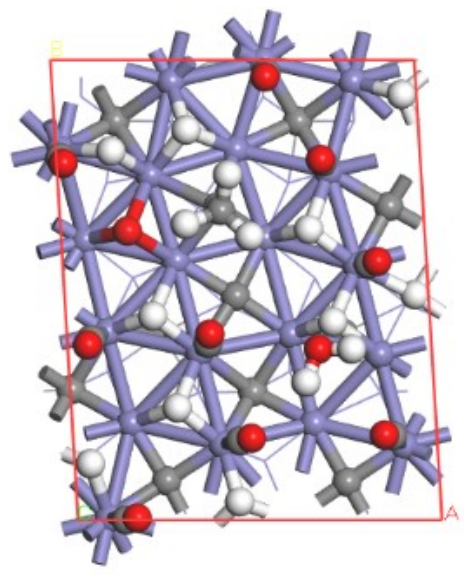

TS8

$CO+OH \rightarrow COOH$

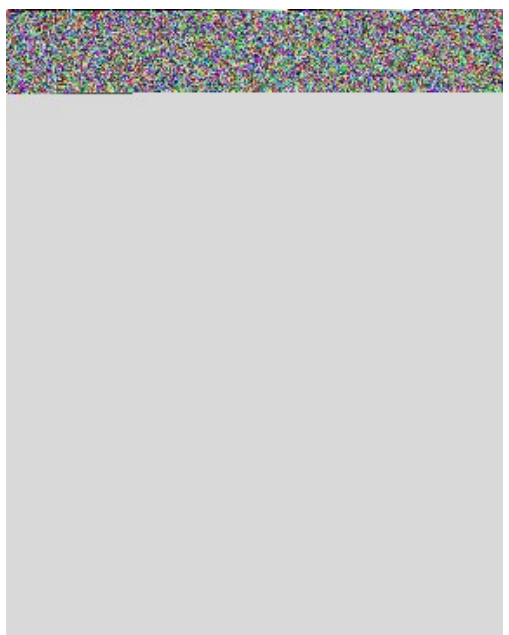 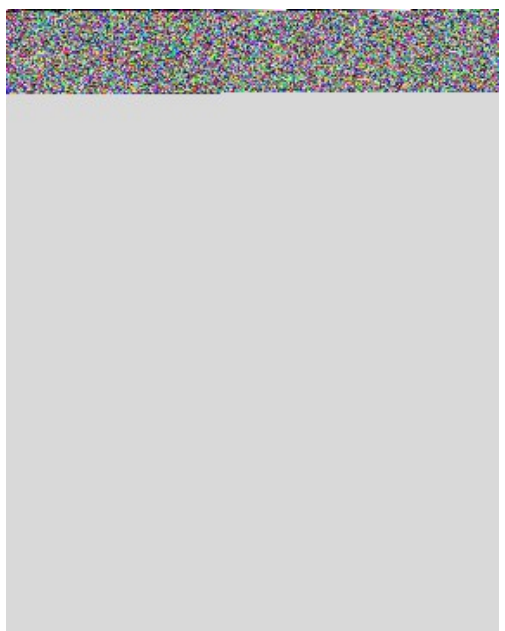 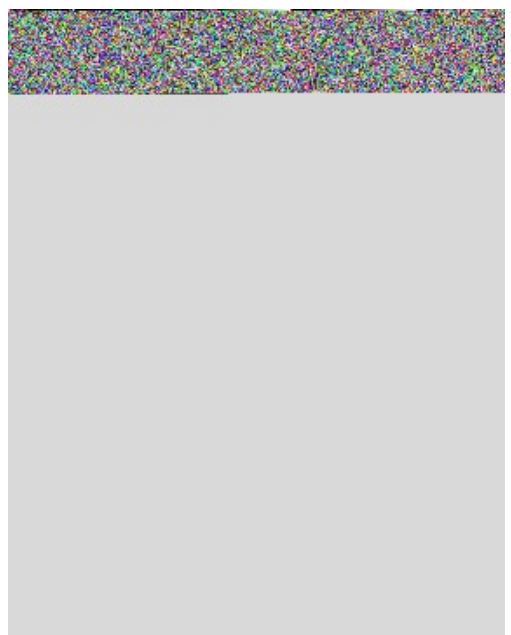

TS9

$CHOO \rightarrow CO_2+H$

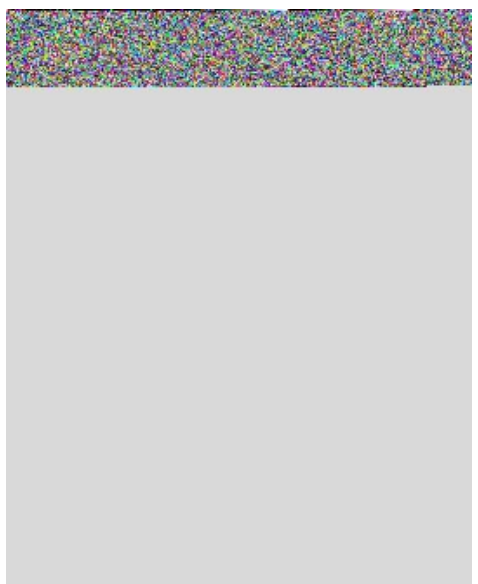 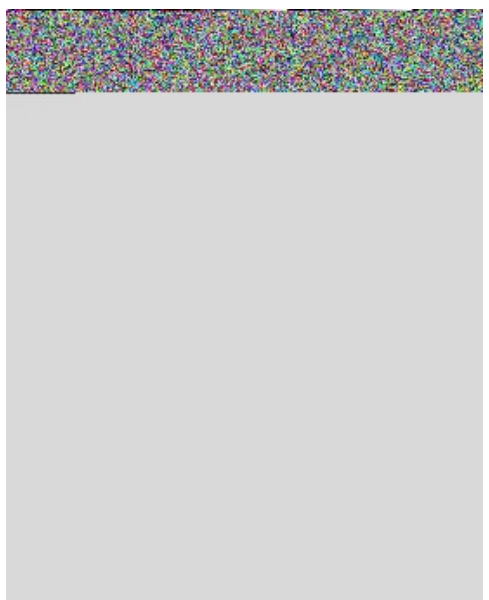 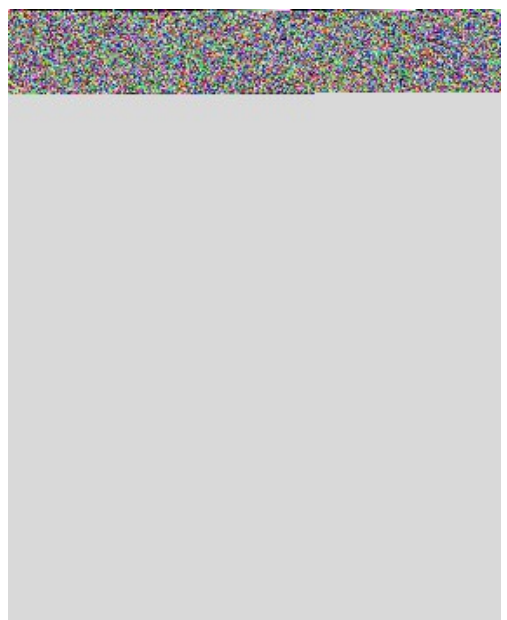

TS10

$COOH \rightarrow CO_2+H$

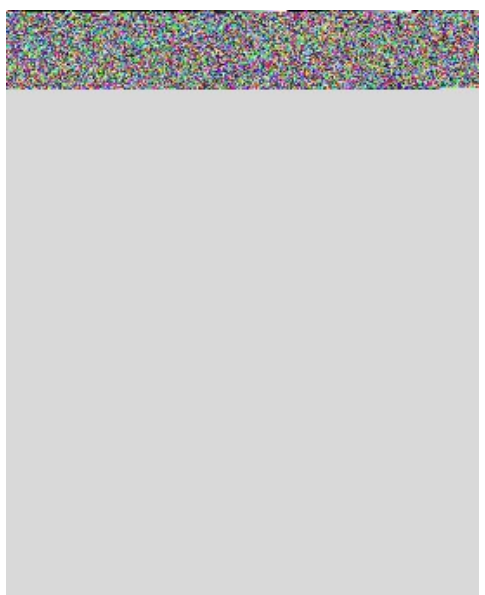 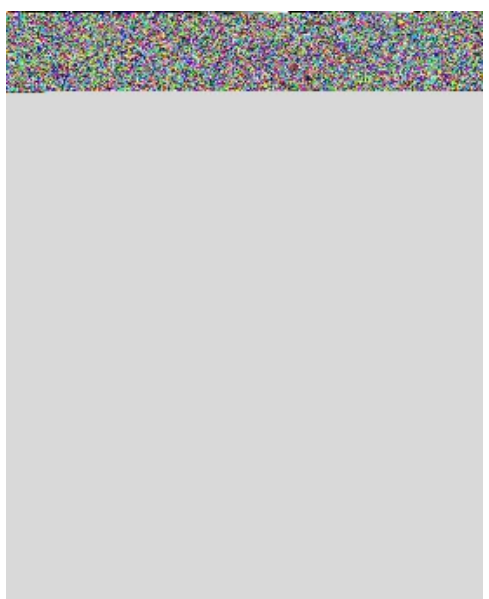 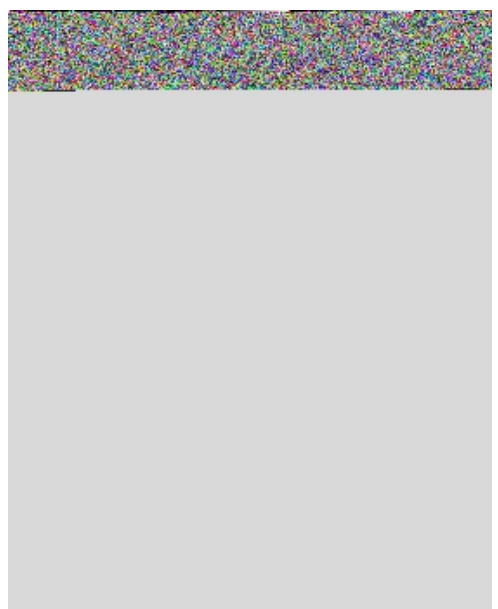

TS11

$COOH \rightarrow CO_2+H$

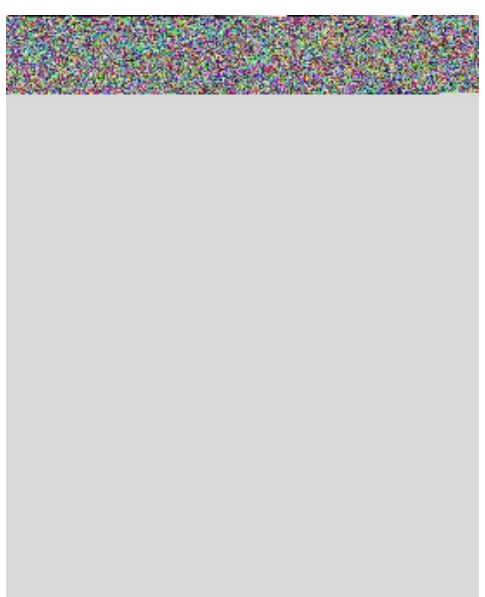 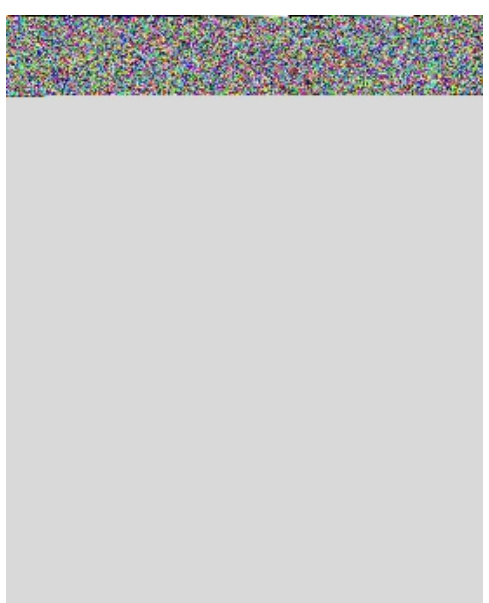 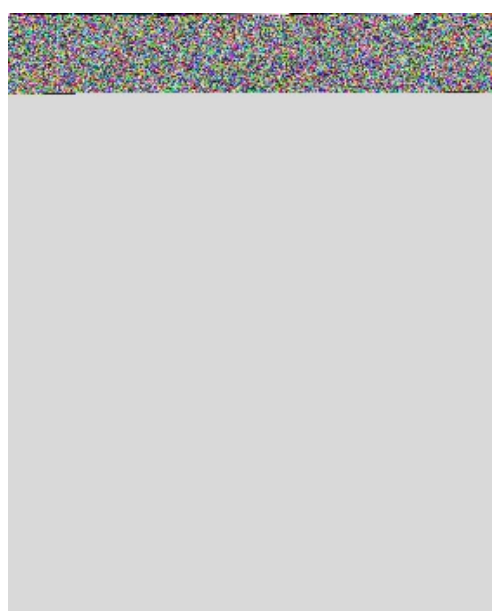

## V. The detail configure of initial state(IS), translate state(TS) and final state(FS) for each elementary reaction in Fig6(b)

| Elementary reaction | Initial state | Translate state | Final state |
|---|---|---|---|
| TS1<br>CO+H→COH | 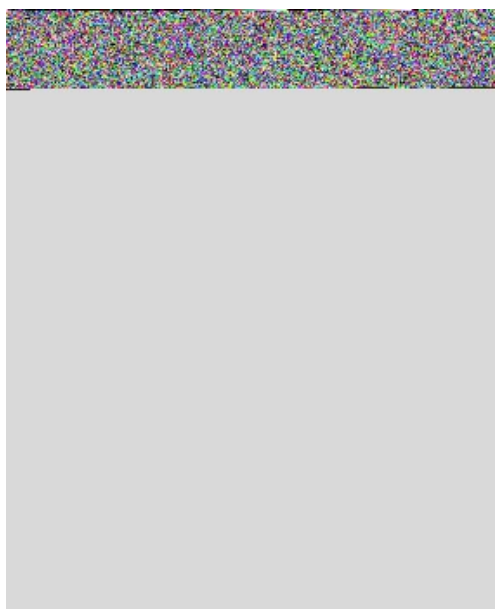 | 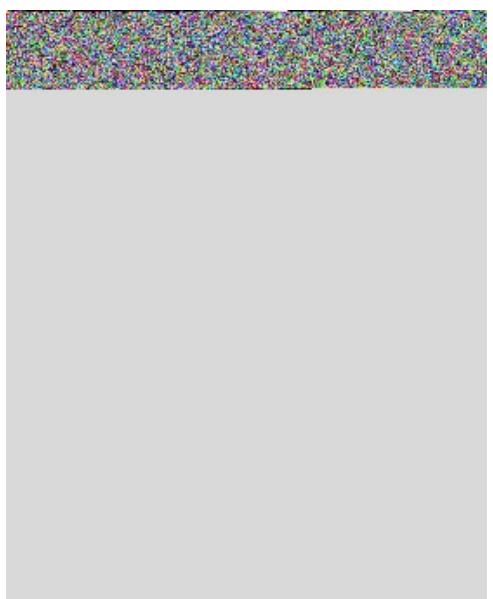 | 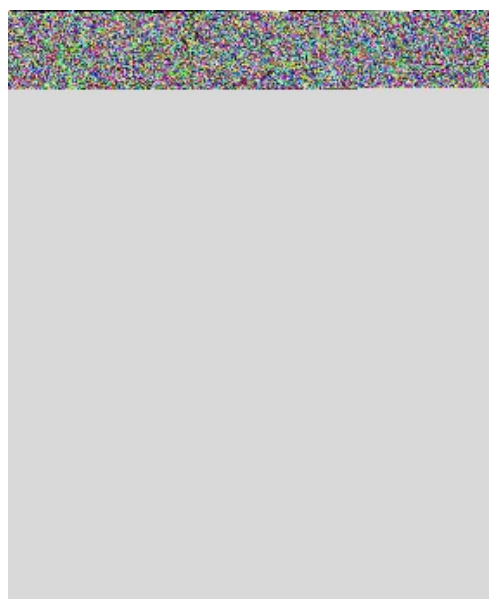 |

TS2

CO+H→CHO

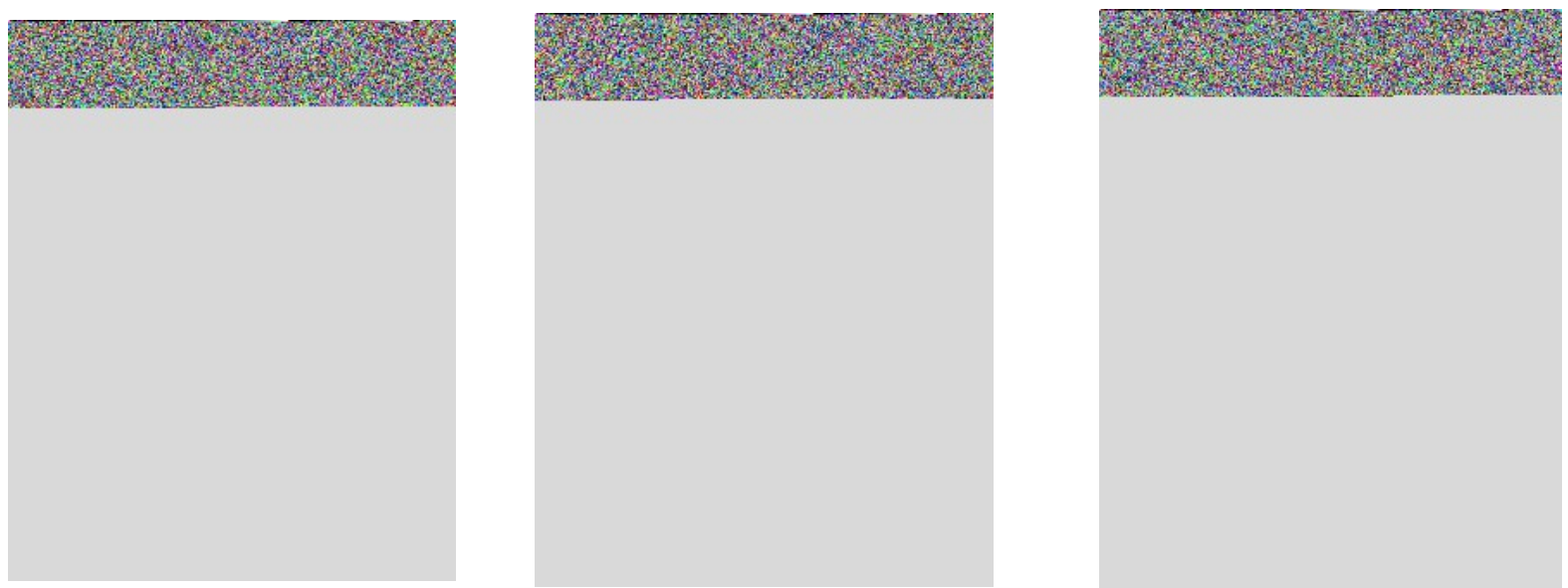

TS3

COH+H→CHOH

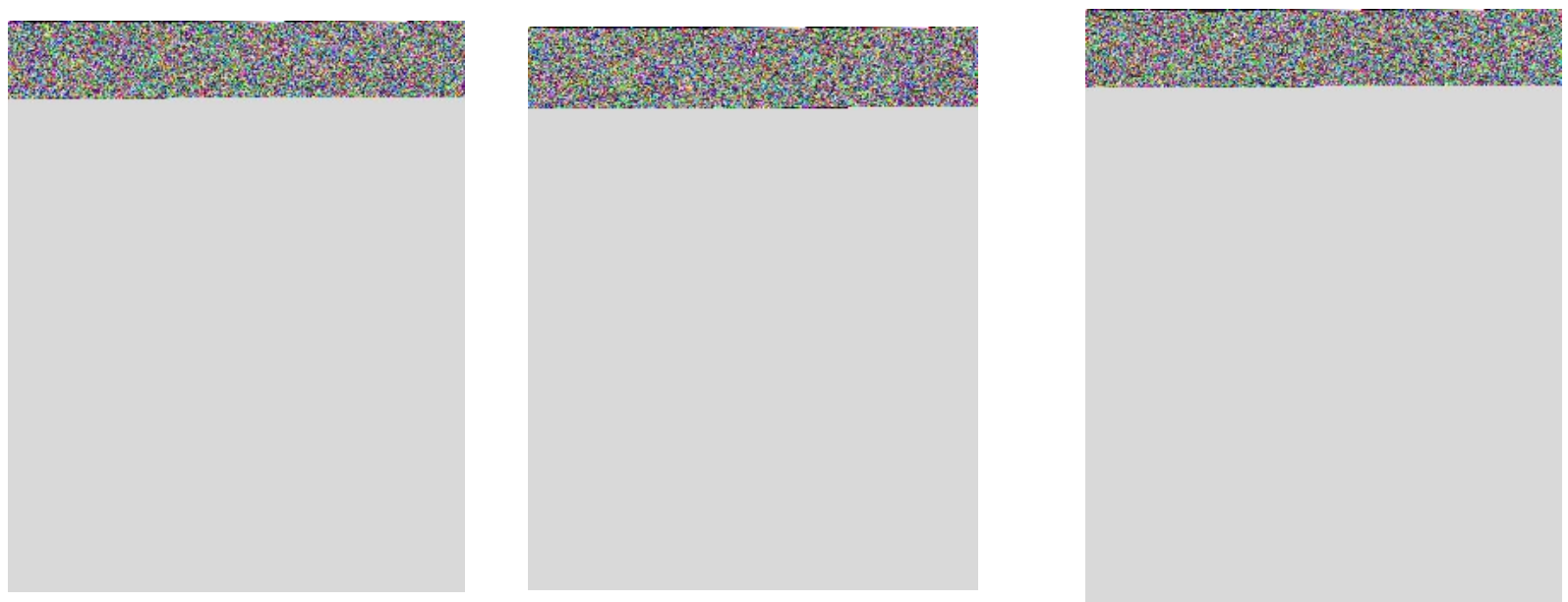

TS4

CHO+H→CHOH

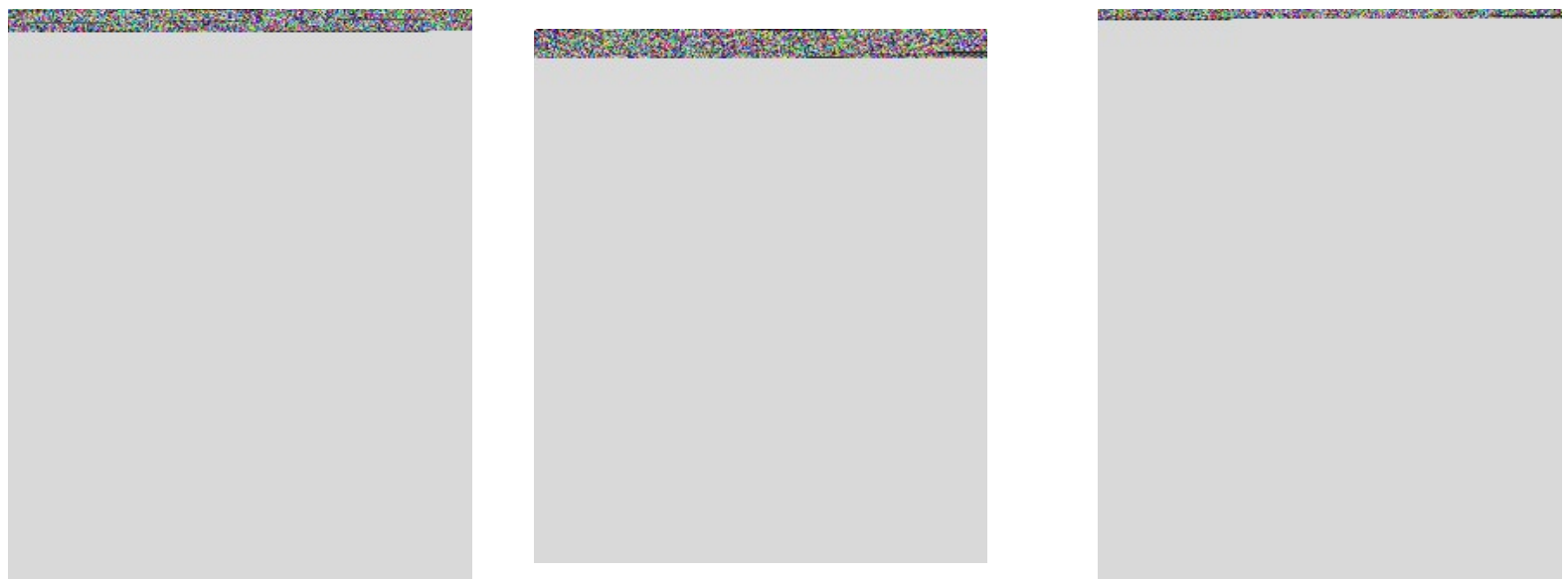

TS5

CHO+H→$CH_2O$

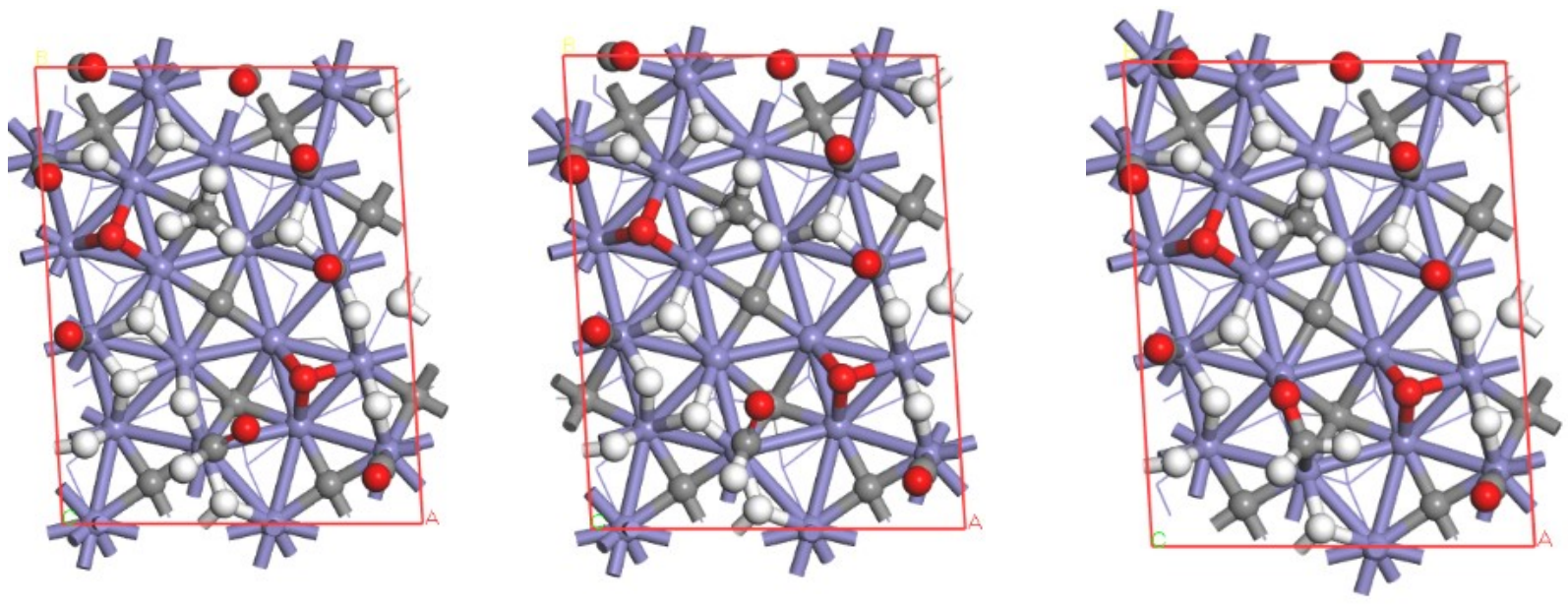

TS6

$CH_2O$+H→$CH_2OH$

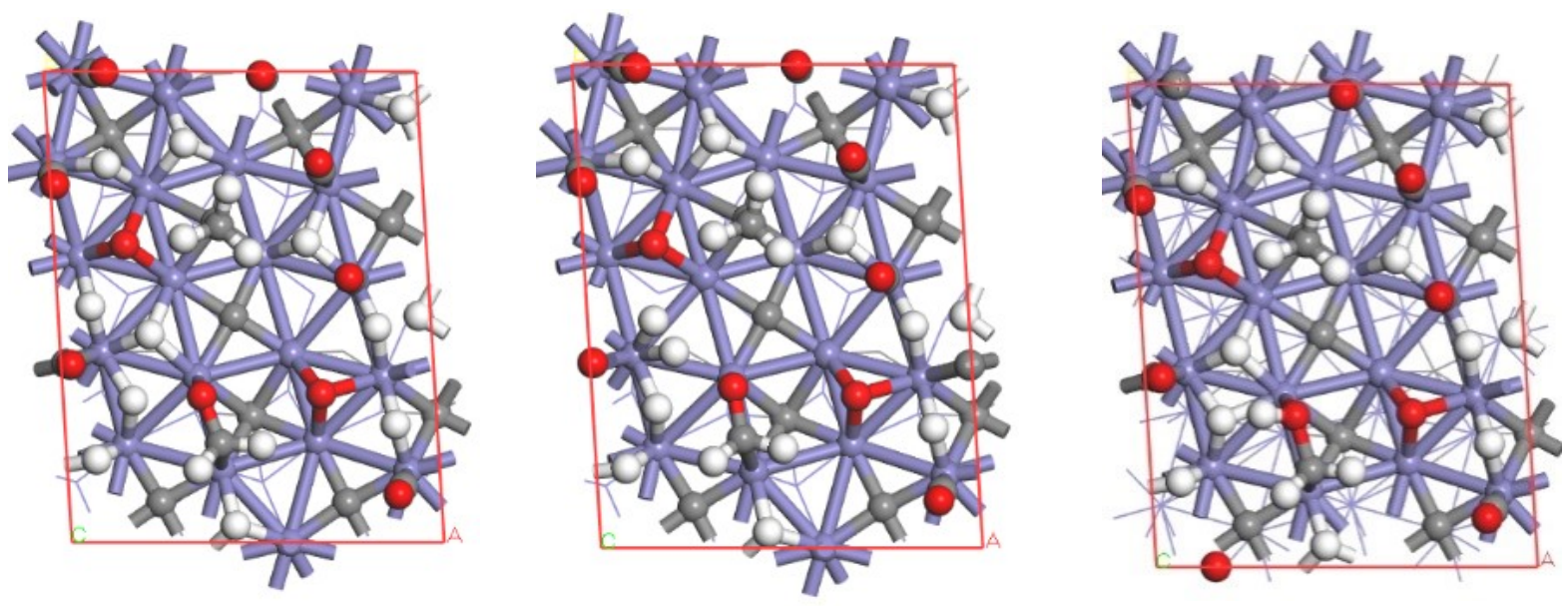

TS7

$CH_2O$+H→$CH_2OH$

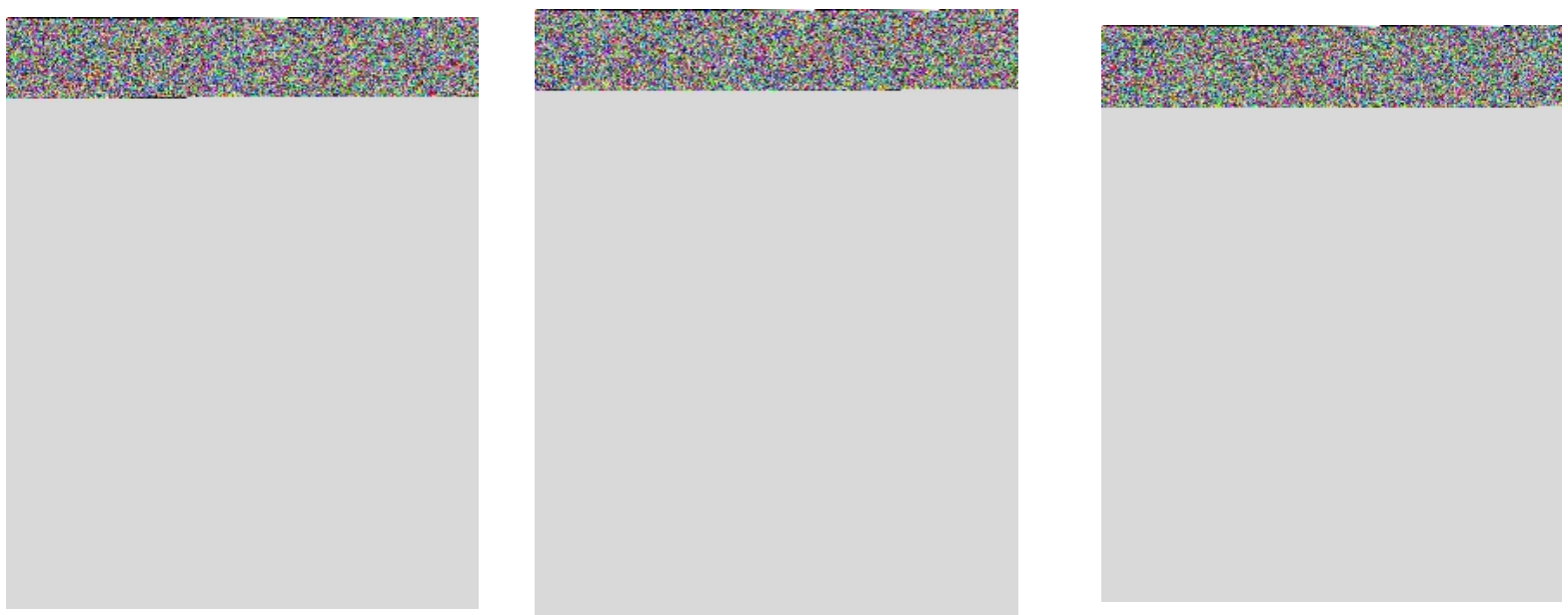

TS8

$CH_2O$+H→$CH_2OH$

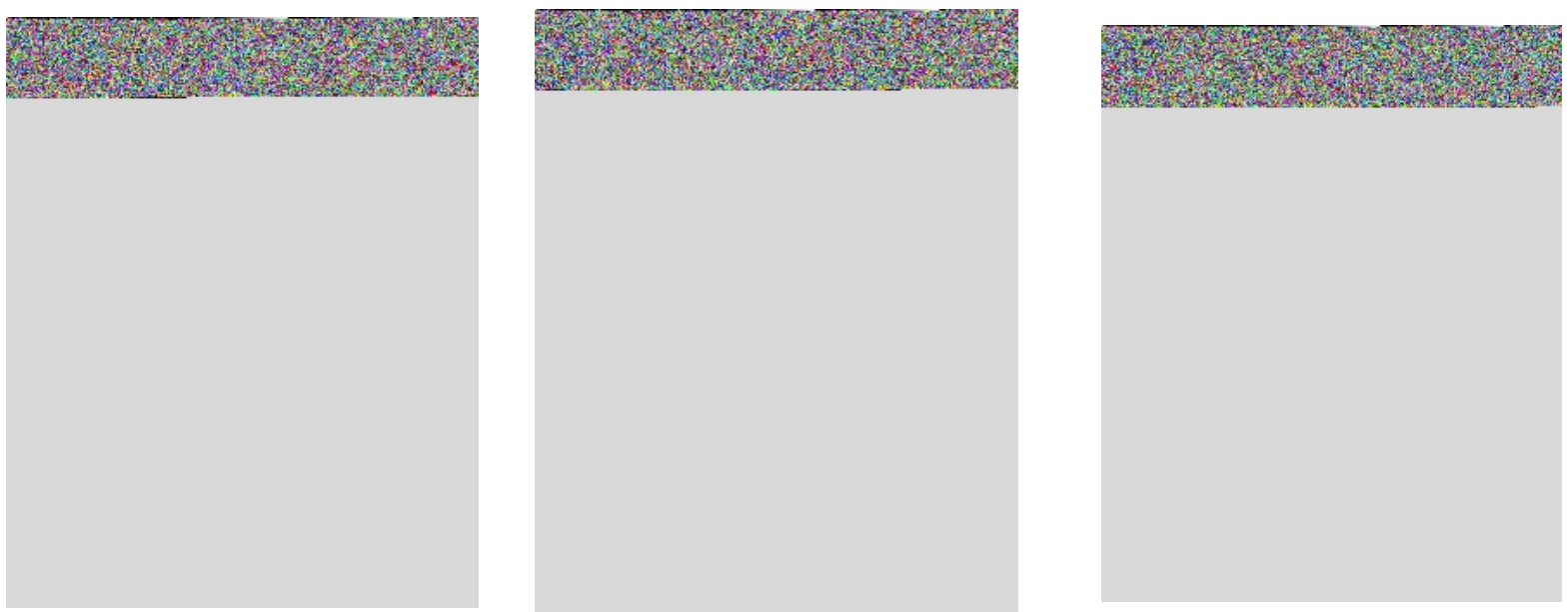

TS9

$CH_2O$+H→$CH_3O$

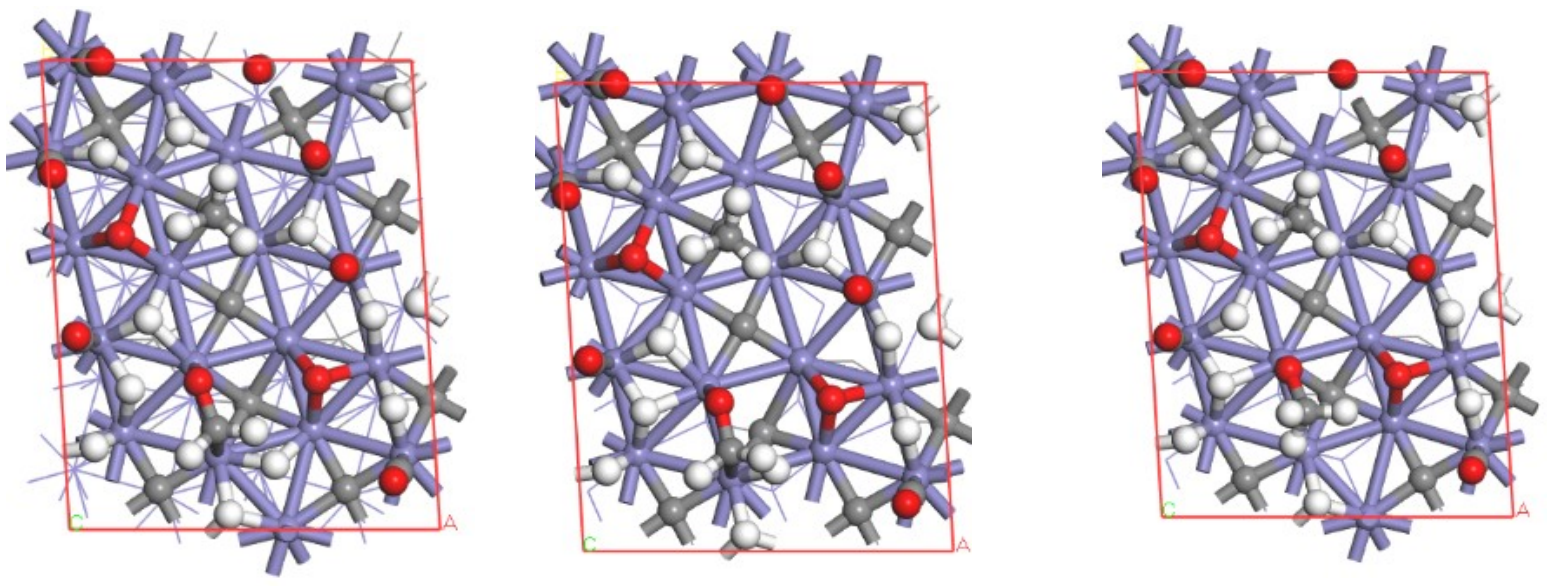

TS10

$CH_3O$+H→$CH_3OH$

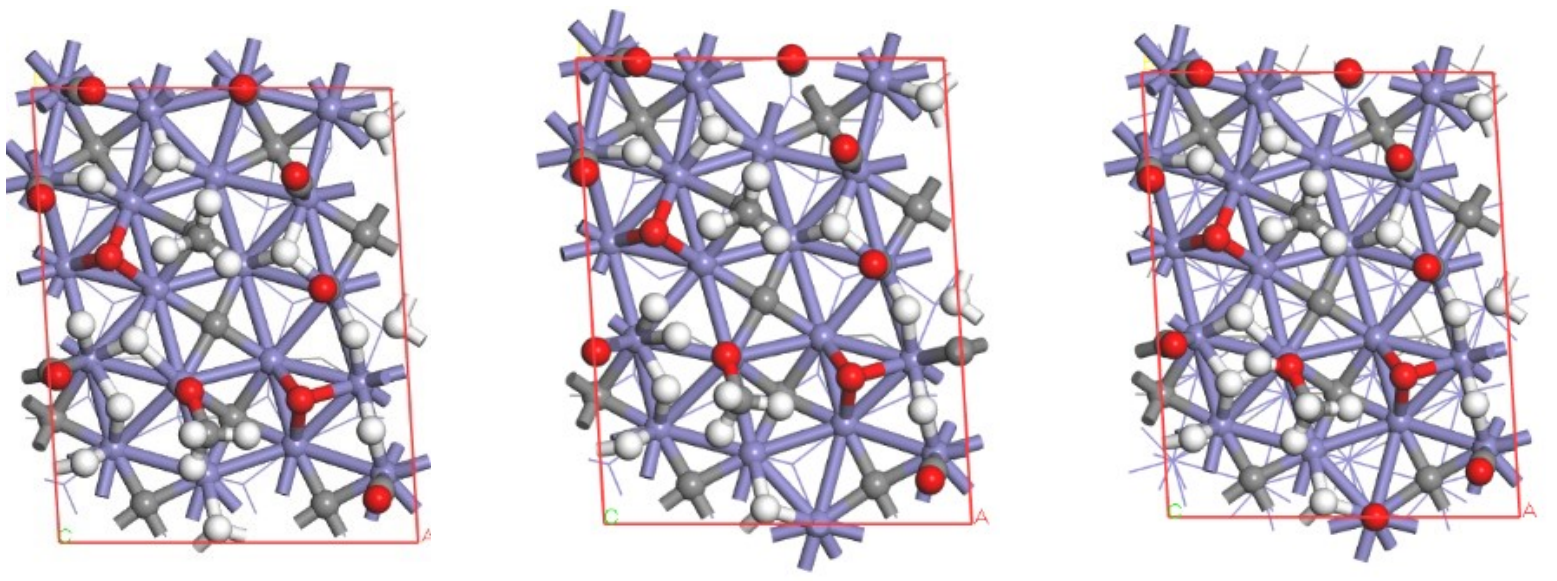

TS11

$CH_2OH$+H→$CH_3OH$

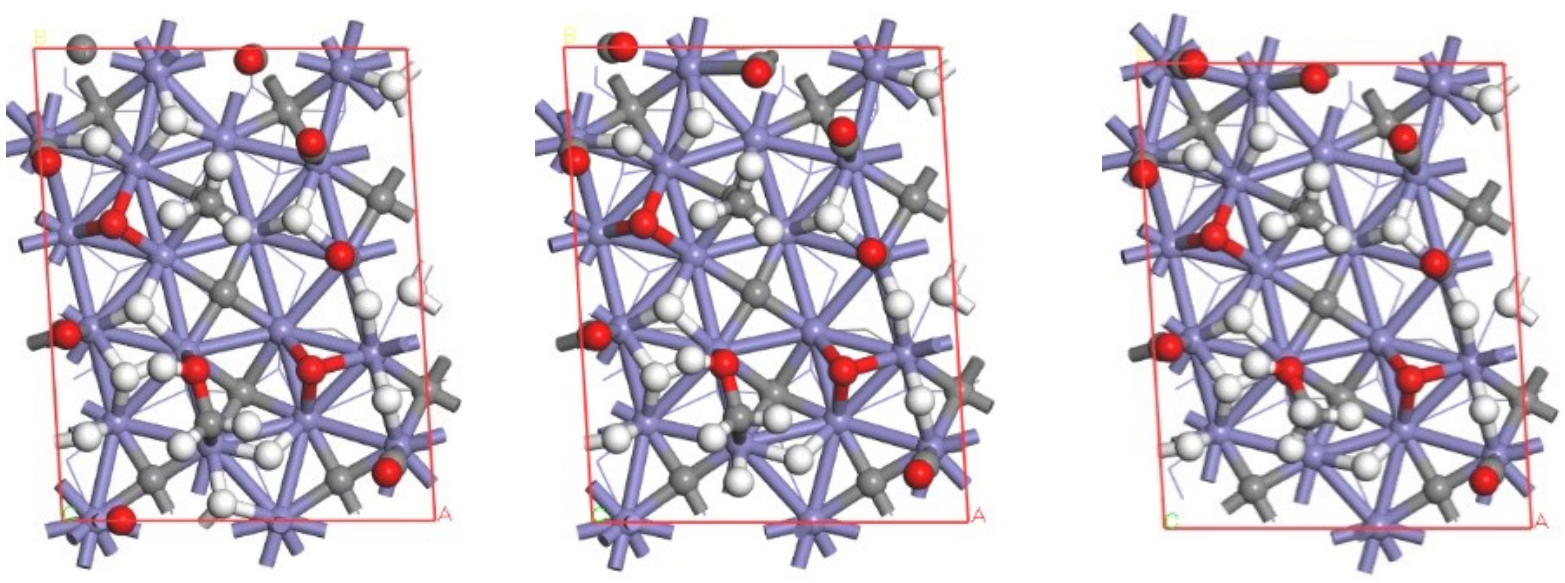

| | | | |
|---|---|---|---|
| TS12<br>$CH_2OH+H \rightarrow CH_3OH$ | 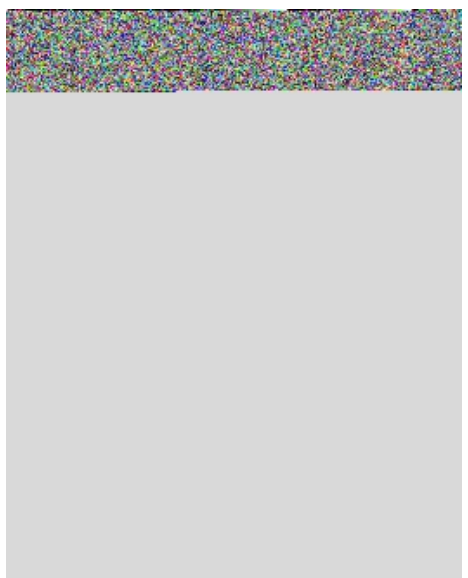 | 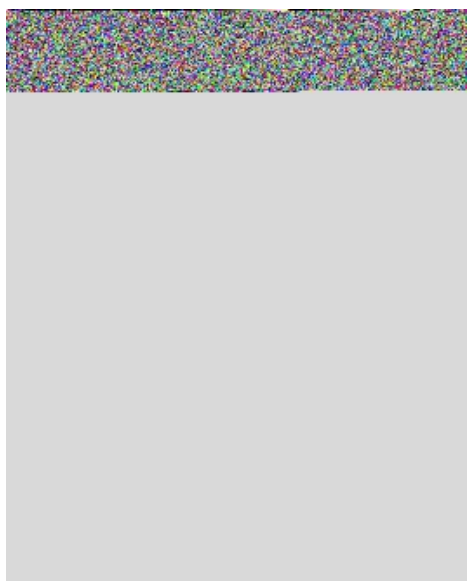 | 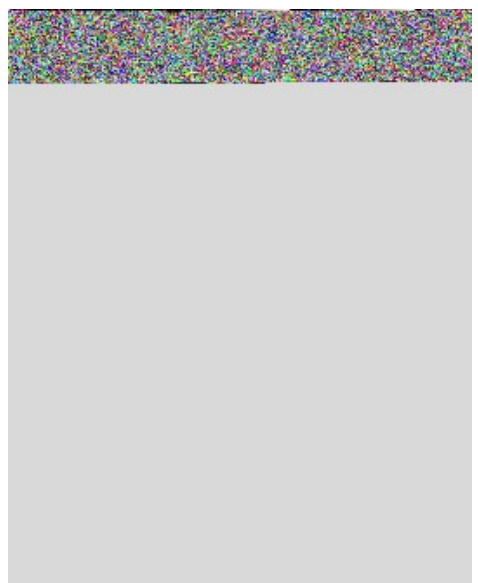 |
| TS13<br>$CH_2OH+H \rightarrow CH_3OH$ | 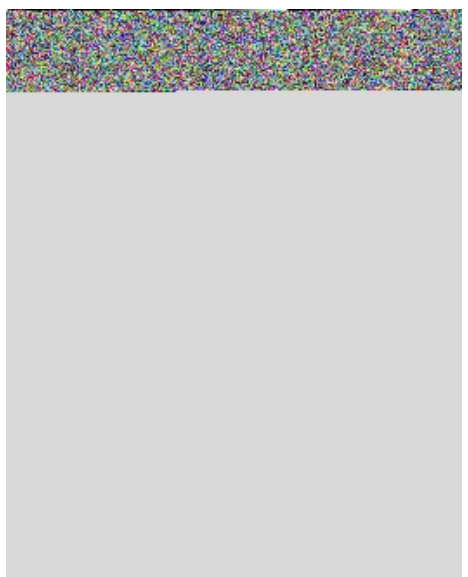 | 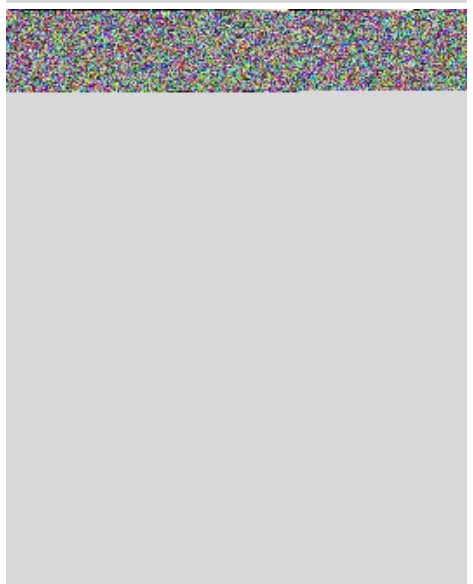 | 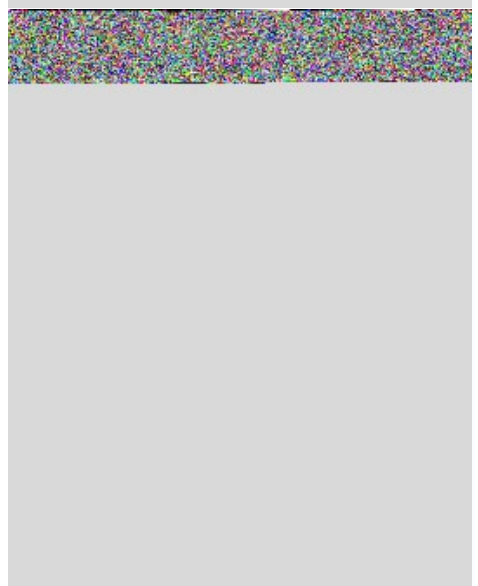 |
