# Supplementary material for: Theoretical insight into the effect of CO coverage on formic acid formation from dissociated oxygen on χ-Fe5C2(510) in Fischer–Tropsch synthesis
Source: RSC Adv. 2026 Jul 23. Online ahead of print. doi: 10.1039/d6ra04509j (PMC13392752; doi:10.1039/d6ra04509j)
Supplement: RA-OLF-D6RA04509J-s001 [file RA-OLF-D6RA04509J-s001.pdf]

Supplementary Information (SI) for RSC Advances.
This journal is © The Royal Society of Chemistry 2026

Theoretical Insight into the Effect of CO Coverage on Formic Acid Formation from Dissociated Oxygen on χ-F_e5C_2(510) in Fischer-Tropsch Synthesis

Jinchun Jiang [a], Hongzhi Zheng [a], Changyi Lai [a], qining Wang[b] Jie Ren [a], Ning Ai [a,b], Wei Zhou [c,*]

(a) College of Biological Chemical Science and Engineering, Jiaxing University,Jiaxing University, Jiaxing, China.

(b) National Demonstration Center for Experimental Chemistry and Chemical Engineering Education, Zhejiang University of Technology, Hangzhou China.

(c) Sichuan Technology and Business University, Chengdu,China;.

I. The detailed calculation formulas for DFT method

The adsorption energies E_{ads} (eV) are computed using Equation 1:

$$E_{ads} = E_{(adsorbate/slab)} - [E_{(slab)} + E_{(adsorbate)}] \quad (1)$$

Here, $E_{\text{(adsorbate/slab)}}$(eV), $E_{\text{(slab)}}$(eV), and $\mathrm{E}_{\text{(adsorbate)}}$(eV) denote the total energy of the adsorbate-surface system energy, the clean surface energy, and the isolated adsorbate energy, respectively.

The desorption energies E_{des} (eV) are computed using Equation 2:

$$E_{des} = E_{(desorbate/slab)} - [E_{(slab)} + E_{(desorbate)}] \quad (2)$$

Here, $E_{\text{(desorbate/slab)}}$(eV), $E_{\text{(slab)}}$(eV), and $\mathrm{E}_{\text{(desorbate)}}$(eV) denote the total energy of the desorbate-surface system energy, the clean surface energy, and the isolated desorbate energy, respectively.

To evaluate standard molar Gibbs free energies (*G*), The zero-point energy (*ZPE*) corrections, standard molar vibrational internal energy contributions, and vibrational entropy effects were applied, G could be computed via Equation 3:

$$G = E + ZPE + U^0 + \gamma RT(1 + ln\, {}^{p}\!/_{p^0}) - TS^0 \quad (3)$$

E denotes electronic energy, *ZPE* denotes zero-point energy, U^0 denotes standard molar vibrational internal energy at 0 K, p denotes system pressure, γ denotes the phase coefficient (0 for surfaces, 1 for gases), and S^0 standard molar vibrational entropy.

The standard molar Gibbs free energy (*G*) values are applied to calculate the Gibbs reaction energy (ΔG_r = *G*(FS) - *G*(IS)) and Gibbs barrier energy (ΔG_a = *G*(TS) - *G*(IS)), where *G*(IS), *G*(FS), and *G*(TS) denote the Gibbs free energies of the initial state, final state, and transition state, respectively.

This work addresses complex reaction network featuring extensive consecutive and parallel pathways. In consecutive steps, the coverage of intermediate species critically influences reaction rates according to law of mass action. Consequently, identifying rate-determining steps requires more than elementary

reaction energy barriers alone. We employ effective Gibbs barrier energies ($G_{a,eff}$) to account for reactant coverage effects on product formation rates[34-36], the $G_{a,eff}$ for the kth reaction step would be calculated via Equation 4:

$$G_{a,eff}^{\ k} = G_a^k + \sum_{i=j}^{k-1} G_r^i \qquad (4)$$

$G^k{}_a$(eV) and $G^i{}_r$(eV) represent the Gibbs barrier energy of the kth step and the Gibbs reaction energy of the ith step, respectively. The jth step reaction represented by j is the elementary step with the lowest product energy in a series of reactions.

This work defines the transitions of adsorbate between different stable adsorption states as migration reactions, which may involve one or multiple elementary reactions—each with distinct initial, transition, and final states. Computational analysis reveals minimal overall energy changes and low barriers in most migration processes, enabling rapid equilibrium attainment. Under such conditions, the Gibbs reaction energy rather than the Gibbs barrier energy is important. Hence, to prevent energy profile diagram becoming complex due to migration reactions, only the Gibbs reaction energy of migration reactions—defined as Gibbs migration energy (G_m)—were considered when evaluating migration effects on reaction pathways.

II . The detailed calculation formulas for kMC method

The reactant adsorption rate k_{ads} (1/s) is given by Equation 5:

$$k_{ads} = \frac{\sigma P A_{site}}{\sqrt{2\pi m k_B T}} \tag{5}$$

Here, σ denotes the dimensionless sticking coefficient (typically set to 1), P(Pa) and T(K) represent gas partial pressure and temperature under simulated conditions, A_{site}(m^2) is the per-site adsorption area, m(kg) is molecular mass, and K_B(J/K) is Boltzmann constant.

The forward rate constants k_i are calculated using the Arrhenius formulation in Equation 6, where A_i are the pre-exponential factor calculated via transition state theory in Equation 7**:**

$$k_i = A_i e^{-\frac{G_i}{K_B T}} \tag{6}$$

$$A_i = \frac{K_B T}{h} \frac{\prod_{i,TS} \frac{1}{1 - e^{-\frac{hv_i}{K_B T}}}}{\prod_{i,IS} \frac{1}{1 - e^{-\frac{hv_i}{K_B T}}}} \tag{7}$$

Here, k_B(J/K) denotes the Boltzmann constant, T(K) is the reaction temperature, h (J·s) is Planck's constant, vi^{TS}(m^{-1}) denotes the ith vibrational frequency of the transition state, and vi^{IS}(m^{-1}) is the corresponding vibrational frequency of the initial state

III. The optimal adsorption configurations of CO adsorption with different amount in Fig.2

1	2	3	4
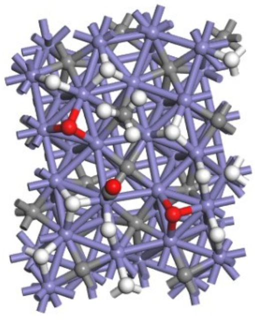	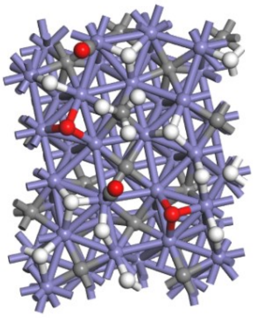	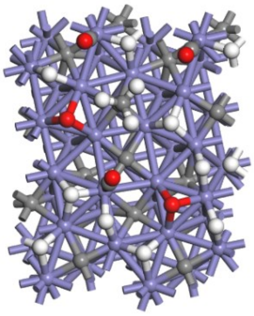	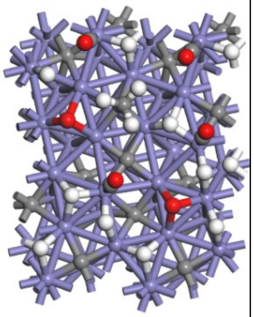
5	6	7	8
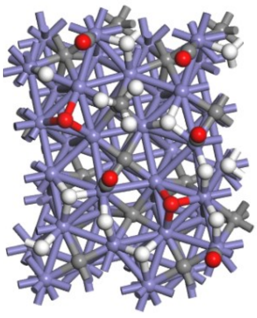	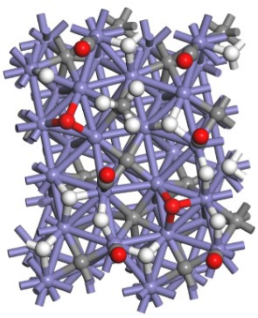	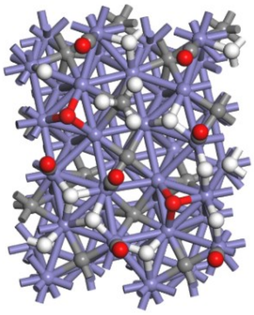	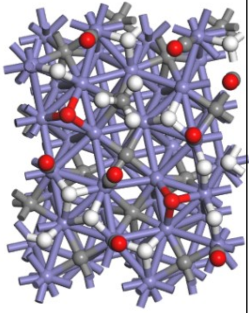
9	10	11	12

Reaction pathway	First step	Second step	Third step
1	$CO+O \rightarrow CO_2$	$CO_2+H \rightarrow CHOO$	$CHOO+ H \rightarrow HCOOH$
2	$CO+O \rightarrow CO_2$	$CO_2+H \rightarrow COOH$	$COOH+ H \rightarrow HCOOH$
3	$O+H \rightarrow OH$	$OH+CO \rightarrow COOH$	$COOH+ H \rightarrow HCOOH$
4	$CO+H \rightarrow CHO$	$O+H \rightarrow OH$	$CHO+OH \rightarrow HCOOH$
5	$CO+H \rightarrow CHO$	$COH+H \rightarrow CHOH$	$CHOH+O \rightarrow HCOOH$
6	$CO+H \rightarrow CHO$	$CHO+O \rightarrow CHOO$	$CHOO+H \rightarrow HCOOH$
7	$CO+H \rightarrow COH$	$COH+H \rightarrow CHOH$	$CHOH+O \rightarrow HCOOH$
8	$CO+H \rightarrow COH$	$COH+O \rightarrow COOH$	$COOH+ H \rightarrow HCOOH$

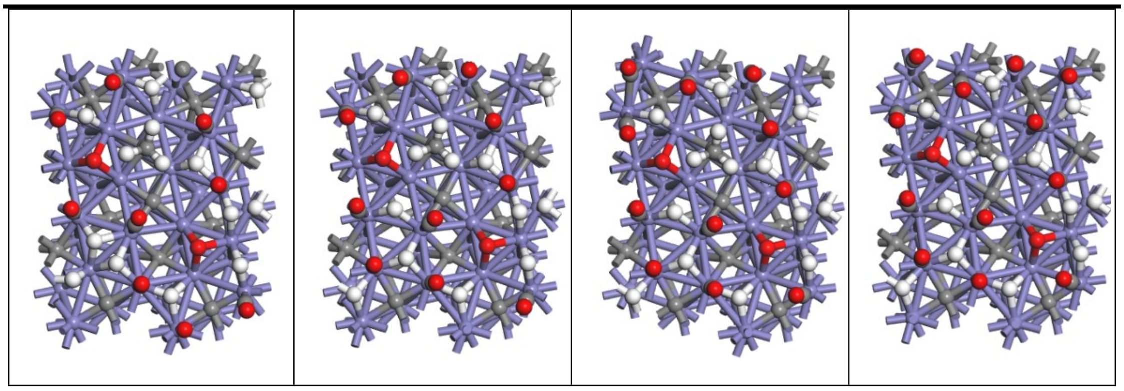

IV. The detailed elementary reactions involved in each route diagram of Fig.4

V. The detail configure of initial state(IS), translate state(TS) and final state(FS) for each elementary reaction in Fig5(a)

Elementary reaction	Initial state	Translate state	Final state
TS1<br>$CO+O \rightarrow CO_2$	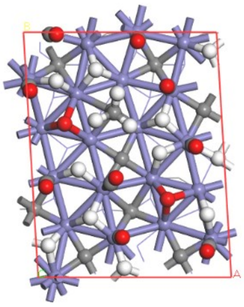	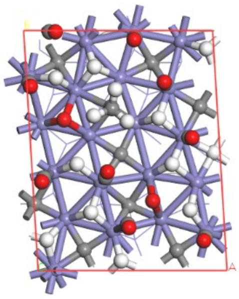	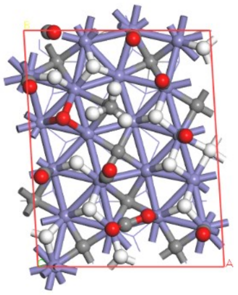
TS2<br>$CO+H \rightarrow CHO$	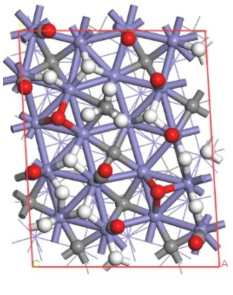	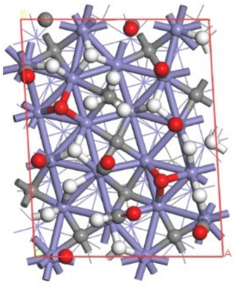	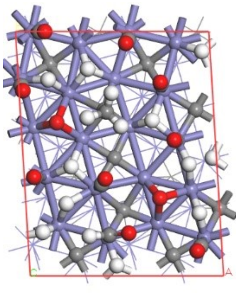

TS3

O+H→OH

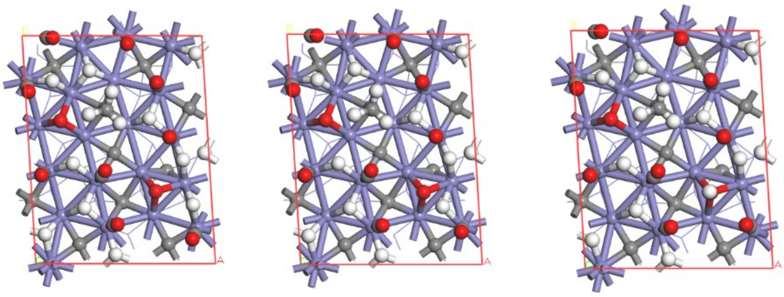

TS4

CO_2+H→CHOO

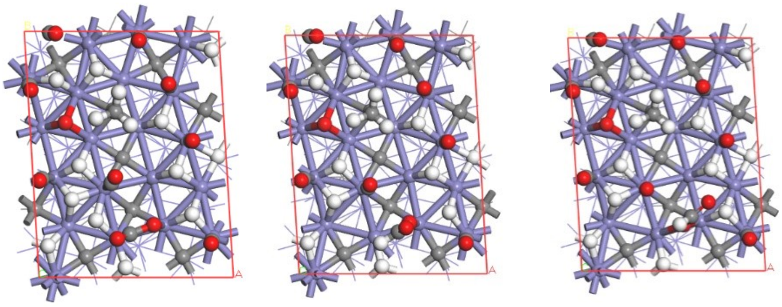

TS5

CHO_O+H→CHO_OH

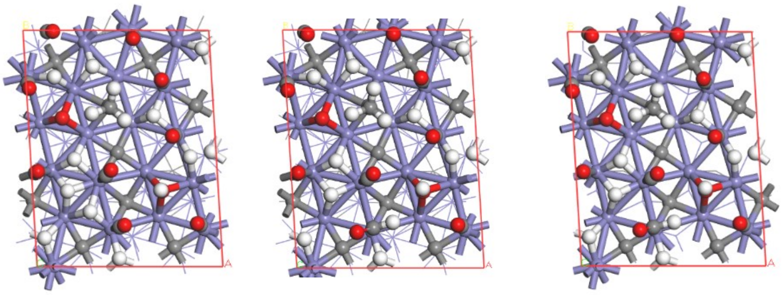

TS6

CO_2+H→COOH

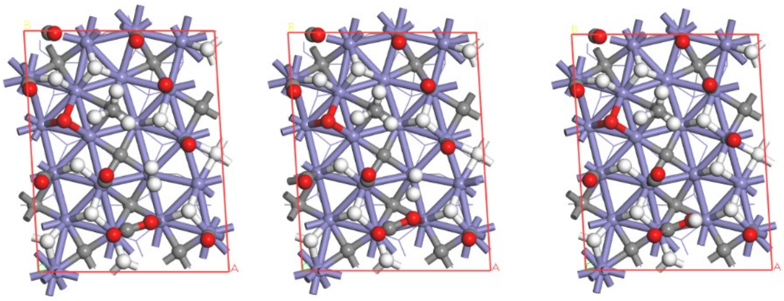

TS7

OH+CO→COOH

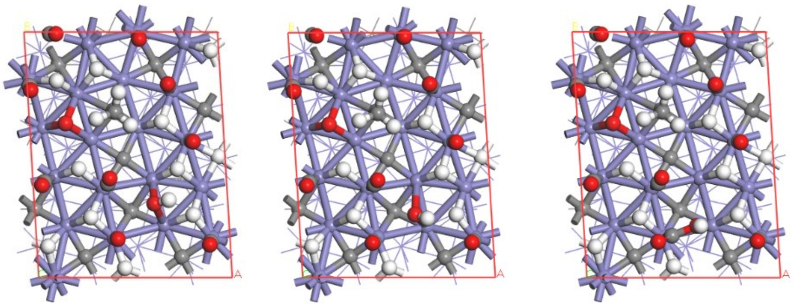

TS8
CHO+OH→HCOOH-1

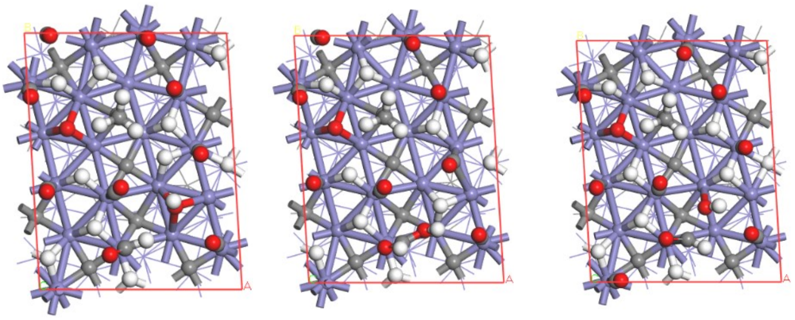

TS9
COOH+H→HCOOH-2

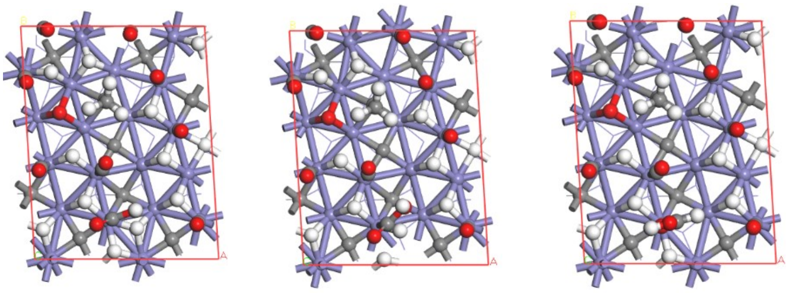

TS10
CHOO+H→HCOOH

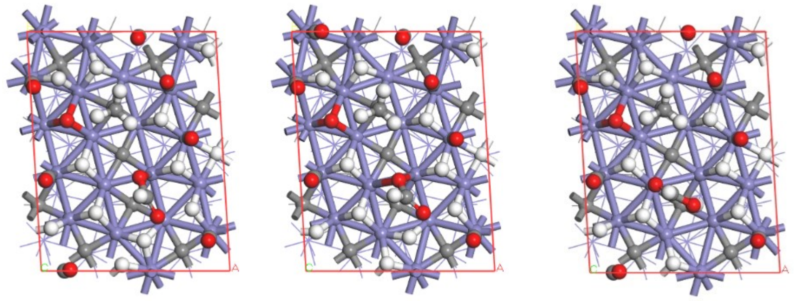

TS11
COOH+H→HCOOH-4

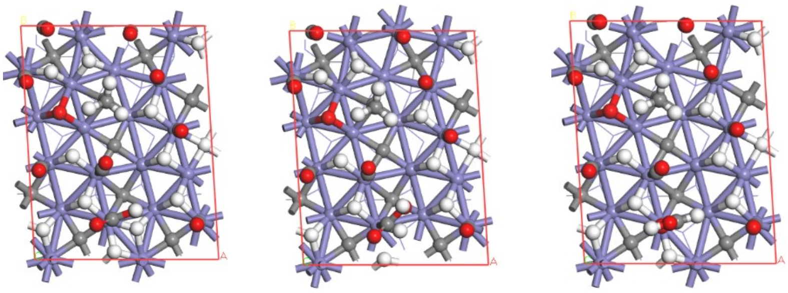

III. The detail configure of initial state(IS), translate state(TS) and final state(FS) for each elementary reaction in Fig5(b)

Elementary reaction	Initial state	Translate state	Final state
TS1<br>CO+H→CHO	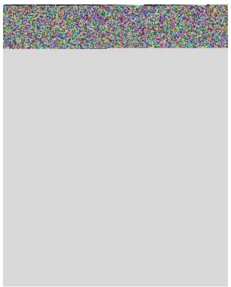	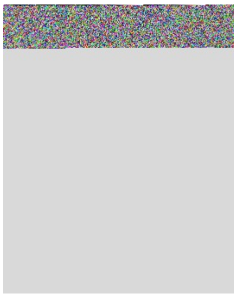	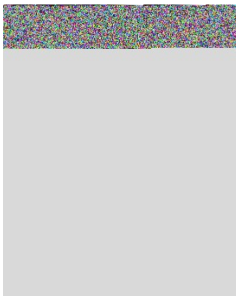
TS2<br>CO+H→COH	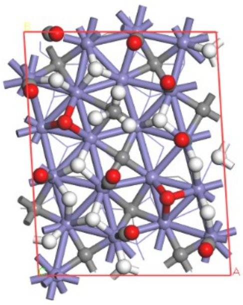	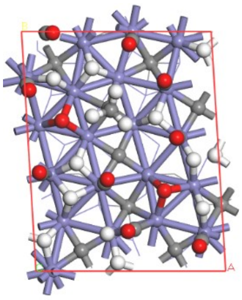	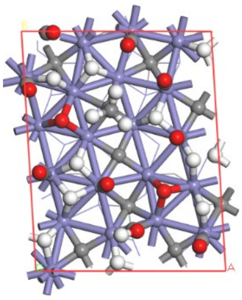

TS3

COH+O→COOH

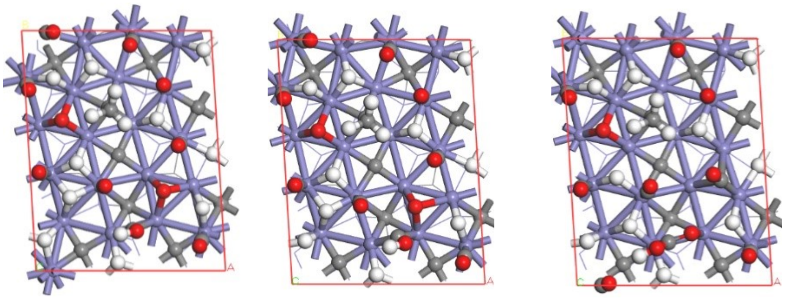

TS4

COH+H→CHOH

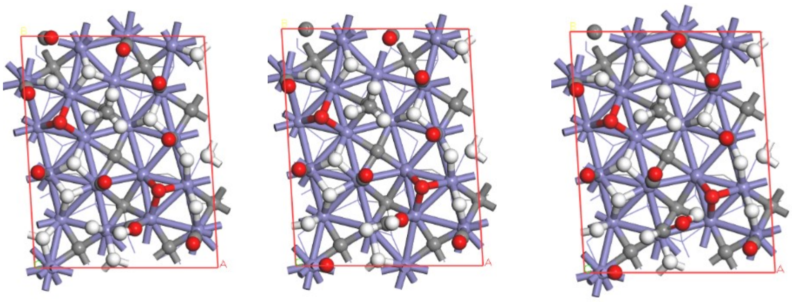

TS5

CHO+H→CHOH

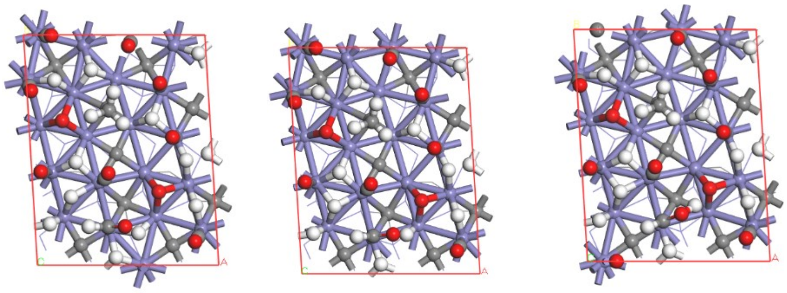

TS6

CHO+O→CHOO

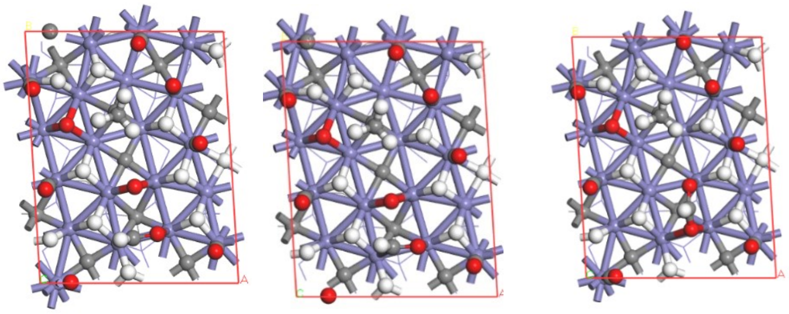

TS7

CHOH+O→HCOOH

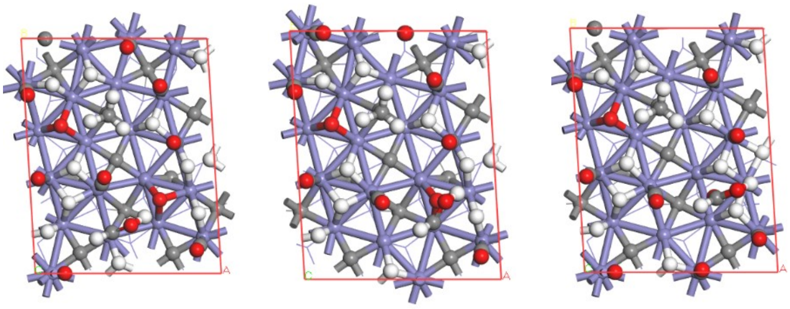

TS8<br>CHOH+O→HCOOH	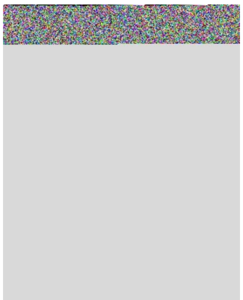	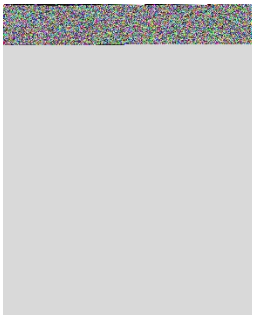	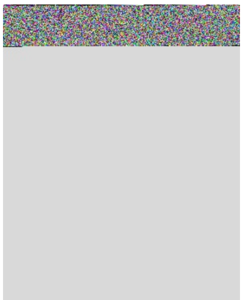
TS9<br>COOH+H→HCOOH	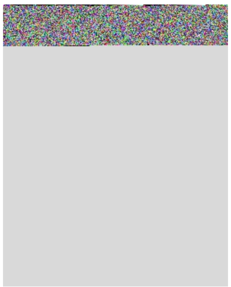	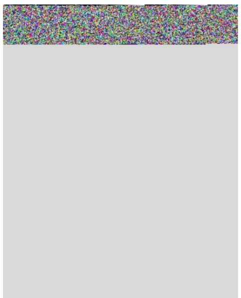	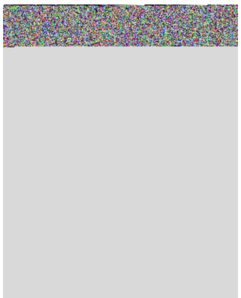
TS10<br>CHOO+H→HCOOH	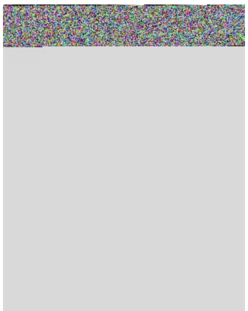	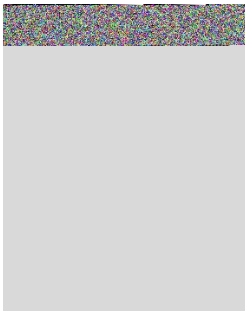	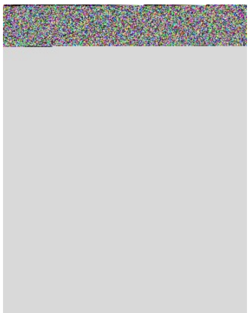

IV. The detail configure of initial state(IS), translate state(TS) and final state(FS) for each elementary reaction in Fig6(a)

Elementary reaction	Initial state	Translate state	Final state
TS1<br>CO+H→COH	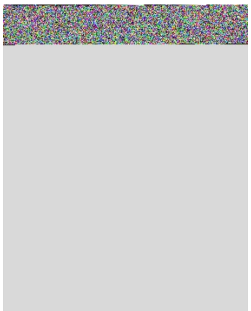	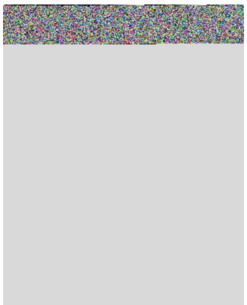	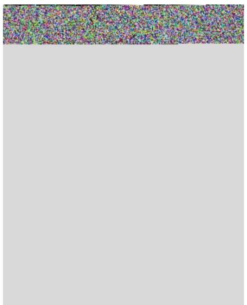

TS2

$CO+O \rightarrow CO_2$

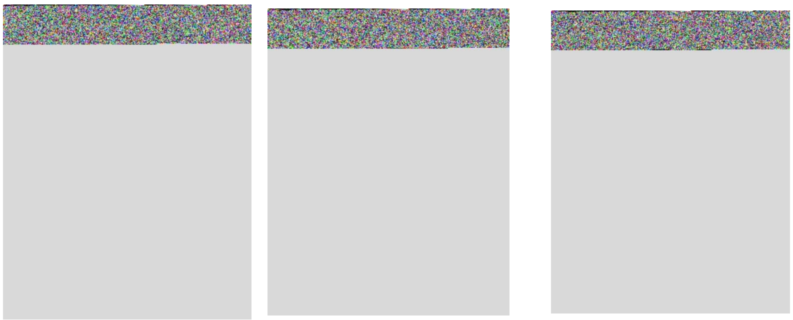

TS3

$CO+H \rightarrow CHO$

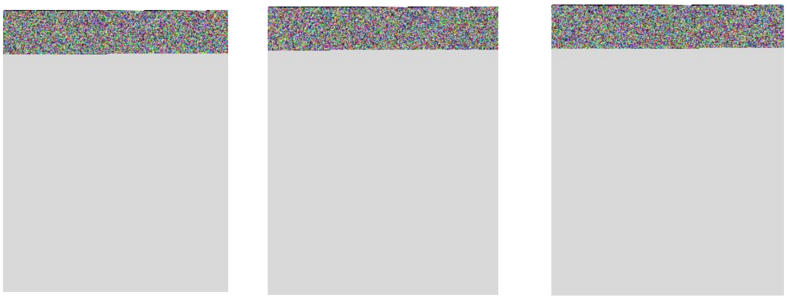

TS4

$O+H \rightarrow OH$

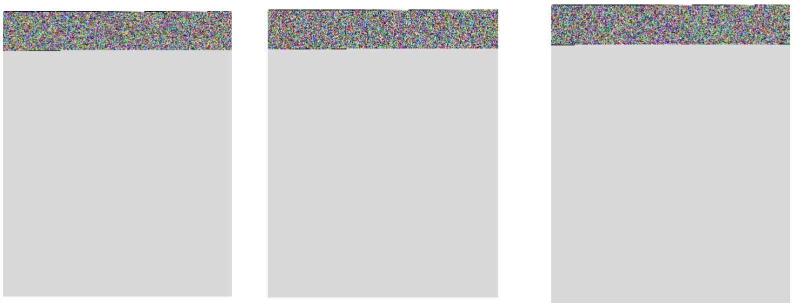

TS5

$COH+O \rightarrow COOH$

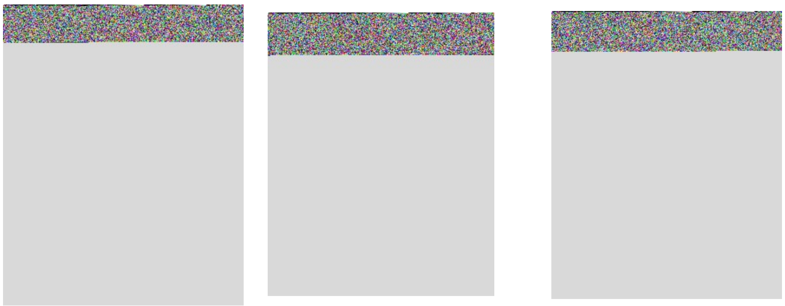

TS6

$CHO+O \rightarrow CHOO$

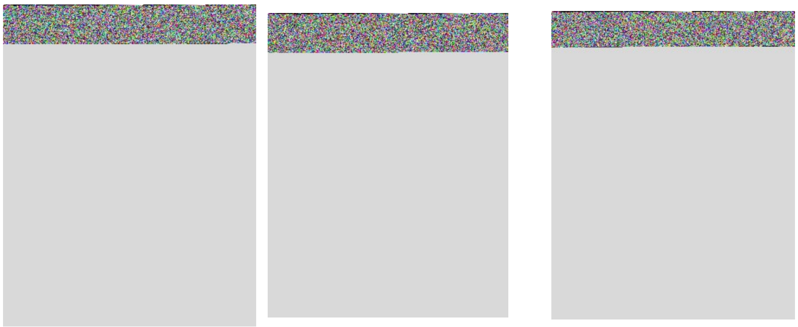

TS7<br>$OH+H \rightarrow H_2O$	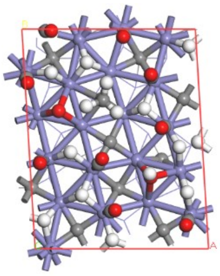	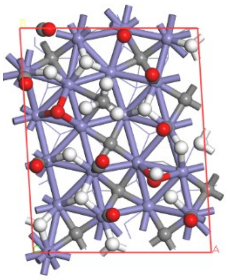	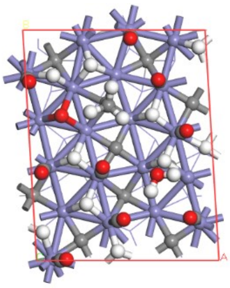
TS8<br>$CO+OH \rightarrow COOH$	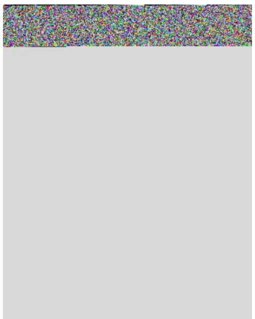	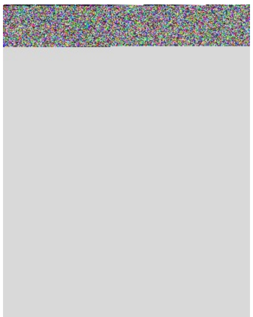	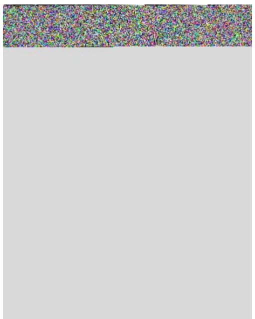
TS9<br>$CHOO \rightarrow CO_2+H$	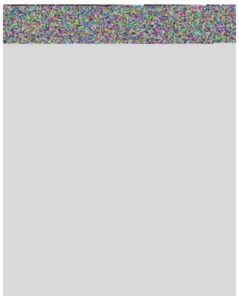	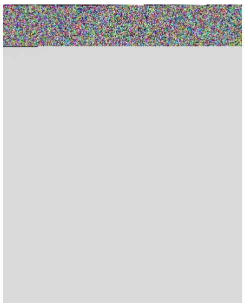	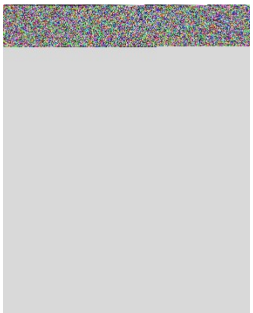
TS10<br>$COOH \rightarrow CO_2+H$	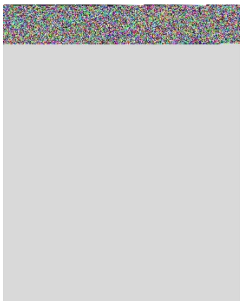	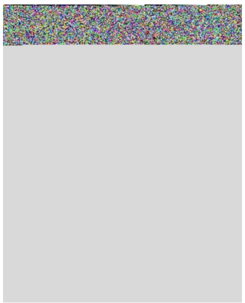	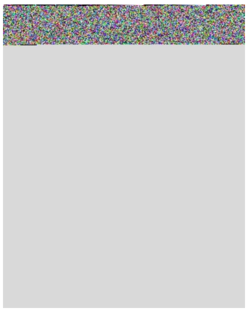
TS11<br>$COOH \rightarrow CO_2+H$	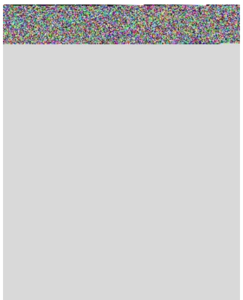	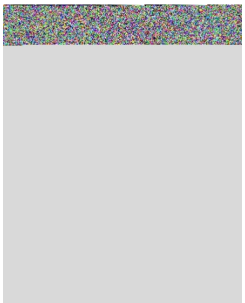	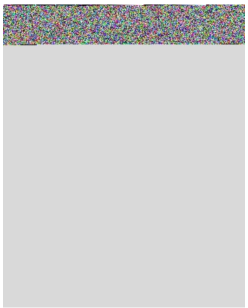

V. The detail configure of initial state(IS), translate state(TS) and final state(FS) for each elementary reaction in Fig6(b)

Elementary reaction	Initial state	Translate state	Final state
TS1<br>CO+H→COH	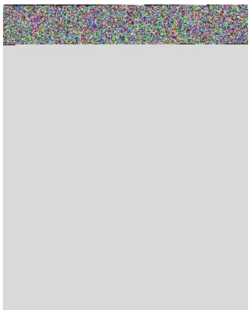	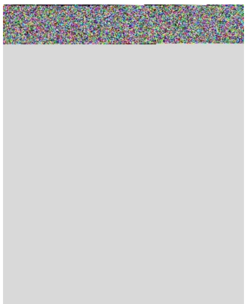	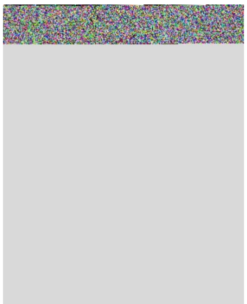

TS2

CO+H→CHO

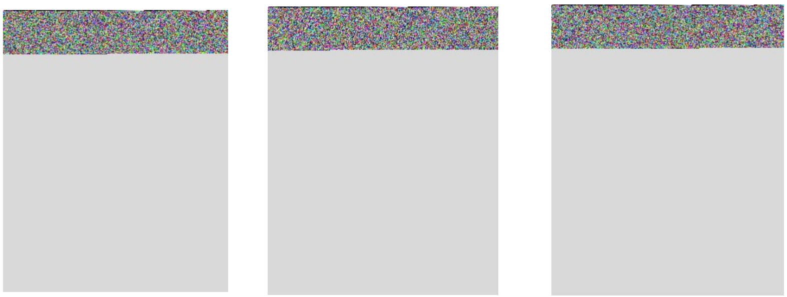

TS3

COH+H→CHOH

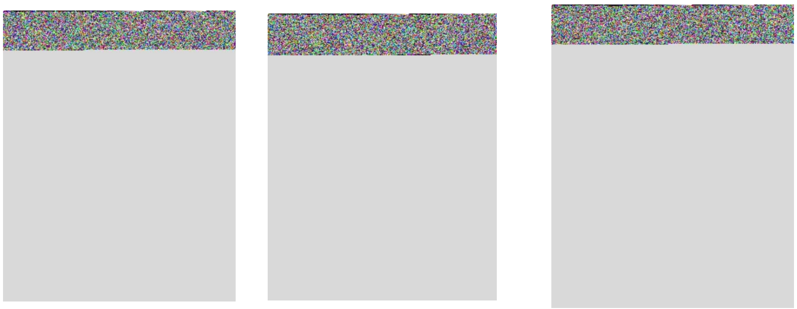

TS4

CHO+H→CHOH

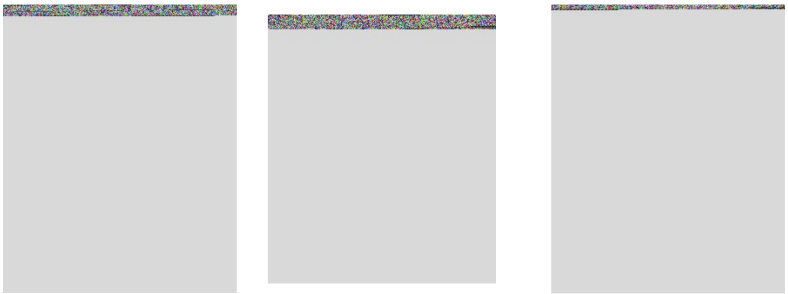

TS5

CHO+H→CH_2O

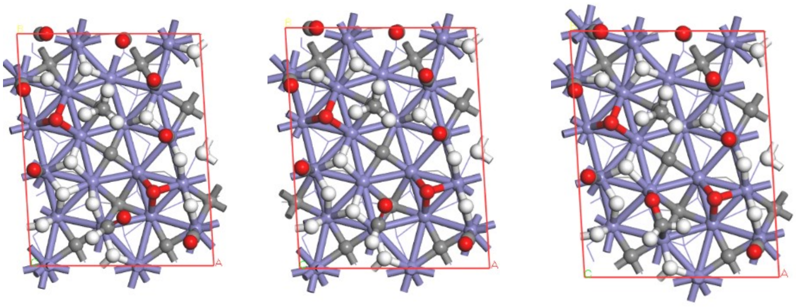

TS6

CH_2O+H→CH_2OH

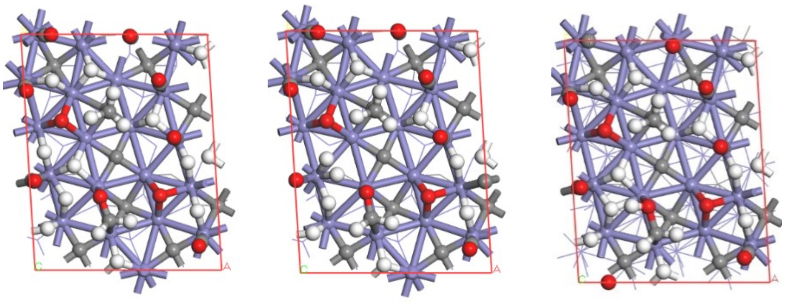

TS7

$CH_2O+H \rightarrow CH_2OH$

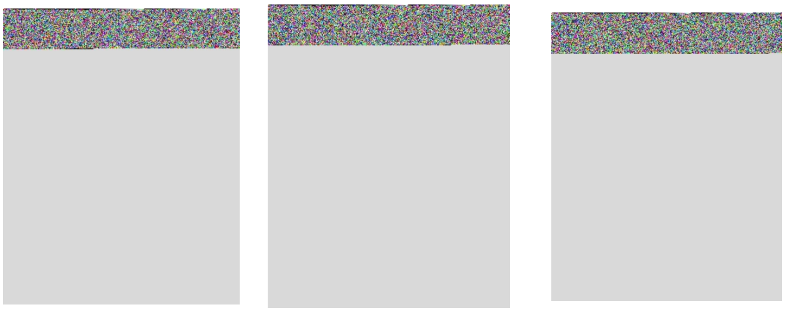

TS8

$CH_2O+H \rightarrow CH_2OH$

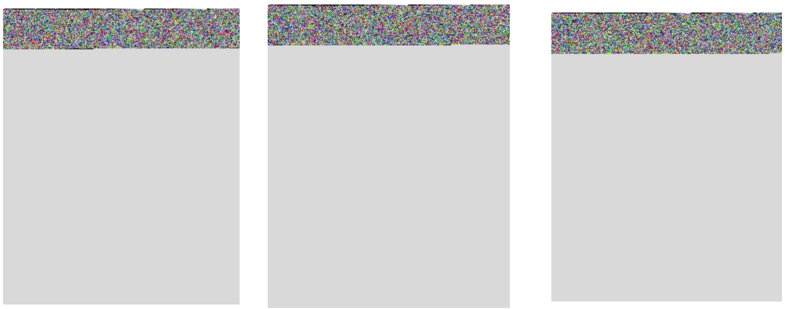

TS9

$CH_2O+H \rightarrow CH_3O$

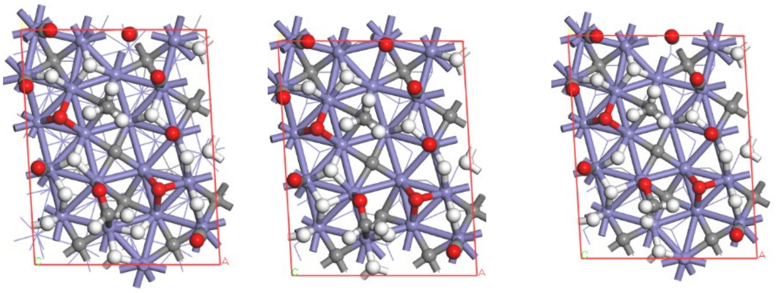

TS10

$CH_3O+H \rightarrow CH_3OH$

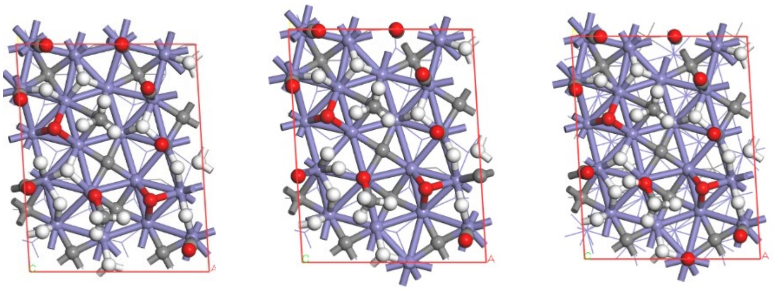

TS11

$CH_2OH+H \rightarrow CH_3OH$

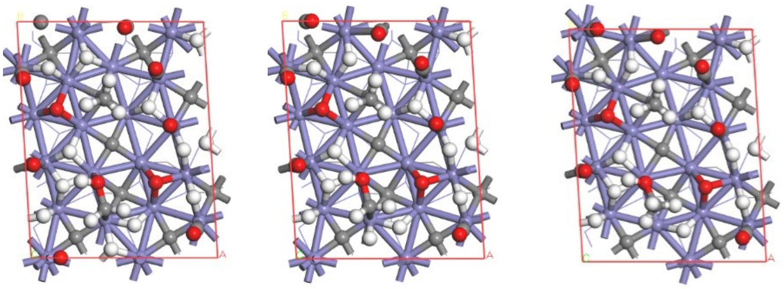

TS12<br>$CH_2OH+H \rightarrow CH_3OH$	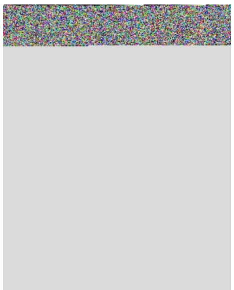	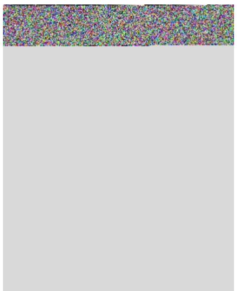	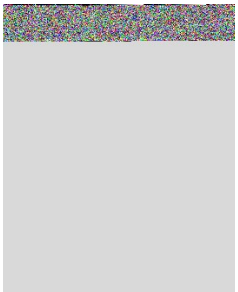
TS13<br>$CH_2OH+H \rightarrow CH_3OH$	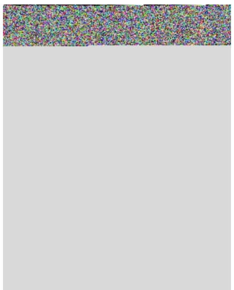	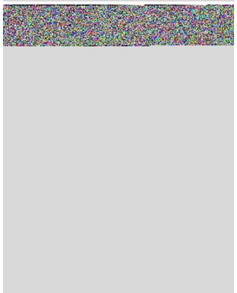	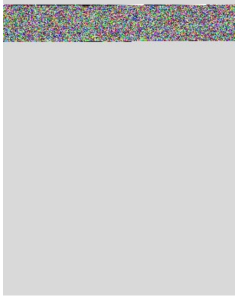
